# Expanding the base editing scope in rice by using Cas9 variants

**DOI:** 10.1111/pbi.12993

**Published:** 2018-10-05

**Authors:** Kai Hua, Xiaoping Tao, Jian‐Kang Zhu

**Affiliations:** ^1^ Shanghai Center for Plant Stress Biology CAS Center of Excellence in Molecular Plant Sciences Chinese Academy of Sciences Shanghai China; ^2^ University of Chinese Academy of Sciences Beijing China; ^3^ Department of Horticulture and Landscape Architecture Purdue University West Lafayette IN USA

**Keywords:** base editing, adenine base editor, cytosine base editor, Cas9 variants, rice

## Abstract

Base editing is a novel genome editing strategy that enables irreversible base conversion at target loci without the need for double stranded break induction or homology‐directed repair. Here, we developed new adenine and cytosine base editors with engineered SpCas9 and SaCas9 variants that substantially expand the targetable sites in the rice genome. These new base editors can edit endogenous genes in the rice genome with various efficiencies. Moreover, we show that adenine and cytosine base editing can be simultaneously executed in rice. The new base editors described here will be useful in rice functional genomics research and will advance precision molecular breeding in crops.

## Introduction

Base editing is a newly developed genome engineering tool that enables gene editing through irreversible base conversion, without the need for double stranded break (DSB) induction or homology‐directed repair (HDR; Komor *et al*., 2016). The first member in the base editing toolbox is the cytosine base editors (CBEs) that consist of a cytidine deaminase domain fused with a *Streptococcus pyogenes* Cas9 nickase [nSpCas9 (D10A)] or catalytically deficient Cas9 (dSpCas9; Komor *et al*., 2016; Nishida *et al*., 2016). CBEs can efficiently induce cytosine (C) to thymine (T) [or guanine (G) to adenine (A)] mutations at target sites in a wide range of species including plants (Li *et al*., 2017; Lu and Zhu, 2017). One major limitation of cytidine deaminase mediated base editing is its inability to induce other forms of base conversion beyond the C·G to T·A mutation. Recently, David R. Liu's group addressed this limitation by developing the adenine base editors (ABEs) that can convert A·T to G·C in a programmable manner in mammalian cells (Gaudelli *et al*., 2017). We and others showed that the ABEs can be adapted for applications in plants (Hua *et al*., 2018; Yan *et al*., 2018). The combination of adenine and cytosine base editors now can generate all four base transition mutations.

However, efficient adenine and cytosine base editing requires the presence of a specific protospacer adjacent motif (PAM) sequence (NGG PAM for SpCas9) that places the target base(s) within a narrow base editing window (Gaudelli *et al*., 2017; Komor *et al*., 2016). This PAM requirement significantly limits the genomic sites that can be targeted by the ABEs and CBEs. Here, we circumvent this limitation by developing new ABEs and CBEs with SpCas9 and *Staphylococcus aureus* Cas9 (SaCas9) variants, thus substantially increasing the target scopes for base editing in the rice genome.

## Results and discussion

Previous studies have shown that the wild type SpCas9 protein can recognize not only the canonical NGG PAM but also the non‐canonical NAG PAM sequence in the rice genome and exhibits robust editing efficiencies at some sites with NAG PAMs (Meng *et al*., 2018). We wondered whether our ABE‐P1 (adenine base editor plant version 1) vector with a wild type SpCas9 (D10A) nickase (Hua *et al*., 2018) can also edit genome sequences with NAG PAMs. First, we designed sgRNA1 that targeted the OsmiRNA156 binding sequence in *OsSPL14* followed by an NAG PAM (Figure S1a). From 46 independent transgenic lines generated from *Agrobacterium*‐mediated transformation, only two lines showed A‐G substitutions at the expected base editing window (Tables 1 and 2). Line SG1‐5 harboured an A‐G substitution at position 5 of the protospacer (scoring the NAG PAM as positions 21–23), whereas line SG1‐24 had an A‐G substitution at position 7 of the protospacer (T‐C conversions in opposite strand were shown in Figure S1b). To confirm the Sanger sequencing results, line SG1‐5 was selected for TA cloning. Three out of 20 randomly selected clones showed an A‐G substitution at position 5 of the protospacer, suggesting that line SG1‐5 was chimeric (T‐C conversions in opposite strand are shown in Figure S1c). To further test the base editing capability of ABE‐P1 at target sites with NAG PAMs, we designed two other sgRNAs. The second sgRNA (sgRNA2) was selected to simultaneously target OsmiRNA156 binding sites in *OsSPL16* and *OsSPL18* (Figure S1d). We genotyped 34 transgenic lines and found that only lines SG2‐15 and SG2‐18 had an A‐G base editing event at position 9 of the protospacer in the *OsSPL16* target site, whereas the target site in *OsSPL18* was not edited (Tables 1 and 2 and T‐C conversions in opposite strand are shown in Figure S1e). The third sgRNA (sgRNA3) was chosen to target the OsmiR396 binding site in *GRF4*. From the 40 transgenic lines that we obtained, we did not find any base editing event at this target site (Table 1). Taken together, the above results indicated that in contrast to the highly efficient cutting activity of the SpCas9 at NAG PAMs, the base editing efficiency of ABE‐P1 was low at target sites with NAG PAMs in rice.

**Table 1:** Summary of base editing efficiencies of different adenine base editors

Base editor	sgRNA^a^	Target gene	PAM sequence	Number of total transgenic lines	Number of edited lines	Editing efficiency
ABE‐P1	sgRNA1	*OsSPL14*	GAG	46	2	4.3%
	sgRNA2	*OsSPL16*	GAG	34	2	5.9%
		*OsSPL18*	GAG		0	0
	sgRNA3	*GRF4*	CAG	40	0	0
ABE‐P3	sgRNA4	*OsSPL14*	CGA	39	26	66.7%
		*OsSPL17*	GGA		29	74.3%
	sgRNA5	*OsSPL16*	GGA	30	9	30%
		*OsSPL18*	GGA		20	66.7%
ABE‐P4	sgRNA6	*OsTOE1*	AGCG	39	1	2.6%
		*OsIDS1*	AGCG		1	2.6%
	sgRNA7	*OMTN1*	GGCG	8	0	0
ABE‐P5	sgRNA8	*SNB*	TGCAGT	46	3	6.5%
	sgRNA9	*OsSPL13*	TTAGGT	21	0	0

a Two genomic sites are simultaneously targeted by sgRNA2, sgRNA4, sgRNA5 and sgRNA6.

**Table 2:** Base editing activity window for different ABEs and CBEs at different target sites

Base editor	sgRNA^a^	Target gene	Base editing sites in the protospacer^b^	Editing form
ABE‐P1	sgRNA1	*OsSPL14*	5,7	A‐G conversion
	sgRNA2	*OsSPL16*	9	A‐G conversion
ABE‐P2	sgRNA12	*OsSPL14*	8,10,14	A‐G conversion
		*OsSPL17*	6,8,10,14	A‐G conversion
ABE‐P3	sgRNA4	*OsSPL14*	3,4,6,8,10	A‐G conversion
		*OsSPL17*	4,6,8,10	A‐G conversion
	sgRNA5	*OsSPL16*	6,8	A‐G conversion
		*OsSPL18*	3,4,6,8,10	A‐G conversion
ABE‐P4	sgRNA6	*OsTOE1*	5	A‐G conversion
		*OsIDS1*	5	A‐G conversion
ABE‐P5	sgRNA8	*SNB*	4,8	A‐G conversion
CBE‐P1	sgRNA13	*SNB*	4,5	C‐T conversion
CBE‐P3	sgRNA11	*PMS3*	8,11,15	C‐T conversion

a Two genomic sites are simultaneously targeted by sgRNA2, sgRNA4, sgRNA5, sgRNA6 and sgRNA12.

b Base editing sites in the protospacer were counted from the PAM‐distal end, scoring the PAM as positions 21–23 for base editors with SpCas9 variants and scoring the PAM as positions 22–27 for base editors with SaCas9 variants.

**Figure 1:**
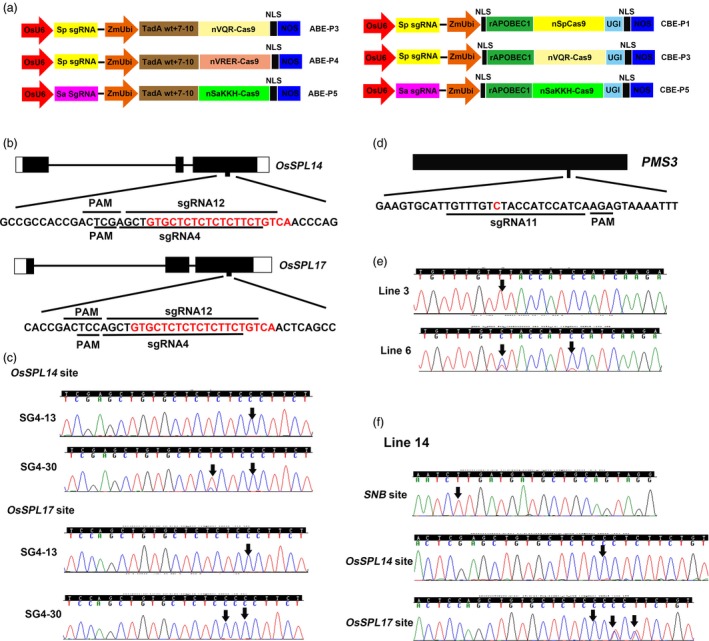
Base editing with new base editors in rice. (a) Schematics of new adenine and cytosine base editors used in this study. (b) Schematic view of the sgRNA4 and sgRNA12 target sites in the *OsSPL14* and *OsSPL17* genes. The OsmiR156 binding sequences of *OsSPL14* and *OsSPL17* are highlighted in red letters. (c) Sequencing chromatograms at the *OsSPL14* and *OsSPL17* target sites of two representative edited lines, SG4‐13 and SG4‐30. Arrows point to the positions with edited bases. (d) Schematic view of the sgRNA11 target site in *PMS3*. The targeted cytosine for base editing is highlighted in red. (e) Representative Sanger sequencing chromatograms of CBE‐P3‐edited *PMS3* alleles. The edited bases are marked by arrows. (f) One representative transgenic line (line 14) is simultaneously edited at three target sites by the ABE‐P2 and CBE‐P1. Sanger sequencing chromatograms at the three target sites are shown. Arrows point to the positions with edited bases.

To expand the targeting scope of our ABE‐P1 base editor in rice, we sought to replace the wild type SpCas9 in ABE‐P1 with engineered SpCas9 variants that can recognize alternative PAM sequences. We used nickases VQR‐Cas9 (D10A) and VRER‐Cas9 (D10A) to replace the SpCas9 (D10A) nickase in the ABE‐P1 base editor, leading to the base editors ABE‐P3 and ABE‐P4, respectively (Figure 1a). The VQR‐Cas9 was reported to recognize an NGA PAM sequence, whereas the VRER‐Cas9 could accept an NGCG PAM sequence (Kleinstiver *et al*., 2015b). For the base editor ABE‐P3, we designed two sgRNAs to test its base editing efficiency. sgRNA4 was selected to target the OsmiRNA156 binding sites in *OsSPL14* and *OsSPL17* (Figure 1b). The base editing efficiency at the *OsSPL17* target site was 74.3% (29/39), slightly higher than that at the *OsSPL14* target site (66.7%, 26/39; Table 1). Importantly, nearly 66.7% (26/39) transgenic lines were edited at both target sites. Among them, line SG4‐30 was homozygous at both target sites (Figure 1c. It is noteworthy that adenines in positions 3, 4, 6, 8, 10 of the protospacer could be substituted to guanines in the *OsSPL14* and/or *OsSPL18* target sites (Table 2). sgRNA5 was designed to target the OsmiRNA156 binding sites in *OsSPL16* and *OsSPL18* (Figure S2a). The base editing efficiencies at these two target sites were also high, up to 66.7% (20/30) at the *OsSPL18* target site and 30% (9/30) at the *OsSPL16* target site (Table 1). Furthermore, nine transgenic lines were edited simultaneously at both sites and sequencing chromatograms of two representative lines are shown in Figure S2b. Interestingly, even adenine in position 10 of the protospacer could be efficiently edited at the *OsSPL18* target site (Table 2).

For the base editor ABE‐P4, we tested two sgRNAs (sgRNA6 and sgRNA7) that targeted three different genomic sites. We found that the base editing efficiencies of ABE‐P4 at the selected target sites were much lower than that of ABE‐P3 (Table 1). The miR172 binding sites in *OsIDS1* and *OsTOE1* were selected as target sites of sgRNA6 (Figure S3a). From 39 transgenic lines that we genotyped, only one line (SG7‐9) was found to harbour an expected A‐G substitution at position 5 of the protospacer in *OsIDS1*, while another line, SG7‐17, had an A‐G substitution at position 5 of the protospacer in *OsTOE1* (T‐C conversions in opposite strand are shown in Figure S3b and Table 2). We did not detect any mutations in the OsmiR164 binding site in the *OMTN1* gene, which was targeted by sgRNA7 (Table 1).

Recently, studies have also shown that an engineered SaCas9 variant (SaKKH‐Cas9) can recognize a more relaxed PAM sequence, NNNRRT (Kleinstiver *et al*., 2015a). To broaden the target range of our ABE‐P2 base editor that uses SaCas9 (Hua *et al*., 2018), we constructed a base editor, ABE‐P5, which has a SaKKH‐Cas9 (D10A) nickase to replace the SaCas9 (D10A) nickase in ABE‐P2 (Figure 1a). We designed two sgRNAs (sgRNA8 and sgRNA9) to test the activity of ABE‐P5 against endogenous genes in the rice genome. For sgRNA8 that targets the OsmiR172 binding site in the *SNB* gene, the base editing efficiency was 6.5% (3/46; Figure S4a,b and Table 1). We did not find any mutation for sgRNA9 that targets the OsmiR156 binding site in *OsSPL13* (Table 1).

Cytosine base editors with SpCas9 and SaCas9 variants have been shown to work efficiently in mammalian cells (Kim *et al*., 2017). So we sought to expand the target range of the cytosine base editors in plants. Previous studies in rice have identified two genetic loci, *PMS1* and *PMS3*, that are responsible for the environment sensitive genic male sterity (EGMS) phenotype of the NK58S mutant and both loci encode long noncoding RNAs (Fan and Zhang, 2018). The causal mutations in NK58S were G‐T and C‐G substitutions in *PMS1* and *PMS3,* respectively (Fan and Zhang, 2018). We surmised that a G‐A mutation in *PMS1* and a C‐T substitution in *PMS3* may also lead to the same EGMS phenotype in the rice variety Nipponbare. We designed sgRNA10 for CBE‐P5 that targets *PMS1* and sgRNA11 for CBE‐P3 that targets *PMS3* (Figure 1a,d and Figure S5b). These two vectors were then separately transformed into rice by *Agrobacterium*‐mediated transformation or co‐transformed by particle bombardment. From the single transformation experiment, five lines were edited at the *PMS3* target site from just seven transgenic lines generated (Figure 1e and Table 3). However, we did not find any mutation at the *PMS1* target site from 52 transgenic lines (Table 3). From the co‐transformation experiment, 18 co‐transformants were obtained from 28 transgenic lines. Again, we found that the *PMS3* target site was edited with high efficiency in the co‐transformants, up to 61.1% (11/18; Table 3). All edited lines had expected C‐T substitutions, except that one line had an additional 1 bp insertion. We found that cytosines even at position 15 in the protospacer could be edited (Figure 1e and Table 2). Therefore, the base editing windows for ABEs and CBEs at some target sites in our study (Table 2) are larger than previously defined in mammalian systems (Gaudelli *et al*., 2017; Komor *et al*., 2016). Two recent *in vivo* base editing studies in mouse and rabbit also showed that ABEs and CBEs with SpCas9 can edit target adenines and cytosines outside of the canonical base editing activity windows at some target sites (Liu *et al*., 2018a,b). As the base editing windows characterized in previous studies were derived from limited target sites and were mainly from cell‐based assays which had relatively a short time for base editors to function (Gaudelli *et al*., 2017; Komor *et al*., 2016), more target sites need to be tested, and *in vivo* base editing studies are required to accurately define the base editing windows for both adenine and cytosine base editors. Furthermore, it has been reported that base editors with SaCas9 have larger base editing activity windows than those of base editors with SpCas9, perhaps due to greater strand exposure to deaminases after formation of the SaCas9‐sgRNA‐DNA R‐loop complex (Hua *et al*., 2018; Kim *et al*., 2017).

**Table 3:** Base editing or mutation efficiencies of sgRNA10‐sgRNA13

sgRNA	Base editor/vector	Target gene	Single or double transfor‐mation^a^	Number of transgenic lines	Number of co‐transfor‐mants	Number of edited lines	Editing efficiency
sgRNA10	CBE‐P5	*PMS1*	single	52	–	0	0
sgRNA11	CBE‐P3	*PMS3*	single	7	–	5	71.4%
sgRNA10	CBE‐P5	*PMS1*	double	28	18	0	0
sgRNA11	CBE‐P3	*PMS3*	double	28	18	11	61.1%
sgRNA10	pSaKKH‐Cas9	*PMS1*	double	19	14	5	35.7%
sgRNA11	pVQR‐Cas9	*PMS3*	double	19	14	0	0
sgRNA12	ABE‐P2	*OsSPL14*	double	51	20	5	25%
		*OsSPL17*	double	51	20	9	45%
sgRNA13	CBE‐P1	*SNB*	double	51	20	16	80%

a Double transformation means that two vectors are co‐transformed into rice by particle bombardment.

It was interesting that the *PMS1* target site was still resistant to editing in the co‐transformants, indicating that sgRNA10 had poor activity. The sgRNA activity is affected by many factors, such as base composition, GC content and chromatin states of the target site. To test the activities of sgRNA10 and sgRNA11, we also co‐transformed rice with the corresponding DSB‐inducing CRISPR/Cas9 vectors (pSaKKH‐Cas9 and pVQR‐Cas9) as a control (Figure S5a). To our surprise, this time we found that five lines were mutated at the sgRNA10 target site from 14 co‐transformants, whereas no mutations were identified at the sgRNA11 target site (Figure S5c and Table 3). Therefore, the sgRNA activities for inducing indel mutations and for base editing are not correlated, at least at the *PMS1* and *PMS3* target sites. We speculate that a mechanistic difference between CRISPR‐Cas9 mediated mutagenesis and base editing may account for their different mutation rates at the *PMS1* and *PMS3* target sites. The key mechanistic difference between CRISPR‐Cas9 mediated mutagenesis and base editing is whether or not they rely on DSB formation. The DSB induced by Cas9 is mainly repaired by the non‐homologous end‐joining pathway in plant cells, which usually results in indel mutations (Michael and Holger, 2017). However, the base editors do not induce DSB formation and are designed to manipulate the cellular DNA repair pathways by inhibiting base‐excision repair and stimulating mismatch repair to improve base conversion efficiency (Gaudelli *et al*., 2017; Komor *et al*., 2016). Thus, different DNA repair pathways are involved in indel formation and base conversion. It is possible that the sequence context and/or other features of a target site may determine whether one repair pathway is more efficient than the other at the target site. The different mutation rates between CRISPR‐Cas9 mediated mutagenesis and base editing at the same target site may be a reflection of this difference.

Thus far, multiple Cas9 orthologues have been identified and repurposed for genome engineering. We hypothesized that the adenine and cytosine base editing may be simultaneously executed in plants by fusing the cytidine and adenine deaminase to orthogonal Cas9 enzymes. To test our hypothesis, we designed sgRNA12 for ABE‐P2 and sgRNA13 for CBE‐P1. sgRNA12 targets the OsmiR156 binding sites in *OsSPL14* and *OsSPL17* (Figure 1b), whereas sgRNA13 targets the OsmiR172 binding site in *SNB* (Figure S4a). The two vectors were co‐transformed into rice by particle bombardment. After genotyping, we obtained 20 co‐transformants from 51 transgenic lines (Table 3). Then the three target sites in the 20 co‐transformants were individually PCR amplified and subjected to Sanger sequencing. We found that 5 and 9 lines harboured the expected A‐G substitutions at the target sites in *OsSPL14* and *OsSPL17*, respectively (Table 3). Eighty percent of the co‐transformants (16/20) had C‐T substitutions at the target site in *SNB* without inducing any indels or other base transition or transversion mutations (Table 3). Importantly, we obtained 5 lines that were edited simultaneously at the three target loci. Sequencing chromatograms at the three target sites of a representative line, line 14, are shown in Figure 1f. These results suggested that the ABE and CBE can efficiently work together in plants.

In summary, we have developed new adenine and cytosine base editors with engineered SpCas9 and SaCas9 variants that substantially expand the targetable sites in the rice genome. Moreover, we have demonstrated that adenine and cytosine base editing can be simultaneously executed in rice. We anticipate that our new base editors described here will be useful for molecular genetics research and precision molecular breeding in rice and other crops.

## Experimental procedures

### Vector construction

Adenine base editing vectors with SpCas9 and SaCas9 variants used in this study were modified from the pRABEsp‐OsU6 (also named as ABE‐P1) and pRABEsa‐OsU6sa (also named as ABE‐P2) vectors (Hua *et al*., 2018). Briefly, specific point mutations described by Kleinstiver *et al*. (2015a,b) were introduced into the wild type SpCas9 and SaCas9 by PCR to convert them to VQR‐Cas9, VRER‐Cas9 and SaKKH‐Cas9 variants. The nickases VQR‐Cas9 (D10A) and VRER‐Cas9 (D10A) were then used to replace the SpCas9 (D10A) nickase in the pRABEsp‐OsU6 vector by the Gibson assembly method, leading to the vectors pRABEsp‐VQR (ABE‐P3) and pRABEsp‐VRER (ABE‐P4), respectively. In the same way, the SaCas9 (D10A) nickase in the pRABEsa‐OsU6sa vector was replaced by the SaKKH‐Cas9 (D10A) nickase, resulting in the vector pRABEsa‐SaKKH (ABE‐P5).

For the cytosine base editing vectors, the cytidine deaminase rAPOBEC1 with a XTEN linker and the uracil glycosylase inhibitor UGI with a VirD2 nuclear localization signal (NLS) were synthesized by Sangon Biotech (Shanghai, China) as described by Komor *et al*. (2016). The three fragments, rAPOBEC1‐XTEN linker, SpCas9 (D10A) or VQR‐Cas9 (D10A) nickase, UGI‐VirD2 NLS, were assembled into the backbone of the pRABEsp‐OsU6 vector to replace the ABE7‐10 cassette, leading to the vectors pRCBEsp‐OsU6 (CBE‐P1) or pRCBEsp‐VQR (CEB‐P3). The pRCBEsa‐SaKKH vector (CBE‐P5) was constructed in a similar way by assembling rAPOBEC1‐XTEN linker, SaKKH‐Cas9 (D10A) nickase and UGI‐VirD2 NLS into the backbone of the pRABEsa‐OsU6sa vector.

For the DSB inducing CRISPR/Cas9 vectors, the ABE7‐10 cassettes in the pRABEsp‐OsU6 and pRABEsa‐OsU6sa vectors were replaced by the VQR‐Cas9 and SaKKH‐Cas9 variants, leading to the vectors pVQR‐Cas9 and pSaKKH‐Cas9, respectively. Complete DNA sequences of all vectors used in this study are provided in Data S1.

Primers for sgRNAs were synthesized and annealed on a PCR machine. The annealed oligo adaptors were then inserted into the BsaI digested binary vectors by standard molecular cloning methods. The accuracy of all vectors was confirmed by Sanger sequencing before rice transformation. A full list of primers for sgRNAs used in this study is shown in Table S1.

### Rice transformation

*Agrobacterium*‐mediated rice transformation was performed as described with minor modifications (Nishimura *et al*., 2007). The binary vectors were first introduced into the *Agrobacterium tumefaciens* strain EH105 by the freeze/thaw method. Embryogenic calli induced from mature seeds of rice variety Nipponbare (*Oryza sativa* L. *japonica. cv*. Nipponbare) were used for transformation. Two days after *Agrobacterium* infection at 22°C in the dark, rice calli were transferred to selection medium with 50 mg/L hygromycin for one round selection (about 2 weeks) in the dark. Then, the hygromycin resistant calli were directly transferred to regeneration medium for 1 month to induce shoot regeneration. The shoots that grew up to 4–5 cm length were transferred to rooting medium for root induction. Two weeks later, the plantlets were transplanted to greenhouse and grew under the standard conditions for rice (12‐h light 28°C and 12‐h darkness at 22°C).

For particle bombardment‐mediated co‐transformation, two vectors were pre‐mixed together in a 1:1 ratio. Then the vectors were coated on 0.6 μm gold particles, following the protocol described by Shan *et al*. (2014). Before bombardment, rice calli were pre‐treated on osmotic medium (induction medium with 0.5 mol/L mannitol) for four hours. Particle bombardments were performed on PDS‐1000/He™ system (Bio‐Rad, California) according to the manufacturer's instructions. After bombardment, rice calli were kept on osmotic medium for another eighteen hours under the dark condition at 28°C. Thereafter, the rice calli were transferred to the selection medium for two rounds selection. Then the hygromycin resistant calli were selected for regeneration. The shoot regeneration and root induction steps were the same as described above.

### Genotyping base editing and mutation events

Rice genome DNA was extracted from the fresh leaves of all transgenic lines. The target regions were amplified by *Taq* DNA polymerase and the PCR products were send for Sanger sequencing by a nest primer. For base edited lines that need to be confirmed by TA cloning, the target regions were re‐amplified by *KOD* DNA polymerase (TOBOYO, OSAKA, Japan) and the PCR products were cloned into the p‐EASY Blunt Zero vector (TransGen Biotech, Beijing, China). At least 20 clones were randomly selected for sequencing. For rice co‐transformation experiments, all transgenic lines were first genotyped with the M13F and the corresponding sgRNA reverse primers. Then the co‐transformants were selected for sequencing at the target sites. Base editing ratio at each target site was calculated by dividing the number of plants with base editing events to the total number of genotyped transgenic lines. All the primers used for genotyping are listed in Table S2.

## Conflict of interest

The authors declare no conflict of interests.

## Author contributions

K.H. performed most of the experiments, analysed the data and wrote the manuscript. X.T. performed rice transformation. J.‐K. Z. supervised the project and wrote the manuscript.

## Supporting information


**Data S1** Complete DNA sequences of all vectors used in this study.
**Figure S1** Base editing by ABE‐P1 at the genomic sequences with NAG PAMs.
**Figure S2** Targeted base editing at *OsSPL16* and *OsSPL18* by ABE‐P3.
**Figure S3** Targeted base editing at *OsTOE1* and *OsIDS1* by ABE‐P4.
**Figure S4** Targeted base editing at the *SNB* gene by ABE‐P5.
**Figure S5** Targeted mutation at the *PMS1* and *PMS3* genes by pSaKKH‐Cas9 and pVQR‐Cas9.
**Table S1** Primers for sgRNAs used in this study.
**Table S2** Primers for target sites amplification and sequencing.

## References

[pbi12993-bib-0001] Fan, Y. and Zhang, Q. (2018) Genetic and molecular characterization of photoperiod and thermo‐sensitive male sterility in rice. Plant Reprod. 31, 3–14. 29094211 10.1007/s00497-017-0310-5

[pbi12993-bib-0002] Gaudelli, N.M. , Komor, A.C. , Rees, H.A. , Packer, M.S. , Badran, A.H. , Bryson, D.I. and Liu, D.R. (2017) Programmable base editing of A·T to G·C in genomic DNA without DNA cleavage. Nature, 551, 464–471. 29160308 10.1038/nature24644 PMC5726555

[pbi12993-bib-0003] Hua, K. , Tao, X. , Yuan, F. , Wang, D. and Zhu, J.K. (2018) Precise A·T to G·C base editing in the rice genome. Mol. Plant, 11, 627–630. 29476916 10.1016/j.molp.2018.02.007

[pbi12993-bib-0004] Kim, Y.B. , Komor, A.C. , Levy, J.M. , Packer, M.S. , Zhao, K.T. and Liu, D.R. (2017) Increasing the genome‐targeting scope and precision of base editing with engineered Cas9‐cytidine deaminase fusions. Nat. Biotechnol. 35, 371–376. 28191901 10.1038/nbt.3803 PMC5388574

[pbi12993-bib-0005] Kleinstiver, B.P. , Prew, M.S. , Tsai, S.Q. , Nguyen, N.T. , Topkar, V.V. , Zheng, Z. and Joung, J.K. (2015a) Broadening the targeting range of *Staphylococcus aureus* CRISPR‐Cas9 by modifying PAM recognition. Nat. Biotechnol. 33, 1293–1298. 26524662 10.1038/nbt.3404 PMC4689141

[pbi12993-bib-0006] Kleinstiver, B.P. , Prew, M.S. , Tsai, S.Q. , Topkar, V.V. , Nguyen, N.T. , Zheng, Z. , Gonzales, A.P.W. *et al* (2015b) Engineered CRISPR‐Cas9 nucleases with altered PAM specificities. Nature, 523, 481–485. 26098369 10.1038/nature14592 PMC4540238

[pbi12993-bib-0007] Komor, A.C. , Kim, Y.B. , Packer, M.S. , Zuris, J.A. and Liu, D.R. (2016) Programmable editing of a target base in genomic DNA without double‐stranded DNA cleavage. Nature, 533, 420–424. 27096365 10.1038/nature17946 PMC4873371

[pbi12993-bib-0008] Li, J. , Sun, Y. , Du, J. , Zhao, Y. and Xia, L. (2017) Generation of targeted point mutations in rice by a modified CRISPR/Cas9 system. Mol. Plant, 10, 526–529. 27940306 10.1016/j.molp.2016.12.001

[pbi12993-bib-0009] Liu, Z. , Chen, M. , Chen, S. , Deng, J. , Song, Y. , Lai, L. and Li, Z. (2018a) Highly efficient RNA‐guided base editing in rabbit. Nat. Commun. 9, 2717. 30006570 10.1038/s41467-018-05232-2 PMC6045575

[pbi12993-bib-0010] Liu, Z. , Lu, Z. , Yang, G. , Huang, S. , Li, G. , Feng, S. , Liu, Y. *et al* (2018b) Efficient generation of mouse models of human diseases via ABE‐ and BE‐mediated base editing. Nature Commun. 9, 2338. 29904106 10.1038/s41467-018-04768-7 PMC6002399

[pbi12993-bib-0011] Lu, Y. and Zhu, J.K. (2017) Precise editing of a target base in the rice genome using a modified CRISPR/Cas9 system. Mol. Plant, 10, 523–525. 27932049 10.1016/j.molp.2016.11.013

[pbi12993-bib-0012] Meng, X. , Hu, X. , Liu, Q. , Song, X. , Gao, C. , Li, J. and Wang, K. (2018) Robust genome editing of CRISPR‐Cas9 at NAG PAMs in rice. Sci. China Life Sci. 61, 122–125. 29285711 10.1007/s11427-017-9247-9

[pbi12993-bib-0013] Michael, P. and Holger, P. (2017) From classical mutagenesis to nuclease‐based breeding – directing natural DNA repair for a natural end‐product. Plant J. 90, 819–833. 28027431 10.1111/tpj.13469

[pbi12993-bib-0014] Nishida, K. , Arazoe, T. , Yachie, N. , Banno, S. , Kakimoto, M. , Tabata, M. , Mochizuki, M. *et al* (2016) Targeted nucleotide editing using hybrid prokaryotic and vertebrate adaptive immune systems. Science, 353, aaf8729. 27492474 10.1126/science.aaf8729

[pbi12993-bib-0015] Nishimura, A. , Aichi, I. and Matsuoka, M. (2007) A protocol for Agrobacterium‐mediated transformation in rice. Nat. Protoc. 1, 2796–2802. 10.1038/nprot.2006.469 17406537

[pbi12993-bib-0016] Shan, Q. , Wang, Y. , Li, J. and Gao, C. (2014) Genome editing in rice and wheat using the CRISPR/Cas system. Nat. Protoc. 9, 2395–2410. 25232936 10.1038/nprot.2014.157

[pbi12993-bib-0017] Yan, F. , Kuang, Y. , Ren, B. , Wang, J. , Zhang, D. , Lin, H. , Yang, B. *et al* (2018) High‐efficient A·T to G·C base editing by Cas9n‐guided tRNA adenosine deaminase in rice. Mol. Plant, 11, 631–634. 29476918 10.1016/j.molp.2018.02.008

