# Supplementary material for: Expanding the base editing scope in rice by using Cas9 variants
Source: Plant Biotechnol J. 2018 Oct 5;17(2):499–504. doi: 10.1111/pbi.12993 (PMC6335069; doi:10.1111/pbi.12993)
Supplement: Supplementary file 1 — Data S1 Complete DNA sequences of all vectors used in this study. Figure S1 Base editing by ABE‐P1 at the genomic sequences with NAG PAMs. Figure S2 Targeted base editing at OsSPL16 and OsSPL18 by ABE‐P3. Figure S3 Targeted base editing at OsTOE1 and OsIDS1 by ABE‐P4. Figure S4 Targeted base editing at the SNB gene by ABE‐P5. Figure S5 Targeted mutation at the PMS1 and PMS3 genes by pSaKKH‐Cas9 and pVQR‐Cas9. Table S1 Primers for sgRNAs used in this study. Table S2 Primers for target sites amplification and sequencing. [file PBI-17-499-s001.docx]

**Supporting Information**

**Expanding the base editing scope in rice by using Cas9 variants**

Kai Hua, Xiaoping Tao, Jian-Kang Zhu

**Contents**

**Supplemental sequence:** Complete DNA sequences of all vectors used in this study.

**Figure S1.** Base editing by ABE-P1 at the genomic sequences with NAG PAMs.

**Figure S2.** Targeted base editing at *OsSPL16* and *OsSPL18* by ABE-P3.

**Figure S3.** Targeted base editing at *OsTOE1* and *OsIDS1* by ABE-P4.

**Figure S4.** Targeted base editing at the *SNB* gene by ABE-P5.

**Figure S5.** Targeted mutation at the *PMS1* and *PMS3* genes by pSaKKH-Cas9 and pVQR-Cas9.

**Table S1.** Primers for sgRNAs used in this study.

**Table S2.** Primers for target sites amplification and sequencing.

**Supplemental sequence:** Complete DNA sequences of all vectors used in this study.

Note: The OsU6 promoter and the sgRNA scaffold for SpCas9 or SaCas9 are marked in blue and red, respectively. The maize ubiquitin promoter is highlighted in green. The mammalian codon-optimized ABE7-10 is marked in orange. The mammalian codon-optimized rAPOBEC1 with a XTEN linker and the UGI in cytosine base editors are highlighted in brown and pink, respectively. The NLSs are shown in purple, whereas the NOS terminator is highlighted in yellow. Two BsaI sites for sgRNA cloning are written in lower cases. The wild type and the D10A nickase form of human codon-optimized SpCas9, SaCas9 and their variants are written in bold. Specific mutation sites introduced into the SpCas9 and SaCas9 variants are underlined.

ABE-P1

GGATCATGAACCAACGGCCTGGCTGTATTTGGTGGTTGTGTAGGGAGATGGGGAGAAGAAAAGCCCGATTCTCTTCGCTGTGATGGGCTGGATGCATGCGGGGGAGCGGGAGGCCCAAGTACGTGCACGGTGAGCGGCCCACAGGGCGAGTGTGAGCGCGAGAGGCGGGAGGAACAGTTTAGTACCACATTGCCCAGCTAACTCGAACGCGACCAACTTATAAACCCGCGCGCTGTCGCTTGTGTGAgagaccATCTAGACggtctcTGTTTCAGAGCTATGCTGGAAACAGCATAGCAAGTTGAAATAAGGCTAGTCCGTTATCAACTTGAAAAAGTGGCACCGAGTCGGTGCTTTTTTTGTTTTTTGGCCGGCATGGTCCCAGCCTCCTCGCTGGCGCCGGCTGGGCAACATGCTTCGGCATGGCGAATGGGACTTTTTTTGTTTTTTCCCGGGTGCAGCGTGACCCGGTCGTGCCCCTCTCTAGAGATAATGAGCATTGCATGTCTAAGTTATAAAAAATTACCACATATTTTTTTTGTCACACTTGTTTGAAGTGCAGTTTATCTATCTTTATACATATATTTAAACTTTACTCTACGAATAATATAATCTATAGTACTACAATAATATCAGTGTTTTAGAGAATCATATAAATGAACAGTTAGACATGGTCTAAAGGACAATTGAGTATTTTGACAACAGGACTCTACAGTTTTATCTTTTTAGTGTGCATGTGTTCTCCTTTTTTTTTGCAAATAGCTTCACCTATATAATACTTCATCCATTTTATTAGTACATCCATTTAGGGTTTAGGGTTAATGGTTTTTATAGACTAATTTTTTTAGTACATCTATTTTATTCTATTTTAGCCTCTAAATTAAGAAAACTAAAACTCTATTTTAGTTTTTTTATTTAATAATTTAGATATAAAATAGAATAAAATAAAGTGACTAAAAATTAAACAAATACCCTTTAAGAAATTAAAAAAACTAAGGAAACATTTTTCTTGTTTCGAGTAGATAATGCCAGCCTGTTAAACGCCGTCGACGAGTCTAACGGACACCAACCAGCGAACCAGCAGCGTCGCGTCGGGCCAAGCGAAGCAGACGGCACGGCATCTCTGTCGCTGCCTCTGGACCCCTATCGAGAGTTCCGCTCCACCGTTGGACTTGCTCCGCTGTCGGCATCCAGAAATTGCGTGGCGGAGCGGCAGACGTGAGCCGGCACGGCAGGCGGCCTCCTCCTCCTCTCACGGCACGGCAGCTACGGGGGATTCCTTTCCCACCGCTCCTTCGCTTTCCCTTCCTCGCCCGCCGTAATAAATAGACACCCCCTCCACACCCTCTTTCCCCAACCTCGTGTTGTTCGGAGCGCACACACACACAACCAGATCTCCCCCAAATCCACCCGTCGGCACCTCCGCTTCAAGGTACGCCGCTCGTCCTCCCCCCCCCCCCCTCTCTACCTTCTCTAGATCGGCGTTCCGGTCCATGGTTAGGGCCCGGTAGTTCTACTTCTGTTCATGTTTGTGTTAGATCCGTGTTTGTGTTAGATCCGTGCTGCTAGCGTTCGTACACGGATGCGACCTGTACGTCAGACACGTTCTGATTGCTAACTTGCCAGTGTTTCTCTTTGGGGAATCCTGGGATGGCTCTAGCCGTTCCGCAGACGGGATCGATTTCATGATTTTTTTTGTTTCGTTGCATAGGGTTTGGTTTGCCCTTTTCCTTTATTTCAATATATGCCGTGCACTTGTTTGTCGGGTCATCTTTTCATGCTTTTTTTTGTCTTGGTTGTGATGATGTGGTCTGGTTGGGCGGTCGTTCTAGATCGGAGTAGAATTCTGTTTCAAACTACCTGGTGGATTTATTAATTTTGGATCTGTATGTGTGTGCCATACATATTCATAGTTACGAATTGAAGATGATGGATGGAAATATCGATCTAGGATAGGTATACATGTTGATGCGGGTTTTACTGATGCATATACAGAGATGCTTTTTGTTCGCTTGGTTGTGATGATGTGGTGTGGTTGGGCGGTCGTTCATTCGTTCTAGATCGGAGTAGAATACTGTTTCAAACTACCTGGTGTATTTATTAATTTTGGAACTGTATGTGTGTGTCATACATCTTCATAGTTACGAGTTTAAGATGGATGGAAATATCGATCTAGGATAGGTATACATGTTGATGTGGGTTTTACTGATGCATATACATGATGGCATATGCAGCATCTATTCATATGCTCTAACCTTGAGTACCTATCTATTATAATAAACAAGTATGTTTTATAATTATTTTGATCTTGATATACTTGGATGATGGCATATGCAGCAGCTATATGTGGATTTTTTTAGCCCTGCCTTCATACGCTATTTATTTGCTTGGTACTGTTTCTTTTGTCGATGCTCACCCTGTTGTTTGGTGTTACTTCTGCAGCCATGTCCGAAGTCGAGTTTTCCCATGAGTACTGGATGAGACACGCATTGACTCTCGCAAAGAGGGCTTGGGATGAACGCGAGGTGCCCGTGGGGGCAGTACTCGTGCATAACAATCGCGTAATCGGCGAAGGTTGGAATAGGCCGATCGGACGCCACGACCCCACTGCACATGCGGAAATCATGGCCCTTCGACAGGGAGGGCTTGTGATGCAGAATTATCGACTTATCGATGCGACGCTGTACGTCACGCTTGAACCTTGCGTAATGTGCGCGGGAGCTATGATTCACTCCCGCATTGGACGAGTTGTATTCGGTGCCCGCGACGCCAAGACGGGTGCCGCAGGTTCACTGATGGACGTGCTGCATCACCCAGGCATGAACCACCGGGTAGAAATCACAGAAGGCATATTGGCGGACGAATGTGCGGCGCTGTTGTCCGACTTTTTTCGCATGCGGAGGCAGGAGATCAAGGCCCAGAAAAAAGCACAATCCTCTACTGACTCTGGAGGGTCCTCCGGCGGATCGTCCGGCAGCGAGACGCCAGGCACCTCCGAGAGCGCTACGCCTGAATCCTCCGGGGGATCTTCAGGAGGATCATCCGAAGTCGAGTTTTCCCATGAGTACTGGATGAGACACGCATTGACTCTCGCAAAGAGGGCTCGGGATGAACGCGAGGTGCCCGTGGGGGCAGTACTCGTGCTTAACAATCGCGTAATCGGCGAAGGTTGGAATAGGGCGATCGGACTCCACGACCCCACTGCACATGCGGAAATCATGGCCCTTCGACAGGGAGGGCTTGTGATGCAGAATTATCGACTTATCGATGCGACGCTGTACGTCACGTTTGAACCTTGCGTAATGTGCGCGGGAGCTATGATTCACTCCCGCATTGGACGAGTTGTATTCGGTGTCCGCAACGCCAAGACGGGTGCCGCAGGTTCACTGATGGACGTGCTGCATTACCCAGGCATGAACCACCGGGTAGAAATCACAGAAGGCATATTGGCGGACGAATGTGCGGCGCTGTTGTGCTACTTTTTTCGCATGCCGAGGCAGGTGTTCAATGCCCAGAAAAAAGCACAATCCTCTACTGACTCTGGAGGGTCCTCCGGCGGATCGTCCGGCAGCGAGACGCCAGGCACCTCCGAGAGCGCTACGCCTGAATCCTCCGGGGGATCTTCAGGAGGATCAGGATCC**GACAAGAAGTACAGCATCGGCCTGGCCATCGGCACCAACTCTGTGGGCTGGGCCGTGATCACCGACGAGTACAAGGTGCCCAGCAAGAAATTCAAGGTGCTGGGCAACACCGACCGGCACAGCATCAAGAAGAACCTGATCGGAGCCCTGCTGTTCGACAGCGGCGAAACAGCCGAGGCCACCCGGCTGAAGAGAACCGCCAGAAGAAGATACACCAGACGGAAGAACCGGATCTGCTATCTGCAAGAGATCTTCAGCAACGAGATGGCCAAGGTGGACGACAGCTTCTTCCACAGACTGGAAGAGTCCTTCCTGGTGGAAGAGGATAAGAAGCACGAGCGGCACCCCATCTTCGGCAACATCGTGGACGAGGTGGCCTACCACGAGAAGTACCCCACCATCTACCACCTGAGAAAGAAACTGGTGGACAGCACCGACAAGGCCGACCTGCGGCTGATCTATCTGGCCCTGGCCCACATGATCAAGTTCCGGGGCCACTTCCTGATCGAGGGCGACCTGAACCCCGACAACAGCGACGTGGACAAGCTGTTCATCCAGCTGGTGCAGACCTACAACCAGCTGTTCGAGGAAAACCCCATCAACGCCAGCGGCGTGGACGCCAAGGCCATCCTGTCTGCCAGACTGAGCAAGAGCAGACGGCTGGAAAATCTGATCGCCCAGCTGCCCGGCGAGAAGAAGAATGGCCTGTTCGGAAACCTGATTGCCCTGAGCCTGGGCCTGACCCCCAACTTCAAGAGCAACTTCGACCTGGCCGAGGATGCCAAACTGCAGCTGAGCAAGGACACCTACGACGACGACCTGGACAACCTGCTGGCCCAGATCGGCGACCAGTACGCCGACCTGTTTCTGGCCGCCAAGAACCTGTCCGACGCCATCCTGCTGAGCGACATCCTGAGAGTGAACACCGAGATCACCAAGGCCCCCCTGAGCGCCTCTATGATCAAGAGATACGACGAGCACCACCAGGACCTGACCCTGCTGAAAGCTCTCGTGCGGCAGCAGCTGCCTGAGAAGTACAAAGAGATTTTCTTCGACCAGAGCAAGAACGGCTACGCCGGCTACATTGACGGCGGAGCCAGCCAGGAAGAGTTCTACAAGTTCATCAAGCCCATCCTGGAAAAGATGGACGGCACCGAGGAACTGCTCGTGAAGCTGAACAGAGAGGACCTGCTGCGGAAGCAGCGGACCTTCGACAACGGCAGCATCCCCCACCAGATCCACCTGGGAGAGCTGCACGCCATTCTGCGGCGGCAGGAAGATTTTTACCCATTCCTGAAGGACAACCGGGAAAAGATCGAGAAGATCCTGACCTTCCGCATCCCCTACTACGTGGGCCCTCTGGCCAGGGGAAACAGCAGATTCGCCTGGATGACCAGAAAGAGCGAGGAAACCATCACCCCCTGGAACTTCGAGGAAGTGGTGGACAAGGGCGCTTCCGCCCAGAGCTTCATCGAGCGGATGACCAACTTCGATAAGAACCTGCCCAACGAGAAGGTGCTGCCCAAGCACAGCCTGCTGTACGAGTACTTCACCGTGTATAACGAGCTGACCAAAGTGAAATACGTGACCGAGGGAATGAGAAAGCCCGCCTTCCTGAGCGGCGAGCAGAAAAAGGCCATCGTGGACCTGCTGTTCAAGACCAACCGGAAAGTGACCGTGAAGCAGCTGAAAGAGGACTACTTCAAGAAAATCGAGTGCTTCGACTCCGTGGAAATCTCCGGCGTGGAAGATCGGTTCAACGCCTCCCTGGGCACATACCACGATCTGCTGAAAATTATCAAGGACAAGGACTTCCTGGACAATGAGGAAAACGAGGACATTCTGGAAGATATCGTGCTGACCCTGACACTGTTTGAGGACAGAGAGATGATCGAGGAACGGCTGAAAACCTATGCCCACCTGTTCGACGACAAAGTGATGAAGCAGCTGAAGCGGCGGAGATACACCGGCTGGGGCAGGCTGAGCCGGAAGCTGATCAACGGCATCCGGGACAAGCAGTCCGGCAAGACAATCCTGGATTTCCTGAAGTCCGACGGCTTCGCCAACAGAAACTTCATGCAGCTGATCCACGACGACAGCCTGACCTTTAAAGAGGACATCCAGAAAGCCCAGGTGTCCGGCCAGGGCGATAGCCTGCACGAGCACATTGCCAATCTGGCCGGCAGCCCCGCCATTAAGAAGGGCATCCTGCAGACAGTGAAGGTGGTGGACGAGCTTGTGAAAGTGATGGGCCGGCACAAGCCCGAGAACATCGTGATCGAAATGGCCAGAGAGAACCAGACCACCCAGAAGGGACAGAAGAACAGCCGCGAGAGAATGAAGCGGATCGAAGAGGGCATCAAAGAGCTGGGCAGCCAGATCCTGAAAGAACACCCCGTGGAAAACACCCAGCTGCAGAACGAGAAGCTGTACCTGTACTACCTGCAGAATGGGCGGGATATGTACGTGGACCAGGAACTGGACATCAACCGGCTGTCCGACTACGATGTGGACCATATCGTGCCTCAGAGCTTTCTGAAGGACGACTCCATCGACAACAAGGTGCTGACCAGAAGCGACAAGAACCGGGGCAAGAGCGACAACGTGCCCTCCGAAGAGGTCGTGAAGAAGATGAAGAACTACTGGCGGCAGCTGCTGAACGCCAAGCTGATTACCCAGAGAAAGTTCGACAATCTGACCAAGGCCGAGAGAGGCGGCCTGAGCGAACTGGATAAGGCCGGCTTCATCAAGAGACAGCTGGTGGAAACCCGGCAGATCACAAAGCACGTGGCACAGATCCTGGACTCCCGGATGAACACTAAGTACGACGAGAATGACAAGCTGATCCGGGAAGTGAAAGTGATCACCCTGAAGTCCAAGCTGGTGTCCGATTTCCGGAAGGATTTCCAGTTTTACAAAGTGCGCGAGATCAACAACTACCACCACGCCCACGACGCCTACCTGAACGCCGTCGTGGGAACCGCCCTGATCAAAAAGTACCCTAAGCTGGAAAGCGAGTTCGTGTACGGCGACTACAAGGTGTACGACGTGCGGAAGATGATCGCCAAGAGCGAGCAGGAAATCGGCAAGGCTACCGCCAAGTACTTCTTCTACAGCAACATCATGAACTTTTTCAAGACCGAGATTACCCTGGCCAACGGCGAGATCCGGAAGCGGCCTCTGATCGAGACAAACGGCGAAACCGGGGAGATCGTGTGGGATAAGGGCCGGGATTTTGCCACCGTGCGGAAAGTGCTGAGCATGCCCCAAGTGAATATCGTGAAAAAGACCGAGGTGCAGACAGGCGGCTTCAGCAAAGAGTCTATCCTGCCCAAGAGGAACAGCGATAAGCTGATCGCCAGAAAGAAGGACTGGGACCCTAAGAAGTACGGCGGCTTCGACAGCCCCACCGTGGCCTATTCTGTGCTGGTGGTGGCCAAAGTGGAAAAGGGCAAGTCCAAGAAACTGAAGAGTGTGAAAGAGCTGCTGGGGATCACCATCATGGAAAGAAGCAGCTTCGAGAAGAATCCCATCGACTTTCTGGAAGCCAAGGGCTACAAAGAAGTGAAAAAGGACCTGATCATCAAGCTGCCTAAGTACTCCCTGTTCGAGCTGGAAAACGGCCGGAAGAGAATGCTGGCCTCTGCCGGCGAACTGCAGAAGGGAAACGAACTGGCCCTGCCCTCCAAATATGTGAACTTCCTGTACCTGGCCAGCCACTATGAGAAGCTGAAGGGCTCCCCCGAGGATAATGAGCAGAAACAGCTGTTTGTGGAACAGCACAAGCACTACCTGGACGAGATCATCGAGCAGATCAGCGAGTTCTCCAAGAGAGTGATCCTGGCCGACGCTAATCTGGACAAAGTGCTGTCCGCCTACAACAAGCACCGGGATAAGCCCATCAGAGAGCAGGCCGAGAATATCATCCACCTGTTTACCCTGACCAATCTGGGAGCCCCTGCCGCCTTCAAGTACTTTGACACCACCATCGACCGGAAGAGGTACACCAGCACCAAAGAGGTGCTGGACGCCACCCTGATCCACCAGAGCATCACCGGCCTGTACGAGACACGGATCGACCTGTCTCAGCTGGGAGGCGAC**ACTAGTTCCGGCGGCAGCGCTTCTCCAAAGCGTCCGCGTGACCGTCACGATGGAGAATTGGGTGGACGCAAACGTGCAAGAGGTTAAGGTACCTGATTGATCGATAGAGCTCGAATTTCCCCGATCGTTCAAACATTTGGCAATAAAGTTTCTTAAGATTGAATCCTGTTGCCGGTCTTGCGATGATTATCATATAATTTCTGTTGAATTACGTTAAGCATGTAATAATTAACATGTAATGCATGACGTTATTTATGAGATGGGTTTTTATGATTAGAGTCCCGCAATTATACATTTAATACGCGATAGAAAACAAAATATAGCGCGCAAACTAGGATAAATTATCGCGCGCGGTGTCATCTATGTTACTAGATCGG

ABE-P2

GGATCATGAACCAACGGCCTGGCTGTATTTGGTGGTTGTGTAGGGAGATGGGGAGAAGAAAAGCCCGATTCTCTTCGCTGTGATGGGCTGGATGCATGCGGGGGAGCGGGAGGCCCAAGTACGTGCACGGTGAGCGGCCCACAGGGCGAGTGTGAGCGCGAGAGGCGGGAGGAACAGTTTAGTACCACATTGCCCAGCTAACTCGAACGCGACCAACTTATAAACCCGCGCGCTGTCGCTTGTGTgagaccATCTAGACggtctcTGTTTTAGTACTCTGGAAACAGAATCTACTAAAACAAGGCAAAATGCCGTGTTTATCTCGTCAACTTGTTGGCGAGATTTTTTTTTGTTTTTTATGTCTCCCGGGTGCAGCGTGACCCGGTCGTGCCCCTCTCTAGAGATAATGAGCATTGCATGTCTAAGTTATAAAAAATTACCACATATTTTTTTTGTCACACTTGTTTGAAGTGCAGTTTATCTATCTTTATACATATATTTAAACTTTACTCTACGAATAATATAATCTATAGTACTACAATAATATCAGTGTTTTAGAGAATCATATAAATGAACAGTTAGACATGGTCTAAAGGACAATTGAGTATTTTGACAACAGGACTCTACAGTTTTATCTTTTTAGTGTGCATGTGTTCTCCTTTTTTTTTGCAAATAGCTTCACCTATATAATACTTCATCCATTTTATTAGTACATCCATTTAGGGTTTAGGGTTAATGGTTTTTATAGACTAATTTTTTTAGTACATCTATTTTATTCTATTTTAGCCTCTAAATTAAGAAAACTAAAACTCTATTTTAGTTTTTTTATTTAATAATTTAGATATAAAATAGAATAAAATAAAGTGACTAAAAATTAAACAAATACCCTTTAAGAAATTAAAAAAACTAAGGAAACATTTTTCTTGTTTCGAGTAGATAATGCCAGCCTGTTAAACGCCGTCGACGAGTCTAACGGACACCAACCAGCGAACCAGCAGCGTCGCGTCGGGCCAAGCGAAGCAGACGGCACGGCATCTCTGTCGCTGCCTCTGGACCCCTATCGAGAGTTCCGCTCCACCGTTGGACTTGCTCCGCTGTCGGCATCCAGAAATTGCGTGGCGGAGCGGCAGACGTGAGCCGGCACGGCAGGCGGCCTCCTCCTCCTCTCACGGCACGGCAGCTACGGGGGATTCCTTTCCCACCGCTCCTTCGCTTTCCCTTCCTCGCCCGCCGTAATAAATAGACACCCCCTCCACACCCTCTTTCCCCAACCTCGTGTTGTTCGGAGCGCACACACACACAACCAGATCTCCCCCAAATCCACCCGTCGGCACCTCCGCTTCAAGGTACGCCGCTCGTCCTCCCCCCCCCCCCCTCTCTACCTTCTCTAGATCGGCGTTCCGGTCCATGGTTAGGGCCCGGTAGTTCTACTTCTGTTCATGTTTGTGTTAGATCCGTGTTTGTGTTAGATCCGTGCTGCTAGCGTTCGTACACGGATGCGACCTGTACGTCAGACACGTTCTGATTGCTAACTTGCCAGTGTTTCTCTTTGGGGAATCCTGGGATGGCTCTAGCCGTTCCGCAGACGGGATCGATTTCATGATTTTTTTTGTTTCGTTGCATAGGGTTTGGTTTGCCCTTTTCCTTTATTTCAATATATGCCGTGCACTTGTTTGTCGGGTCATCTTTTCATGCTTTTTTTTGTCTTGGTTGTGATGATGTGGTCTGGTTGGGCGGTCGTTCTAGATCGGAGTAGAATTCTGTTTCAAACTACCTGGTGGATTTATTAATTTTGGATCTGTATGTGTGTGCCATACATATTCATAGTTACGAATTGAAGATGATGGATGGAAATATCGATCTAGGATAGGTATACATGTTGATGCGGGTTTTACTGATGCATATACAGAGATGCTTTTTGTTCGCTTGGTTGTGATGATGTGGTGTGGTTGGGCGGTCGTTCATTCGTTCTAGATCGGAGTAGAATACTGTTTCAAACTACCTGGTGTATTTATTAATTTTGGAACTGTATGTGTGTGTCATACATCTTCATAGTTACGAGTTTAAGATGGATGGAAATATCGATCTAGGATAGGTATACATGTTGATGTGGGTTTTACTGATGCATATACATGATGGCATATGCAGCATCTATTCATATGCTCTAACCTTGAGTACCTATCTATTATAATAAACAAGTATGTTTTATAATTATTTTGATCTTGATATACTTGGATGATGGCATATGCAGCAGCTATATGTGGATTTTTTTAGCCCTGCCTTCATACGCTATTTATTTGCTTGGTACTGTTTCTTTTGTCGATGCTCACCCTGTTGTTTGGTGTTACTTCTGCAGCCATGTCCGAAGTCGAGTTTTCCCATGAGTACTGGATGAGACACGCATTGACTCTCGCAAAGAGGGCTTGGGATGAACGCGAGGTGCCCGTGGGGGCAGTACTCGTGCATAACAATCGCGTAATCGGCGAAGGTTGGAATAGGCCGATCGGACGCCACGACCCCACTGCACATGCGGAAATCATGGCCCTTCGACAGGGAGGGCTTGTGATGCAGAATTATCGACTTATCGATGCGACGCTGTACGTCACGCTTGAACCTTGCGTAATGTGCGCGGGAGCTATGATTCACTCCCGCATTGGACGAGTTGTATTCGGTGCCCGCGACGCCAAGACGGGTGCCGCAGGTTCACTGATGGACGTGCTGCATCACCCAGGCATGAACCACCGGGTAGAAATCACAGAAGGCATATTGGCGGACGAATGTGCGGCGCTGTTGTCCGACTTTTTTCGCATGCGGAGGCAGGAGATCAAGGCCCAGAAAAAAGCACAATCCTCTACTGACTCTGGAGGGTCCTCCGGCGGATCGTCCGGCAGCGAGACGCCAGGCACCTCCGAGAGCGCTACGCCTGAATCCTCCGGGGGATCTTCAGGAGGATCATCCGAAGTCGAGTTTTCCCATGAGTACTGGATGAGACACGCATTGACTCTCGCAAAGAGGGCTCGGGATGAACGCGAGGTGCCCGTGGGGGCAGTACTCGTGCTTAACAATCGCGTAATCGGCGAAGGTTGGAATAGGGCGATCGGACTCCACGACCCCACTGCACATGCGGAAATCATGGCCCTTCGACAGGGAGGGCTTGTGATGCAGAATTATCGACTTATCGATGCGACGCTGTACGTCACGTTTGAACCTTGCGTAATGTGCGCGGGAGCTATGATTCACTCCCGCATTGGACGAGTTGTATTCGGTGTCCGCAACGCCAAGACGGGTGCCGCAGGTTCACTGATGGACGTGCTGCATTACCCAGGCATGAACCACCGGGTAGAAATCACAGAAGGCATATTGGCGGACGAATGTGCGGCGCTGTTGTGCTACTTTTTTCGCATGCCGAGGCAGGTGTTCAATGCCCAGAAAAAAGCACAATCCTCTACTGACTCTGGAGGGTCCTCCGGCGGATCGTCCGGCAGCGAGACGCCAGGCACCTCCGAGAGCGCTACGCCTGAATCCTCCGGGGGATCTTCAGGAGGATCAGGATCC**AAGCGGAACTACATCCTGGGCCTGGCCATCGGCATCACCAGCGTGGGCTACGGCATCATCGACTACGAGACACGGGACGTGATCGATGCCGGCGTGCGGCTGTTCAAAGAGGCCAACGTGGAAAACAACGAGGGCAGGCGGAGCAAGAGAGGCGCCAGAAGGCTGAAGCGGCGGAGGCGGCATAGAATCCAGAGAGTGAAGAAGCTGCTGTTCGACTACAACCTGCTGACCGACCACAGCGAGCTGAGCGGCATCAACCCCTACGAGGCCAGAGTGAAGGGCCTGAGCCAGAAGCTGAGCGAGGAAGAGTTCTCTGCCGCCCTGCTGCACCTGGCCAAGAGAAGAGGCGTGCACAACGTGAACGAGGTGGAAGAGGACACCGGCAACGAGCTGTCCACCAAAGAGCAGATCAGCCGGAACAGCAAGGCCCTGGAAGAGAAATACGTGGCCGAACTGCAGCTGGAACGGCTGAAGAAAGACGGCGAAGTGCGGGGCAGCATCAACAGATTCAAGACCAGCGACTACGTGAAAGAAGCCAAACAGCTGCTGAAGGTGCAGAAGGCCTACCACCAGCTGGACCAGAGCTTCATCGACACCTACATCGACCTGCTGGAAACCCGGCGGACCTACTATGAGGGACCTGGCGAGGGCAGCCCCTTCGGCTGGAAGGACATCAAAGAATGGTACGAGATGCTGATGGGCCACTGCACCTACTTCCCCGAGGAACTGCGGAGCGTGAAGTACGCCTACAACGCCGACCTGTACAACGCCCTGAACGACCTGAACAATCTCGTGATCACCAGGGACGAGAACGAGAAGCTGGAATATTACGAGAAGTTCCAGATCATCGAGAACGTGTTCAAGCAGAAGAAGAAGCCCACCCTGAAGCAGATCGCCAAAGAAATCCTCGTGAACGAAGAGGATATTAAGGGCTACAGAGTGACCAGCACCGGCAAGCCCGAGTTCACCAACCTGAAGGTGTACCACGACATCAAGGACATTACCGCCCGGAAAGAGATTATTGAGAACGCCGAGCTGCTGGATCAGATTGCCAAGATCCTGACCATCTACCAGAGCAGCGAGGACATCCAGGAAGAACTGACCAATCTGAACTCCGAGCTGACCCAGGAAGAGATCGAGCAGATCTCTAATCTGAAGGGCTATACCGGCACCCACAACCTGAGCCTGAAGGCCATCAACCTGATCCTGGACGAGCTGTGGCACACCAACGACAACCAGATCGCTATCTTCAACCGGCTGAAGCTGGTGCCCAAGAAGGTGGACCTGTCCCAGCAGAAAGAGATCCCCACCACCCTGGTGGACGACTTCATCCTGAGCCCCGTCGTGAAGAGAAGCTTCATCCAGAGCATCAAAGTGATCAACGCCATCATCAAGAAGTACGGCCTGCCCAACGACATCATTATCGAGCTGGCCCGCGAGAAGAACTCCAAGGACGCCCAGAAAATGATCAACGAGATGCAGAAGCGGAACCGGCAGACCAACGAGCGGATCGAGGAAATCATCCGGACCACCGGCAAAGAGAACGCCAAGTACCTGATCGAGAAGATCAAGCTGCACGACATGCAGGAAGGCAAGTGCCTGTACAGCCTGGAAGCCATCCCTCTGGAAGATCTGCTGAACAACCCCTTCAACTATGAGGTGGACCACATCATCCCCAGAAGCGTGTCCTTCGACAACAGCTTCAACAACAAGGTGCTCGTGAAGCAGGAAGAAAACAGCAAGAAGGGCAACCGGACCCCATTCCAGTACCTGAGCAGCAGCGACAGCAAGATCAGCTACGAAACCTTCAAGAAGCACATCCTGAATCTGGCCAAGGGCAAGGGCAGAATCAGCAAGACCAAGAAAGAGTATCTGCTGGAAGAACGGGACATCAACAGGTTCTCCGTGCAGAAAGACTTCATCAACCGGAACCTGGTGGATACCAGATACGCCACCAGAGGCCTGATGAACCTGCTGCGGAGCTACTTCAGAGTGAACAACCTGGACGTGAAAGTGAAGTCCATCAATGGCGGCTTCACCAGCTTTCTGCGGCGGAAGTGGAAGTTTAAGAAAGAGCGGAACAAGGGGTACAAGCACCACGCCGAGGACGCCCTGATCATTGCCAACGCCGATTTCATCTTCAAAGAGTGGAAGAAACTGGACAAGGCCAAAAAAGTGATGGAAAACCAGATGTTCGAGGAAAAGCAGGCCGAGAGCATGCCCGAGATCGAAACCGAGCAGGAGTACAAAGAGATCTTCATCACCCCCCACCAGATCAAGCACATTAAGGACTTCAAGGACTACAAGTACAGCCACCGGGTGGACAAGAAGCCTAATAGAGAGCTGATTAACGACACCCTGTACTCCACCCGGAAGGACGACAAGGGCAACACCCTGATCGTGAACAATCTGAACGGCCTGTACGACAAGGACAATGACAAGCTGAAAAAGCTGATCAACAAGAGCCCCGAAAAGCTGCTGATGTACCACCACGACCCCCAGACCTACCAGAAACTGAAGCTGATTATGGAACAGTACGGCGACGAGAAGAATCCCCTGTACAAGTACTACGAGGAAACCGGGAACTACCTGACCAAGTACTCCAAAAAGGACAACGGCCCCGTGATCAAGAAGATTAAGTATTACGGCAACAAACTGAACGCCCATCTGGACATCACCGACGACTACCCCAACAGCAGAAACAAGGTCGTGAAGCTGTCCCTGAAGCCCTACAGATTCGACGTGTACCTGGACAATGGCGTGTACAAGTTCGTGACCGTGAAGAATCTGGATGTGATCAAAAAAGAAAACTACTACGAAGTGAATAGCAAGTGCTATGAGGAAGCTAAGAAGCTGAAGAAGATCAGCAACCAGGCCGAGTTTATCGCCTCCTTCTACAACAACGATCTGATCAAGATCAACGGCGAGCTGTATAGAGTGATCGGCGTGAACAACGACCTGCTGAACCGGATCGAAGTGAACATGATCGACATCACCTACCGCGAGTACCTGGAAAACATGAACGACAAGAGGCCCCCCAGGATCATTAAGACAATCGCCTCCAAGACCCAGAGCATTAAGAAGTACAGCACAGACATTCTGGGCAACCTGTATGAAGTGAAATCTAAGAAGCACCCTCAGATCATCAAAAAGGG**ACTAGTTCCGGCGGCAGCGCTTCTCCAAAGCGTCCGCGTGACCGTCACGATGGAGAATTGGGTGGACGCAAACGTGCAAGAGGTTAAGGTACCTGATTGATCGATAGAGCTCGAATTTCCCCGATCGTTCAAACATTTGGCAATAAAGTTTCTTAAGATTGAATCCTGTTGCCGGTCTTGCGATGATTATCATATAATTTCTGTTGAATTACGTTAAGCATGTAATAATTAACATGTAATGCATGACGTTATTTATGAGATGGGTTTTTATGATTAGAGTCCCGCAATTATACATTTAATACGCGATAGAAAACAAAATATAGCGCGCAAACTAGGATAAATTATCGCGCGCGGTGTCATCTATGTTACTAGATCGG

ABE-P3

GGATCATGAACCAACGGCCTGGCTGTATTTGGTGGTTGTGTAGGGAGATGGGGAGAAGAAAAGCCCGATTCTCTTCGCTGTGATGGGCTGGATGCATGCGGGGGAGCGGGAGGCCCAAGTACGTGCACGGTGAGCGGCCCACAGGGCGAGTGTGAGCGCGAGAGGCGGGAGGAACAGTTTAGTACCACATTGCCCAGCTAACTCGAACGCGACCAACTTATAAACCCGCGCGCTGTCGCTTGTGTGAgagaccATCTAGACggtctcTGTTTCAGAGCTATGCTGGAAACAGCATAGCAAGTTGAAATAAGGCTAGTCCGTTATCAACTTGAAAAAGTGGCACCGAGTCGGTGCTTTTTTTGTTTTTTGGCCGGCATGGTCCCAGCCTCCTCGCTGGCGCCGGCTGGGCAACATGCTTCGGCATGGCGAATGGGACTTTTTTTGTTTTTTCCCGGGTGCAGCGTGACCCGGTCGTGCCCCTCTCTAGAGATAATGAGCATTGCATGTCTAAGTTATAAAAAATTACCACATATTTTTTTTGTCACACTTGTTTGAAGTGCAGTTTATCTATCTTTATACATATATTTAAACTTTACTCTACGAATAATATAATCTATAGTACTACAATAATATCAGTGTTTTAGAGAATCATATAAATGAACAGTTAGACATGGTCTAAAGGACAATTGAGTATTTTGACAACAGGACTCTACAGTTTTATCTTTTTAGTGTGCATGTGTTCTCCTTTTTTTTTGCAAATAGCTTCACCTATATAATACTTCATCCATTTTATTAGTACATCCATTTAGGGTTTAGGGTTAATGGTTTTTATAGACTAATTTTTTTAGTACATCTATTTTATTCTATTTTAGCCTCTAAATTAAGAAAACTAAAACTCTATTTTAGTTTTTTTATTTAATAATTTAGATATAAAATAGAATAAAATAAAGTGACTAAAAATTAAACAAATACCCTTTAAGAAATTAAAAAAACTAAGGAAACATTTTTCTTGTTTCGAGTAGATAATGCCAGCCTGTTAAACGCCGTCGACGAGTCTAACGGACACCAACCAGCGAACCAGCAGCGTCGCGTCGGGCCAAGCGAAGCAGACGGCACGGCATCTCTGTCGCTGCCTCTGGACCCCTATCGAGAGTTCCGCTCCACCGTTGGACTTGCTCCGCTGTCGGCATCCAGAAATTGCGTGGCGGAGCGGCAGACGTGAGCCGGCACGGCAGGCGGCCTCCTCCTCCTCTCACGGCACGGCAGCTACGGGGGATTCCTTTCCCACCGCTCCTTCGCTTTCCCTTCCTCGCCCGCCGTAATAAATAGACACCCCCTCCACACCCTCTTTCCCCAACCTCGTGTTGTTCGGAGCGCACACACACACAACCAGATCTCCCCCAAATCCACCCGTCGGCACCTCCGCTTCAAGGTACGCCGCTCGTCCTCCCCCCCCCCCCCTCTCTACCTTCTCTAGATCGGCGTTCCGGTCCATGGTTAGGGCCCGGTAGTTCTACTTCTGTTCATGTTTGTGTTAGATCCGTGTTTGTGTTAGATCCGTGCTGCTAGCGTTCGTACACGGATGCGACCTGTACGTCAGACACGTTCTGATTGCTAACTTGCCAGTGTTTCTCTTTGGGGAATCCTGGGATGGCTCTAGCCGTTCCGCAGACGGGATCGATTTCATGATTTTTTTTGTTTCGTTGCATAGGGTTTGGTTTGCCCTTTTCCTTTATTTCAATATATGCCGTGCACTTGTTTGTCGGGTCATCTTTTCATGCTTTTTTTTGTCTTGGTTGTGATGATGTGGTCTGGTTGGGCGGTCGTTCTAGATCGGAGTAGAATTCTGTTTCAAACTACCTGGTGGATTTATTAATTTTGGATCTGTATGTGTGTGCCATACATATTCATAGTTACGAATTGAAGATGATGGATGGAAATATCGATCTAGGATAGGTATACATGTTGATGCGGGTTTTACTGATGCATATACAGAGATGCTTTTTGTTCGCTTGGTTGTGATGATGTGGTGTGGTTGGGCGGTCGTTCATTCGTTCTAGATCGGAGTAGAATACTGTTTCAAACTACCTGGTGTATTTATTAATTTTGGAACTGTATGTGTGTGTCATACATCTTCATAGTTACGAGTTTAAGATGGATGGAAATATCGATCTAGGATAGGTATACATGTTGATGTGGGTTTTACTGATGCATATACATGATGGCATATGCAGCATCTATTCATATGCTCTAACCTTGAGTACCTATCTATTATAATAAACAAGTATGTTTTATAATTATTTTGATCTTGATATACTTGGATGATGGCATATGCAGCAGCTATATGTGGATTTTTTTAGCCCTGCCTTCATACGCTATTTATTTGCTTGGTACTGTTTCTTTTGTCGATGCTCACCCTGTTGTTTGGTGTTACTTCTGCAGCCATGTCCGAAGTCGAGTTTTCCCATGAGTACTGGATGAGACACGCATTGACTCTCGCAAAGAGGGCTTGGGATGAACGCGAGGTGCCCGTGGGGGCAGTACTCGTGCATAACAATCGCGTAATCGGCGAAGGTTGGAATAGGCCGATCGGACGCCACGACCCCACTGCACATGCGGAAATCATGGCCCTTCGACAGGGAGGGCTTGTGATGCAGAATTATCGACTTATCGATGCGACGCTGTACGTCACGCTTGAACCTTGCGTAATGTGCGCGGGAGCTATGATTCACTCCCGCATTGGACGAGTTGTATTCGGTGCCCGCGACGCCAAGACGGGTGCCGCAGGTTCACTGATGGACGTGCTGCATCACCCAGGCATGAACCACCGGGTAGAAATCACAGAAGGCATATTGGCGGACGAATGTGCGGCGCTGTTGTCCGACTTTTTTCGCATGCGGAGGCAGGAGATCAAGGCCCAGAAAAAAGCACAATCCTCTACTGACTCTGGAGGGTCCTCCGGCGGATCGTCCGGCAGCGAGACGCCAGGCACCTCCGAGAGCGCTACGCCTGAATCCTCCGGGGGATCTTCAGGAGGATCATCCGAAGTCGAGTTTTCCCATGAGTACTGGATGAGACACGCATTGACTCTCGCAAAGAGGGCTCGGGATGAACGCGAGGTGCCCGTGGGGGCAGTACTCGTGCTTAACAATCGCGTAATCGGCGAAGGTTGGAATAGGGCGATCGGACTCCACGACCCCACTGCACATGCGGAAATCATGGCCCTTCGACAGGGAGGGCTTGTGATGCAGAATTATCGACTTATCGATGCGACGCTGTACGTCACGTTTGAACCTTGCGTAATGTGCGCGGGAGCTATGATTCACTCCCGCATTGGACGAGTTGTATTCGGTGTCCGCAACGCCAAGACGGGTGCCGCAGGTTCACTGATGGACGTGCTGCATTACCCAGGCATGAACCACCGGGTAGAAATCACAGAAGGCATATTGGCGGACGAATGTGCGGCGCTGTTGTGCTACTTTTTTCGCATGCCGAGGCAGGTGTTCAATGCCCAGAAAAAAGCACAATCCTCTACTGACTCTGGAGGGTCCTCCGGCGGATCGTCCGGCAGCGAGACGCCAGGCACCTCCGAGAGCGCTACGCCTGAATCCTCCGGGGGATCTTCAGGAGGATCAGGATCC**GACAAGAAGTACAGCATCGGCCTGGCCATCGGCACCAACTCTGTGGGCTGGGCCGTGATCACCGACGAGTACAAGGTGCCCAGCAAGAAATTCAAGGTGCTGGGCAACACCGACCGGCACAGCATCAAGAAGAACCTGATCGGAGCCCTGCTGTTCGACAGCGGCGAAACAGCCGAGGCCACCCGGCTGAAGAGAACCGCCAGAAGAAGATACACCAGACGGAAGAACCGGATCTGCTATCTGCAAGAGATCTTCAGCAACGAGATGGCCAAGGTGGACGACAGCTTCTTCCACAGACTGGAAGAGTCCTTCCTGGTGGAAGAGGATAAGAAGCACGAGCGGCACCCCATCTTCGGCAACATCGTGGACGAGGTGGCCTACCACGAGAAGTACCCCACCATCTACCACCTGAGAAAGAAACTGGTGGACAGCACCGACAAGGCCGACCTGCGGCTGATCTATCTGGCCCTGGCCCACATGATCAAGTTCCGGGGCCACTTCCTGATCGAGGGCGACCTGAACCCCGACAACAGCGACGTGGACAAGCTGTTCATCCAGCTGGTGCAGACCTACAACCAGCTGTTCGAGGAAAACCCCATCAACGCCAGCGGCGTGGACGCCAAGGCCATCCTGTCTGCCAGACTGAGCAAGAGCAGACGGCTGGAAAATCTGATCGCCCAGCTGCCCGGCGAGAAGAAGAATGGCCTGTTCGGAAACCTGATTGCCCTGAGCCTGGGCCTGACCCCCAACTTCAAGAGCAACTTCGACCTGGCCGAGGATGCCAAACTGCAGCTGAGCAAGGACACCTACGACGACGACCTGGACAACCTGCTGGCCCAGATCGGCGACCAGTACGCCGACCTGTTTCTGGCCGCCAAGAACCTGTCCGACGCCATCCTGCTGAGCGACATCCTGAGAGTGAACACCGAGATCACCAAGGCCCCCCTGAGCGCCTCTATGATCAAGAGATACGACGAGCACCACCAGGACCTGACCCTGCTGAAAGCTCTCGTGCGGCAGCAGCTGCCTGAGAAGTACAAAGAGATTTTCTTCGACCAGAGCAAGAACGGCTACGCCGGCTACATTGACGGCGGAGCCAGCCAGGAAGAGTTCTACAAGTTCATCAAGCCCATCCTGGAAAAGATGGACGGCACCGAGGAACTGCTCGTGAAGCTGAACAGAGAGGACCTGCTGCGGAAGCAGCGGACCTTCGACAACGGCAGCATCCCCCACCAGATCCACCTGGGAGAGCTGCACGCCATTCTGCGGCGGCAGGAAGATTTTTACCCATTCCTGAAGGACAACCGGGAAAAGATCGAGAAGATCCTGACCTTCCGCATCCCCTACTACGTGGGCCCTCTGGCCAGGGGAAACAGCAGATTCGCCTGGATGACCAGAAAGAGCGAGGAAACCATCACCCCCTGGAACTTCGAGGAAGTGGTGGACAAGGGCGCTTCCGCCCAGAGCTTCATCGAGCGGATGACCAACTTCGATAAGAACCTGCCCAACGAGAAGGTGCTGCCCAAGCACAGCCTGCTGTACGAGTACTTCACCGTGTATAACGAGCTGACCAAAGTGAAATACGTGACCGAGGGAATGAGAAAGCCCGCCTTCCTGAGCGGCGAGCAGAAAAAGGCCATCGTGGACCTGCTGTTCAAGACCAACCGGAAAGTGACCGTGAAGCAGCTGAAAGAGGACTACTTCAAGAAAATCGAGTGCTTCGACTCCGTGGAAATCTCCGGCGTGGAAGATCGGTTCAACGCCTCCCTGGGCACATACCACGATCTGCTGAAAATTATCAAGGACAAGGACTTCCTGGACAATGAGGAAAACGAGGACATTCTGGAAGATATCGTGCTGACCCTGACACTGTTTGAGGACAGAGAGATGATCGAGGAACGGCTGAAAACCTATGCCCACCTGTTCGACGACAAAGTGATGAAGCAGCTGAAGCGGCGGAGATACACCGGCTGGGGCAGGCTGAGCCGGAAGCTGATCAACGGCATCCGGGACAAGCAGTCCGGCAAGACAATCCTGGATTTCCTGAAGTCCGACGGCTTCGCCAACAGAAACTTCATGCAGCTGATCCACGACGACAGCCTGACCTTTAAAGAGGACATCCAGAAAGCCCAGGTGTCCGGCCAGGGCGATAGCCTGCACGAGCACATTGCCAATCTGGCCGGCAGCCCCGCCATTAAGAAGGGCATCCTGCAGACAGTGAAGGTGGTGGACGAGCTTGTGAAAGTGATGGGCCGGCACAAGCCCGAGAACATCGTGATCGAAATGGCCAGAGAGAACCAGACCACCCAGAAGGGACAGAAGAACAGCCGCGAGAGAATGAAGCGGATCGAAGAGGGCATCAAAGAGCTGGGCAGCCAGATCCTGAAAGAACACCCCGTGGAAAACACCCAGCTGCAGAACGAGAAGCTGTACCTGTACTACCTGCAGAATGGGCGGGATATGTACGTGGACCAGGAACTGGACATCAACCGGCTGTCCGACTACGATGTGGACCATATCGTGCCTCAGAGCTTTCTGAAGGACGACTCCATCGACAACAAGGTGCTGACCAGAAGCGACAAGAACCGGGGCAAGAGCGACAACGTGCCCTCCGAAGAGGTCGTGAAGAAGATGAAGAACTACTGGCGGCAGCTGCTGAACGCCAAGCTGATTACCCAGAGAAAGTTCGACAATCTGACCAAGGCCGAGAGAGGCGGCCTGAGCGAACTGGATAAGGCCGGCTTCATCAAGAGACAGCTGGTGGAAACCCGGCAGATCACAAAGCACGTGGCACAGATCCTGGACTCCCGGATGAACACTAAGTACGACGAGAATGACAAGCTGATCCGGGAAGTGAAAGTGATCACCCTGAAGTCCAAGCTGGTGTCCGATTTCCGGAAGGATTTCCAGTTTTACAAAGTGCGCGAGATCAACAACTACCACCACGCCCACGACGCCTACCTGAACGCCGTCGTGGGAACCGCCCTGATCAAAAAGTACCCTAAGCTGGAAAGCGAGTTCGTGTACGGCGACTACAAGGTGTACGACGTGCGGAAGATGATCGCCAAGAGCGAGCAGGAAATCGGCAAGGCTACCGCCAAGTACTTCTTCTACAGCAACATCATGAACTTTTTCAAGACCGAGATTACCCTGGCCAACGGCGAGATCCGGAAGCGGCCTCTGATCGAGACAAACGGCGAAACCGGGGAGATCGTGTGGGATAAGGGCCGGGATTTTGCCACCGTGCGGAAAGTGCTGAGCATGCCCCAAGTGAATATCGTGAAAAAGACCGAGGTGCAGACAGGCGGCTTCAGCAAAGAGTCTATCCTGCCCAAGAGGAACAGCGATAAGCTGATCGCCAGAAAGAAGGACTGGGACCCTAAGAAGTACGGCGGCTTCGTCAGCCCCACCGTGGCCTATTCTGTGCTGGTGGTGGCCAAAGTGGAAAAGGGCAAGTCCAAGAAACTGAAGAGTGTGAAAGAGCTGCTGGGGATCACCATCATGGAAAGAAGCAGCTTCGAGAAGAATCCCATCGACTTTCTGGAAGCCAAGGGCTACAAAGAAGTGAAAAAGGACCTGATCATCAAGCTGCCTAAGTACTCCCTGTTCGAGCTGGAAAACGGCCGGAAGAGAATGCTGGCCTCTGCCGGCGAACTGCAGAAGGGAAACGAACTGGCCCTGCCCTCCAAATATGTGAACTTCCTGTACCTGGCCAGCCACTATGAGAAGCTGAAGGGCTCCCCCGAGGATAATGAGCAGAAACAGCTGTTTGTGGAACAGCACAAGCACTACCTGGACGAGATCATCGAGCAGATCAGCGAGTTCTCCAAGAGAGTGATCCTGGCCGACGCTAATCTGGACAAAGTGCTGTCCGCCTACAACAAGCACCGGGATAAGCCCATCAGAGAGCAGGCCGAGAATATCATCCACCTGTTTACCCTGACCAATCTGGGAGCCCCTGCCGCCTTCAAGTACTTTGACACCACCATCGACCGGAAGCAGTACAGGAGCACCAAAGAGGTGCTGGACGCCACCCTGATCCACCAGAGCATCACCGGCCTGTACGAGACACGGATCGACCTGTCTCAGCTGGGAGGCGAC**ACTAGTTCCGGCGGCAGCGCTTCTCCAAAGCGTCCGCGTGACCGTCACGATGGAGAATTGGGTGGACGCAAACGTGCAAGAGGTTAAGGTACCTGATTGATCGATAGAGCTCGAATTTCCCCGATCGTTCAAACATTTGGCAATAAAGTTTCTTAAGATTGAATCCTGTTGCCGGTCTTGCGATGATTATCATATAATTTCTGTTGAATTACGTTAAGCATGTAATAATTAACATGTAATGCATGACGTTATTTATGAGATGGGTTTTTATGATTAGAGTCCCGCAATTATACATTTAATACGCGATAGAAAACAAAATATAGCGCGCAAACTAGGATAAATTATCGCGCGCGGTGTCATCTATGTTACTAGATCGG

ABE-P4

GGATCATGAACCAACGGCCTGGCTGTATTTGGTGGTTGTGTAGGGAGATGGGGAGAAGAAAAGCCCGATTCTCTTCGCTGTGATGGGCTGGATGCATGCGGGGGAGCGGGAGGCCCAAGTACGTGCACGGTGAGCGGCCCACAGGGCGAGTGTGAGCGCGAGAGGCGGGAGGAACAGTTTAGTACCACATTGCCCAGCTAACTCGAACGCGACCAACTTATAAACCCGCGCGCTGTCGCTTGTGTGAgagaccATCTAGACggtctcTGTTTCAGAGCTATGCTGGAAACAGCATAGCAAGTTGAAATAAGGCTAGTCCGTTATCAACTTGAAAAAGTGGCACCGAGTCGGTGCTTTTTTTGTTTTTTGGCCGGCATGGTCCCAGCCTCCTCGCTGGCGCCGGCTGGGCAACATGCTTCGGCATGGCGAATGGGACTTTTTTTGTTTTTTCCCGGGTGCAGCGTGACCCGGTCGTGCCCCTCTCTAGAGATAATGAGCATTGCATGTCTAAGTTATAAAAAATTACCACATATTTTTTTTGTCACACTTGTTTGAAGTGCAGTTTATCTATCTTTATACATATATTTAAACTTTACTCTACGAATAATATAATCTATAGTACTACAATAATATCAGTGTTTTAGAGAATCATATAAATGAACAGTTAGACATGGTCTAAAGGACAATTGAGTATTTTGACAACAGGACTCTACAGTTTTATCTTTTTAGTGTGCATGTGTTCTCCTTTTTTTTTGCAAATAGCTTCACCTATATAATACTTCATCCATTTTATTAGTACATCCATTTAGGGTTTAGGGTTAATGGTTTTTATAGACTAATTTTTTTAGTACATCTATTTTATTCTATTTTAGCCTCTAAATTAAGAAAACTAAAACTCTATTTTAGTTTTTTTATTTAATAATTTAGATATAAAATAGAATAAAATAAAGTGACTAAAAATTAAACAAATACCCTTTAAGAAATTAAAAAAACTAAGGAAACATTTTTCTTGTTTCGAGTAGATAATGCCAGCCTGTTAAACGCCGTCGACGAGTCTAACGGACACCAACCAGCGAACCAGCAGCGTCGCGTCGGGCCAAGCGAAGCAGACGGCACGGCATCTCTGTCGCTGCCTCTGGACCCCTATCGAGAGTTCCGCTCCACCGTTGGACTTGCTCCGCTGTCGGCATCCAGAAATTGCGTGGCGGAGCGGCAGACGTGAGCCGGCACGGCAGGCGGCCTCCTCCTCCTCTCACGGCACGGCAGCTACGGGGGATTCCTTTCCCACCGCTCCTTCGCTTTCCCTTCCTCGCCCGCCGTAATAAATAGACACCCCCTCCACACCCTCTTTCCCCAACCTCGTGTTGTTCGGAGCGCACACACACACAACCAGATCTCCCCCAAATCCACCCGTCGGCACCTCCGCTTCAAGGTACGCCGCTCGTCCTCCCCCCCCCCCCCTCTCTACCTTCTCTAGATCGGCGTTCCGGTCCATGGTTAGGGCCCGGTAGTTCTACTTCTGTTCATGTTTGTGTTAGATCCGTGTTTGTGTTAGATCCGTGCTGCTAGCGTTCGTACACGGATGCGACCTGTACGTCAGACACGTTCTGATTGCTAACTTGCCAGTGTTTCTCTTTGGGGAATCCTGGGATGGCTCTAGCCGTTCCGCAGACGGGATCGATTTCATGATTTTTTTTGTTTCGTTGCATAGGGTTTGGTTTGCCCTTTTCCTTTATTTCAATATATGCCGTGCACTTGTTTGTCGGGTCATCTTTTCATGCTTTTTTTTGTCTTGGTTGTGATGATGTGGTCTGGTTGGGCGGTCGTTCTAGATCGGAGTAGAATTCTGTTTCAAACTACCTGGTGGATTTATTAATTTTGGATCTGTATGTGTGTGCCATACATATTCATAGTTACGAATTGAAGATGATGGATGGAAATATCGATCTAGGATAGGTATACATGTTGATGCGGGTTTTACTGATGCATATACAGAGATGCTTTTTGTTCGCTTGGTTGTGATGATGTGGTGTGGTTGGGCGGTCGTTCATTCGTTCTAGATCGGAGTAGAATACTGTTTCAAACTACCTGGTGTATTTATTAATTTTGGAACTGTATGTGTGTGTCATACATCTTCATAGTTACGAGTTTAAGATGGATGGAAATATCGATCTAGGATAGGTATACATGTTGATGTGGGTTTTACTGATGCATATACATGATGGCATATGCAGCATCTATTCATATGCTCTAACCTTGAGTACCTATCTATTATAATAAACAAGTATGTTTTATAATTATTTTGATCTTGATATACTTGGATGATGGCATATGCAGCAGCTATATGTGGATTTTTTTAGCCCTGCCTTCATACGCTATTTATTTGCTTGGTACTGTTTCTTTTGTCGATGCTCACCCTGTTGTTTGGTGTTACTTCTGCAGCCATGTCCGAAGTCGAGTTTTCCCATGAGTACTGGATGAGACACGCATTGACTCTCGCAAAGAGGGCTTGGGATGAACGCGAGGTGCCCGTGGGGGCAGTACTCGTGCATAACAATCGCGTAATCGGCGAAGGTTGGAATAGGCCGATCGGACGCCACGACCCCACTGCACATGCGGAAATCATGGCCCTTCGACAGGGAGGGCTTGTGATGCAGAATTATCGACTTATCGATGCGACGCTGTACGTCACGCTTGAACCTTGCGTAATGTGCGCGGGAGCTATGATTCACTCCCGCATTGGACGAGTTGTATTCGGTGCCCGCGACGCCAAGACGGGTGCCGCAGGTTCACTGATGGACGTGCTGCATCACCCAGGCATGAACCACCGGGTAGAAATCACAGAAGGCATATTGGCGGACGAATGTGCGGCGCTGTTGTCCGACTTTTTTCGCATGCGGAGGCAGGAGATCAAGGCCCAGAAAAAAGCACAATCCTCTACTGACTCTGGAGGGTCCTCCGGCGGATCGTCCGGCAGCGAGACGCCAGGCACCTCCGAGAGCGCTACGCCTGAATCCTCCGGGGGATCTTCAGGAGGATCATCCGAAGTCGAGTTTTCCCATGAGTACTGGATGAGACACGCATTGACTCTCGCAAAGAGGGCTCGGGATGAACGCGAGGTGCCCGTGGGGGCAGTACTCGTGCTTAACAATCGCGTAATCGGCGAAGGTTGGAATAGGGCGATCGGACTCCACGACCCCACTGCACATGCGGAAATCATGGCCCTTCGACAGGGAGGGCTTGTGATGCAGAATTATCGACTTATCGATGCGACGCTGTACGTCACGTTTGAACCTTGCGTAATGTGCGCGGGAGCTATGATTCACTCCCGCATTGGACGAGTTGTATTCGGTGTCCGCAACGCCAAGACGGGTGCCGCAGGTTCACTGATGGACGTGCTGCATTACCCAGGCATGAACCACCGGGTAGAAATCACAGAAGGCATATTGGCGGACGAATGTGCGGCGCTGTTGTGCTACTTTTTTCGCATGCCGAGGCAGGTGTTCAATGCCCAGAAAAAAGCACAATCCTCTACTGACTCTGGAGGGTCCTCCGGCGGATCGTCCGGCAGCGAGACGCCAGGCACCTCCGAGAGCGCTACGCCTGAATCCTCCGGGGGATCTTCAGGAGGATCAGGATCC**GACAAGAAGTACAGCATCGGCCTGGCCATCGGCACCAACTCTGTGGGCTGGGCCGTGATCACCGACGAGTACAAGGTGCCCAGCAAGAAATTCAAGGTGCTGGGCAACACCGACCGGCACAGCATCAAGAAGAACCTGATCGGAGCCCTGCTGTTCGACAGCGGCGAAACAGCCGAGGCCACCCGGCTGAAGAGAACCGCCAGAAGAAGATACACCAGACGGAAGAACCGGATCTGCTATCTGCAAGAGATCTTCAGCAACGAGATGGCCAAGGTGGACGACAGCTTCTTCCACAGACTGGAAGAGTCCTTCCTGGTGGAAGAGGATAAGAAGCACGAGCGGCACCCCATCTTCGGCAACATCGTGGACGAGGTGGCCTACCACGAGAAGTACCCCACCATCTACCACCTGAGAAAGAAACTGGTGGACAGCACCGACAAGGCCGACCTGCGGCTGATCTATCTGGCCCTGGCCCACATGATCAAGTTCCGGGGCCACTTCCTGATCGAGGGCGACCTGAACCCCGACAACAGCGACGTGGACAAGCTGTTCATCCAGCTGGTGCAGACCTACAACCAGCTGTTCGAGGAAAACCCCATCAACGCCAGCGGCGTGGACGCCAAGGCCATCCTGTCTGCCAGACTGAGCAAGAGCAGACGGCTGGAAAATCTGATCGCCCAGCTGCCCGGCGAGAAGAAGAATGGCCTGTTCGGAAACCTGATTGCCCTGAGCCTGGGCCTGACCCCCAACTTCAAGAGCAACTTCGACCTGGCCGAGGATGCCAAACTGCAGCTGAGCAAGGACACCTACGACGACGACCTGGACAACCTGCTGGCCCAGATCGGCGACCAGTACGCCGACCTGTTTCTGGCCGCCAAGAACCTGTCCGACGCCATCCTGCTGAGCGACATCCTGAGAGTGAACACCGAGATCACCAAGGCCCCCCTGAGCGCCTCTATGATCAAGAGATACGACGAGCACCACCAGGACCTGACCCTGCTGAAAGCTCTCGTGCGGCAGCAGCTGCCTGAGAAGTACAAAGAGATTTTCTTCGACCAGAGCAAGAACGGCTACGCCGGCTACATTGACGGCGGAGCCAGCCAGGAAGAGTTCTACAAGTTCATCAAGCCCATCCTGGAAAAGATGGACGGCACCGAGGAACTGCTCGTGAAGCTGAACAGAGAGGACCTGCTGCGGAAGCAGCGGACCTTCGACAACGGCAGCATCCCCCACCAGATCCACCTGGGAGAGCTGCACGCCATTCTGCGGCGGCAGGAAGATTTTTACCCATTCCTGAAGGACAACCGGGAAAAGATCGAGAAGATCCTGACCTTCCGCATCCCCTACTACGTGGGCCCTCTGGCCAGGGGAAACAGCAGATTCGCCTGGATGACCAGAAAGAGCGAGGAAACCATCACCCCCTGGAACTTCGAGGAAGTGGTGGACAAGGGCGCTTCCGCCCAGAGCTTCATCGAGCGGATGACCAACTTCGATAAGAACCTGCCCAACGAGAAGGTGCTGCCCAAGCACAGCCTGCTGTACGAGTACTTCACCGTGTATAACGAGCTGACCAAAGTGAAATACGTGACCGAGGGAATGAGAAAGCCCGCCTTCCTGAGCGGCGAGCAGAAAAAGGCCATCGTGGACCTGCTGTTCAAGACCAACCGGAAAGTGACCGTGAAGCAGCTGAAAGAGGACTACTTCAAGAAAATCGAGTGCTTCGACTCCGTGGAAATCTCCGGCGTGGAAGATCGGTTCAACGCCTCCCTGGGCACATACCACGATCTGCTGAAAATTATCAAGGACAAGGACTTCCTGGACAATGAGGAAAACGAGGACATTCTGGAAGATATCGTGCTGACCCTGACACTGTTTGAGGACAGAGAGATGATCGAGGAACGGCTGAAAACCTATGCCCACCTGTTCGACGACAAAGTGATGAAGCAGCTGAAGCGGCGGAGATACACCGGCTGGGGCAGGCTGAGCCGGAAGCTGATCAACGGCATCCGGGACAAGCAGTCCGGCAAGACAATCCTGGATTTCCTGAAGTCCGACGGCTTCGCCAACAGAAACTTCATGCAGCTGATCCACGACGACAGCCTGACCTTTAAAGAGGACATCCAGAAAGCCCAGGTGTCCGGCCAGGGCGATAGCCTGCACGAGCACATTGCCAATCTGGCCGGCAGCCCCGCCATTAAGAAGGGCATCCTGCAGACAGTGAAGGTGGTGGACGAGCTTGTGAAAGTGATGGGCCGGCACAAGCCCGAGAACATCGTGATCGAAATGGCCAGAGAGAACCAGACCACCCAGAAGGGACAGAAGAACAGCCGCGAGAGAATGAAGCGGATCGAAGAGGGCATCAAAGAGCTGGGCAGCCAGATCCTGAAAGAACACCCCGTGGAAAACACCCAGCTGCAGAACGAGAAGCTGTACCTGTACTACCTGCAGAATGGGCGGGATATGTACGTGGACCAGGAACTGGACATCAACCGGCTGTCCGACTACGATGTGGACCATATCGTGCCTCAGAGCTTTCTGAAGGACGACTCCATCGACAACAAGGTGCTGACCAGAAGCGACAAGAACCGGGGCAAGAGCGACAACGTGCCCTCCGAAGAGGTCGTGAAGAAGATGAAGAACTACTGGCGGCAGCTGCTGAACGCCAAGCTGATTACCCAGAGAAAGTTCGACAATCTGACCAAGGCCGAGAGAGGCGGCCTGAGCGAACTGGATAAGGCCGGCTTCATCAAGAGACAGCTGGTGGAAACCCGGCAGATCACAAAGCACGTGGCACAGATCCTGGACTCCCGGATGAACACTAAGTACGACGAGAATGACAAGCTGATCCGGGAAGTGAAAGTGATCACCCTGAAGTCCAAGCTGGTGTCCGATTTCCGGAAGGATTTCCAGTTTTACAAAGTGCGCGAGATCAACAACTACCACCACGCCCACGACGCCTACCTGAACGCCGTCGTGGGAACCGCCCTGATCAAAAAGTACCCTAAGCTGGAAAGCGAGTTCGTGTACGGCGACTACAAGGTGTACGACGTGCGGAAGATGATCGCCAAGAGCGAGCAGGAAATCGGCAAGGCTACCGCCAAGTACTTCTTCTACAGCAACATCATGAACTTTTTCAAGACCGAGATTACCCTGGCCAACGGCGAGATCCGGAAGCGGCCTCTGATCGAGACAAACGGCGAAACCGGGGAGATCGTGTGGGATAAGGGCCGGGATTTTGCCACCGTGCGGAAAGTGCTGAGCATGCCCCAAGTGAATATCGTGAAAAAGACCGAGGTGCAGACAGGCGGCTTCAGCAAAGAGTCTATCCTGCCCAAGAGGAACAGCGATAAGCTGATCGCCAGAAAGAAGGACTGGGACCCTAAGAAGTACGGCGGCTTCGTCAGCCCCACCGTGGCCTATTCTGTGCTGGTGGTGGCCAAAGTGGAAAAGGGCAAGTCCAAGAAACTGAAGAGTGTGAAAGAGCTGCTGGGGATCACCATCATGGAAAGAAGCAGCTTCGAGAAGAATCCCATCGACTTTCTGGAAGCCAAGGGCTACAAAGAAGTGAAAAAGGACCTGATCATCAAGCTGCCTAAGTACTCCCTGTTCGAGCTGGAAAACGGCCGGAAGAGAATGCTGGCCTCTGCCCGCGAACTGCAGAAGGGAAACGAACTGGCCCTGCCCTCCAAATATGTGAACTTCCTGTACCTGGCCAGCCACTATGAGAAGCTGAAGGGCTCCCCCGAGGATAATGAGCAGAAACAGCTGTTTGTGGAACAGCACAAGCACTACCTGGACGAGATCATCGAGCAGATCAGCGAGTTCTCCAAGAGAGTGATCCTGGCCGACGCTAATCTGGACAAAGTGCTGTCCGCCTACAACAAGCACCGGGATAAGCCCATCAGAGAGCAGGCCGAGAATATCATCCACCTGTTTACCCTGACCAATCTGGGAGCCCCTGCCGCCTTCAAGTACTTTGACACCACCATCGACCGGAAGGAGTACAGGAGCACCAAAGAGGTGCTGGACGCCACCCTGATCCACCAGAGCATCACCGGCCTGTACGAGACACGGATCGACCTGTCTCAGCTGGGAGGCGAC**ACTAGTTCCGGCGGCAGCGCTTCTCCAAAGCGTCCGCGTGACCGTCACGATGGAGAATTGGGTGGACGCAAACGTGCAAGAGGTTAAGGTACCTGATTGATCGATAGAGCTCGAATTTCCCCGATCGTTCAAACATTTGGCAATAAAGTTTCTTAAGATTGAATCCTGTTGCCGGTCTTGCGATGATTATCATATAATTTCTGTTGAATTACGTTAAGCATGTAATAATTAACATGTAATGCATGACGTTATTTATGAGATGGGTTTTTATGATTAGAGTCCCGCAATTATACATTTAATACGCGATAGAAAACAAAATATAGCGCGCAAACTAGGATAAATTATCGCGCGCGGTGTCATCTATGTTACTAGATCGG

ABE-P5

GGATCATGAACCAACGGCCTGGCTGTATTTGGTGGTTGTGTAGGGAGATGGGGAGAAGAAAAGCCCGATTCTCTTCGCTGTGATGGGCTGGATGCATGCGGGGGAGCGGGAGGCCCAAGTACGTGCACGGTGAGCGGCCCACAGGGCGAGTGTGAGCGCGAGAGGCGGGAGGAACAGTTTAGTACCACATTGCCCAGCTAACTCGAACGCGACCAACTTATAAACCCGCGCGCTGTCGCTTGTGTgagaccATCTAGACggtctcTGTTTTAGTACTCTGGAAACAGAATCTACTAAAACAAGGCAAAATGCCGTGTTTATCTCGTCAACTTGTTGGCGAGATTTTTTTTTGTTTTTTATGTCTCCCGGGTGCAGCGTGACCCGGTCGTGCCCCTCTCTAGAGATAATGAGCATTGCATGTCTAAGTTATAAAAAATTACCACATATTTTTTTTGTCACACTTGTTTGAAGTGCAGTTTATCTATCTTTATACATATATTTAAACTTTACTCTACGAATAATATAATCTATAGTACTACAATAATATCAGTGTTTTAGAGAATCATATAAATGAACAGTTAGACATGGTCTAAAGGACAATTGAGTATTTTGACAACAGGACTCTACAGTTTTATCTTTTTAGTGTGCATGTGTTCTCCTTTTTTTTTGCAAATAGCTTCACCTATATAATACTTCATCCATTTTATTAGTACATCCATTTAGGGTTTAGGGTTAATGGTTTTTATAGACTAATTTTTTTAGTACATCTATTTTATTCTATTTTAGCCTCTAAATTAAGAAAACTAAAACTCTATTTTAGTTTTTTTATTTAATAATTTAGATATAAAATAGAATAAAATAAAGTGACTAAAAATTAAACAAATACCCTTTAAGAAATTAAAAAAACTAAGGAAACATTTTTCTTGTTTCGAGTAGATAATGCCAGCCTGTTAAACGCCGTCGACGAGTCTAACGGACACCAACCAGCGAACCAGCAGCGTCGCGTCGGGCCAAGCGAAGCAGACGGCACGGCATCTCTGTCGCTGCCTCTGGACCCCTATCGAGAGTTCCGCTCCACCGTTGGACTTGCTCCGCTGTCGGCATCCAGAAATTGCGTGGCGGAGCGGCAGACGTGAGCCGGCACGGCAGGCGGCCTCCTCCTCCTCTCACGGCACGGCAGCTACGGGGGATTCCTTTCCCACCGCTCCTTCGCTTTCCCTTCCTCGCCCGCCGTAATAAATAGACACCCCCTCCACACCCTCTTTCCCCAACCTCGTGTTGTTCGGAGCGCACACACACACAACCAGATCTCCCCCAAATCCACCCGTCGGCACCTCCGCTTCAAGGTACGCCGCTCGTCCTCCCCCCCCCCCCCTCTCTACCTTCTCTAGATCGGCGTTCCGGTCCATGGTTAGGGCCCGGTAGTTCTACTTCTGTTCATGTTTGTGTTAGATCCGTGTTTGTGTTAGATCCGTGCTGCTAGCGTTCGTACACGGATGCGACCTGTACGTCAGACACGTTCTGATTGCTAACTTGCCAGTGTTTCTCTTTGGGGAATCCTGGGATGGCTCTAGCCGTTCCGCAGACGGGATCGATTTCATGATTTTTTTTGTTTCGTTGCATAGGGTTTGGTTTGCCCTTTTCCTTTATTTCAATATATGCCGTGCACTTGTTTGTCGGGTCATCTTTTCATGCTTTTTTTTGTCTTGGTTGTGATGATGTGGTCTGGTTGGGCGGTCGTTCTAGATCGGAGTAGAATTCTGTTTCAAACTACCTGGTGGATTTATTAATTTTGGATCTGTATGTGTGTGCCATACATATTCATAGTTACGAATTGAAGATGATGGATGGAAATATCGATCTAGGATAGGTATACATGTTGATGCGGGTTTTACTGATGCATATACAGAGATGCTTTTTGTTCGCTTGGTTGTGATGATGTGGTGTGGTTGGGCGGTCGTTCATTCGTTCTAGATCGGAGTAGAATACTGTTTCAAACTACCTGGTGTATTTATTAATTTTGGAACTGTATGTGTGTGTCATACATCTTCATAGTTACGAGTTTAAGATGGATGGAAATATCGATCTAGGATAGGTATACATGTTGATGTGGGTTTTACTGATGCATATACATGATGGCATATGCAGCATCTATTCATATGCTCTAACCTTGAGTACCTATCTATTATAATAAACAAGTATGTTTTATAATTATTTTGATCTTGATATACTTGGATGATGGCATATGCAGCAGCTATATGTGGATTTTTTTAGCCCTGCCTTCATACGCTATTTATTTGCTTGGTACTGTTTCTTTTGTCGATGCTCACCCTGTTGTTTGGTGTTACTTCTGCAGCCATGTCCGAAGTCGAGTTTTCCCATGAGTACTGGATGAGACACGCATTGACTCTCGCAAAGAGGGCTTGGGATGAACGCGAGGTGCCCGTGGGGGCAGTACTCGTGCATAACAATCGCGTAATCGGCGAAGGTTGGAATAGGCCGATCGGACGCCACGACCCCACTGCACATGCGGAAATCATGGCCCTTCGACAGGGAGGGCTTGTGATGCAGAATTATCGACTTATCGATGCGACGCTGTACGTCACGCTTGAACCTTGCGTAATGTGCGCGGGAGCTATGATTCACTCCCGCATTGGACGAGTTGTATTCGGTGCCCGCGACGCCAAGACGGGTGCCGCAGGTTCACTGATGGACGTGCTGCATCACCCAGGCATGAACCACCGGGTAGAAATCACAGAAGGCATATTGGCGGACGAATGTGCGGCGCTGTTGTCCGACTTTTTTCGCATGCGGAGGCAGGAGATCAAGGCCCAGAAAAAAGCACAATCCTCTACTGACTCTGGAGGGTCCTCCGGCGGATCGTCCGGCAGCGAGACGCCAGGCACCTCCGAGAGCGCTACGCCTGAATCCTCCGGGGGATCTTCAGGAGGATCATCCGAAGTCGAGTTTTCCCATGAGTACTGGATGAGACACGCATTGACTCTCGCAAAGAGGGCTCGGGATGAACGCGAGGTGCCCGTGGGGGCAGTACTCGTGCTTAACAATCGCGTAATCGGCGAAGGTTGGAATAGGGCGATCGGACTCCACGACCCCACTGCACATGCGGAAATCATGGCCCTTCGACAGGGAGGGCTTGTGATGCAGAATTATCGACTTATCGATGCGACGCTGTACGTCACGTTTGAACCTTGCGTAATGTGCGCGGGAGCTATGATTCACTCCCGCATTGGACGAGTTGTATTCGGTGTCCGCAACGCCAAGACGGGTGCCGCAGGTTCACTGATGGACGTGCTGCATTACCCAGGCATGAACCACCGGGTAGAAATCACAGAAGGCATATTGGCGGACGAATGTGCGGCGCTGTTGTGCTACTTTTTTCGCATGCCGAGGCAGGTGTTCAATGCCCAGAAAAAAGCACAATCCTCTACTGACTCTGGAGGGTCCTCCGGCGGATCGTCCGGCAGCGAGACGCCAGGCACCTCCGAGAGCGCTACGCCTGAATCCTCCGGGGGATCTTCAGGAGGATCAGGATCC**AAGCGGAACTACATCCTGGGCCTGGCCATCGGCATCACCAGCGTGGGCTACGGCATCATCGACTACGAGACACGGGACGTGATCGATGCCGGCGTGCGGCTGTTCAAAGAGGCCAACGTGGAAAACAACGAGGGCAGGCGGAGCAAGAGAGGCGCCAGAAGGCTGAAGCGGCGGAGGCGGCATAGAATCCAGAGAGTGAAGAAGCTGCTGTTCGACTACAACCTGCTGACCGACCACAGCGAGCTGAGCGGCATCAACCCCTACGAGGCCAGAGTGAAGGGCCTGAGCCAGAAGCTGAGCGAGGAAGAGTTCTCTGCCGCCCTGCTGCACCTGGCCAAGAGAAGAGGCGTGCACAACGTGAACGAGGTGGAAGAGGACACCGGCAACGAGCTGTCCACCAAAGAGCAGATCAGCCGGAACAGCAAGGCCCTGGAAGAGAAATACGTGGCCGAACTGCAGCTGGAACGGCTGAAGAAAGACGGCGAAGTGCGGGGCAGCATCAACAGATTCAAGACCAGCGACTACGTGAAAGAAGCCAAACAGCTGCTGAAGGTGCAGAAGGCCTACCACCAGCTGGACCAGAGCTTCATCGACACCTACATCGACCTGCTGGAAACCCGGCGGACCTACTATGAGGGACCTGGCGAGGGCAGCCCCTTCGGCTGGAAGGACATCAAAGAATGGTACGAGATGCTGATGGGCCACTGCACCTACTTCCCCGAGGAACTGCGGAGCGTGAAGTACGCCTACAACGCCGACCTGTACAACGCCCTGAACGACCTGAACAATCTCGTGATCACCAGGGACGAGAACGAGAAGCTGGAATATTACGAGAAGTTCCAGATCATCGAGAACGTGTTCAAGCAGAAGAAGAAGCCCACCCTGAAGCAGATCGCCAAAGAAATCCTCGTGAACGAAGAGGATATTAAGGGCTACAGAGTGACCAGCACCGGCAAGCCCGAGTTCACCAACCTGAAGGTGTACCACGACATCAAGGACATTACCGCCCGGAAAGAGATTATTGAGAACGCCGAGCTGCTGGATCAGATTGCCAAGATCCTGACCATCTACCAGAGCAGCGAGGACATCCAGGAAGAACTGACCAATCTGAACTCCGAGCTGACCCAGGAAGAGATCGAGCAGATCTCTAATCTGAAGGGCTATACCGGCACCCACAACCTGAGCCTGAAGGCCATCAACCTGATCCTGGACGAGCTGTGGCACACCAACGACAACCAGATCGCTATCTTCAACCGGCTGAAGCTGGTGCCCAAGAAGGTGGACCTGTCCCAGCAGAAAGAGATCCCCACCACCCTGGTGGACGACTTCATCCTGAGCCCCGTCGTGAAGAGAAGCTTCATCCAGAGCATCAAAGTGATCAACGCCATCATCAAGAAGTACGGCCTGCCCAACGACATCATTATCGAGCTGGCCCGCGAGAAGAACTCCAAGGACGCCCAGAAAATGATCAACGAGATGCAGAAGCGGAACCGGCAGACCAACGAGCGGATCGAGGAAATCATCCGGACCACCGGCAAAGAGAACGCCAAGTACCTGATCGAGAAGATCAAGCTGCACGACATGCAGGAAGGCAAGTGCCTGTACAGCCTGGAAGCCATCCCTCTGGAAGATCTGCTGAACAACCCCTTCAACTATGAGGTGGACCACATCATCCCCAGAAGCGTGTCCTTCGACAACAGCTTCAACAACAAGGTGCTCGTGAAGCAGGAAGAAAACAGCAAGAAGGGCAACCGGACCCCATTCCAGTACCTGAGCAGCAGCGACAGCAAGATCAGCTACGAAACCTTCAAGAAGCACATCCTGAATCTGGCCAAGGGCAAGGGCAGAATCAGCAAGACCAAGAAAGAGTATCTGCTGGAAGAACGGGACATCAACAGGTTCTCCGTGCAGAAAGACTTCATCAACCGGAACCTGGTGGATACCAGATACGCCACCAGAGGCCTGATGAACCTGCTGCGGAGCTACTTCAGAGTGAACAACCTGGACGTGAAAGTGAAGTCCATCAATGGCGGCTTCACCAGCTTTCTGCGGCGGAAGTGGAAGTTTAAGAAAGAGCGGAACAAGGGGTACAAGCACCACGCCGAGGACGCCCTGATCATTGCCAACGCCGATTTCATCTTCAAAGAGTGGAAGAAACTGGACAAGGCCAAAAAAGTGATGGAAAACCAGATGTTCGAGGAAAAGCAGGCCGAGAGCATGCCCGAGATCGAAACCGAGCAGGAGTACAAAGAGATCTTCATCACCCCCCACCAGATCAAGCACATTAAGGACTTCAAGGACTACAAGTACAGCCACCGGGTGGACAAGAAGCCTAATAGAAAGCTGATTAACGACACCCTGTACTCCACCCGGAAGGACGACAAGGGCAACACCCTGATCGTGAACAATCTGAACGGCCTGTACGACAAGGACAATGACAAGCTGAAAAAGCTGATCAACAAGAGCCCCGAAAAGCTGCTGATGTACCACCACGACCCCCAGACCTACCAGAAACTGAAGCTGATTATGGAACAGTACGGCGACGAGAAGAATCCCCTGTACAAGTACTACGAGGAAACCGGGAACTACCTGACCAAGTACTCCAAAAAGGACAACGGCCCCGTGATCAAGAAGATTAAGTATTACGGCAACAAACTGAACGCCCATCTGGACATCACCGACGACTACCCCAACAGCAGAAACAAGGTCGTGAAGCTGTCCCTGAAGCCCTACAGATTCGACGTGTACCTGGACAATGGCGTGTACAAGTTCGTGACCGTGAAGAATCTGGATGTGATCAAAAAAGAAAACTACTACGAAGTGAATAGCAAGTGCTATGAGGAAGCTAAGAAGCTGAAGAAGATCAGCAACCAGGCCGAGTTTATCGCCTCCTTCTACAAGAACGATCTGATCAAGATCAACGGCGAGCTGTATAGAGTGATCGGCGTGAACAACGACCTGCTGAACCGGATCGAAGTGAACATGATCGACATCACCTACCGCGAGTACCTGGAAAACATGAACGACAAGAGGCCCCCCCACATCATTAAGACAATCGCCTCCAAGACCCAGAGCATTAAGAAGTACAGCACAGACATTCTGGGCAACCTGTATGAAGTGAAATCTAAGAAGCACCCTCAGATCATCAAAAAGGG**ACTAGTTCCGGCGGCAGCGCTTCTCCAAAGCGTCCGCGTGACCGTCACGATGGAGAATTGGGTGGACGCAAACGTGCAAGAGGTTAAGGTACCTGATTGATCGATAGAGCTCGAATTTCCCCGATCGTTCAAACATTTGGCAATAAAGTTTCTTAAGATTGAATCCTGTTGCCGGTCTTGCGATGATTATCATATAATTTCTGTTGAATTACGTTAAGCATGTAATAATTAACATGTAATGCATGACGTTATTTATGAGATGGGTTTTTATGATTAGAGTCCCGCAATTATACATTTAATACGCGATAGAAAACAAAATATAGCGCGCAAACTAGGATAAATTATCGCGCGCGGTGTCATCTATGTTACTAGATCGG

CBE-P1

GGATCATGAACCAACGGCCTGGCTGTATTTGGTGGTTGTGTAGGGAGATGGGGAGAAGAAAAGCCCGATTCTCTTCGCTGTGATGGGCTGGATGCATGCGGGGGAGCGGGAGGCCCAAGTACGTGCACGGTGAGCGGCCCACAGGGCGAGTGTGAGCGCGAGAGGCGGGAGGAACAGTTTAGTACCACATTGCCCAGCTAACTCGAACGCGACCAACTTATAAACCCGCGCGCTGTCGCTTGTGTGAgagaccATCTAGACggtctcTGTTTCAGAGCTATGCTGGAAACAGCATAGCAAGTTGAAATAAGGCTAGTCCGTTATCAACTTGAAAAAGTGGCACCGAGTCGGTGCTTTTTTTGTTTTTTGGCCGGCATGGTCCCAGCCTCCTCGCTGGCGCCGGCTGGGCAACATGCTTCGGCATGGCGAATGGGACTTTTTTTGTTTTTTCCCGGGTGCAGCGTGACCCGGTCGTGCCCCTCTCTAGAGATAATGAGCATTGCATGTCTAAGTTATAAAAAATTACCACATATTTTTTTTGTCACACTTGTTTGAAGTGCAGTTTATCTATCTTTATACATATATTTAAACTTTACTCTACGAATAATATAATCTATAGTACTACAATAATATCAGTGTTTTAGAGAATCATATAAATGAACAGTTAGACATGGTCTAAAGGACAATTGAGTATTTTGACAACAGGACTCTACAGTTTTATCTTTTTAGTGTGCATGTGTTCTCCTTTTTTTTTGCAAATAGCTTCACCTATATAATACTTCATCCATTTTATTAGTACATCCATTTAGGGTTTAGGGTTAATGGTTTTTATAGACTAATTTTTTTAGTACATCTATTTTATTCTATTTTAGCCTCTAAATTAAGAAAACTAAAACTCTATTTTAGTTTTTTTATTTAATAATTTAGATATAAAATAGAATAAAATAAAGTGACTAAAAATTAAACAAATACCCTTTAAGAAATTAAAAAAACTAAGGAAACATTTTTCTTGTTTCGAGTAGATAATGCCAGCCTGTTAAACGCCGTCGACGAGTCTAACGGACACCAACCAGCGAACCAGCAGCGTCGCGTCGGGCCAAGCGAAGCAGACGGCACGGCATCTCTGTCGCTGCCTCTGGACCCCTATCGAGAGTTCCGCTCCACCGTTGGACTTGCTCCGCTGTCGGCATCCAGAAATTGCGTGGCGGAGCGGCAGACGTGAGCCGGCACGGCAGGCGGCCTCCTCCTCCTCTCACGGCACGGCAGCTACGGGGGATTCCTTTCCCACCGCTCCTTCGCTTTCCCTTCCTCGCCCGCCGTAATAAATAGACACCCCCTCCACACCCTCTTTCCCCAACCTCGTGTTGTTCGGAGCGCACACACACACAACCAGATCTCCCCCAAATCCACCCGTCGGCACCTCCGCTTCAAGGTACGCCGCTCGTCCTCCCCCCCCCCCCCTCTCTACCTTCTCTAGATCGGCGTTCCGGTCCATGGTTAGGGCCCGGTAGTTCTACTTCTGTTCATGTTTGTGTTAGATCCGTGTTTGTGTTAGATCCGTGCTGCTAGCGTTCGTACACGGATGCGACCTGTACGTCAGACACGTTCTGATTGCTAACTTGCCAGTGTTTCTCTTTGGGGAATCCTGGGATGGCTCTAGCCGTTCCGCAGACGGGATCGATTTCATGATTTTTTTTGTTTCGTTGCATAGGGTTTGGTTTGCCCTTTTCCTTTATTTCAATATATGCCGTGCACTTGTTTGTCGGGTCATCTTTTCATGCTTTTTTTTGTCTTGGTTGTGATGATGTGGTCTGGTTGGGCGGTCGTTCTAGATCGGAGTAGAATTCTGTTTCAAACTACCTGGTGGATTTATTAATTTTGGATCTGTATGTGTGTGCCATACATATTCATAGTTACGAATTGAAGATGATGGATGGAAATATCGATCTAGGATAGGTATACATGTTGATGCGGGTTTTACTGATGCATATACAGAGATGCTTTTTGTTCGCTTGGTTGTGATGATGTGGTGTGGTTGGGCGGTCGTTCATTCGTTCTAGATCGGAGTAGAATACTGTTTCAAACTACCTGGTGTATTTATTAATTTTGGAACTGTATGTGTGTGTCATACATCTTCATAGTTACGAGTTTAAGATGGATGGAAATATCGATCTAGGATAGGTATACATGTTGATGTGGGTTTTACTGATGCATATACATGATGGCATATGCAGCATCTATTCATATGCTCTAACCTTGAGTACCTATCTATTATAATAAACAAGTATGTTTTATAATTATTTTGATCTTGATATACTTGGATGATGGCATATGCAGCAGCTATATGTGGATTTTTTTAGCCCTGCCTTCATACGCTATTTATTTGCTTGGTACTGTTTCTTTTGTCGATGCTCACCCTGTTGTTTGGTGTTACTTCTGCAG

CCATGGCTCCTAAGAAAAAGAGGAAGGTGGCGAGCTCAGAGACTGGCCCAGTGGCTGTGGACCCCACATTGAGACGGCGGATCGAGCCCCATGAGTTTGAGGTATTCTTCGATCCGAGAGAGCTCCGCAAGGAAACCTGCCTGCTTTACGAAATTAATTGGGGGGGCCGGCACTCCATTTGGCGACATACATCACAGAACACTAACAAGCACGTCGAAGTCAACTTCATCGAGAAGTTCACGACAGAAAGATATTTCTGTCCGAACACAAGGTGCAGCATTACCTGGTTTCTCAGCTGGAGCCCATGCGGCGAATGTAGTAGGGCCATCACTGAGTTCCTGTCAAGGTATCCCCACGTCACTCTGTTTATTTACATCGCAAGGCTGTACCACCACGCTGACCCCCGCAATCGACAAGGCCTGCGGGATTTGATCTCTTCAGGTGTGACTATCCAAATTATGACTGAGCAGGAGTCAGGATACTGCTGGAGAAACTTTGTGAATTATAGCCCGAGTAATGAAGCCCACTGGCCTAGGTATCCCCATCTGTGGGTACGACTGTACGTTCTTGAACTGTACTGCATCATACTGGGCCTGCCTCCTTGTCTCAACATTCTGAGAAGGAAGCAGCCACAGCTGACATTCTTTACCATCGCTCTTCAGTCTTGTCATTACCAGCGACTGCCCCCACACATTCTCTGGGCCACCGGGTTGAAATCCGGCAGCGAGACGCCAGGCACCTCCGAGAGCGCTACGCCTGAATCCGGATCC**GACAAGAAGTACAGCATCGGCCTGGCCATCGGCACCAACTCTGTGGGCTGGGCCGTGATCACCGACGAGTACAAGGTGCCCAGCAAGAAATTCAAGGTGCTGGGCAACACCGACCGGCACAGCATCAAGAAGAACCTGATCGGAGCCCTGCTGTTCGACAGCGGCGAAACAGCCGAGGCCACCCGGCTGAAGAGAACCGCCAGAAGAAGATACACCAGACGGAAGAACCGGATCTGCTATCTGCAAGAGATCTTCAGCAACGAGATGGCCAAGGTGGACGACAGCTTCTTCCACAGACTGGAAGAGTCCTTCCTGGTGGAAGAGGATAAGAAGCACGAGCGGCACCCCATCTTCGGCAACATCGTGGACGAGGTGGCCTACCACGAGAAGTACCCCACCATCTACCACCTGAGAAAGAAACTGGTGGACAGCACCGACAAGGCCGACCTGCGGCTGATCTATCTGGCCCTGGCCCACATGATCAAGTTCCGGGGCCACTTCCTGATCGAGGGCGACCTGAACCCCGACAACAGCGACGTGGACAAGCTGTTCATCCAGCTGGTGCAGACCTACAACCAGCTGTTCGAGGAAAACCCCATCAACGCCAGCGGCGTGGACGCCAAGGCCATCCTGTCTGCCAGACTGAGCAAGAGCAGACGGCTGGAAAATCTGATCGCCCAGCTGCCCGGCGAGAAGAAGAATGGCCTGTTCGGAAACCTGATTGCCCTGAGCCTGGGCCTGACCCCCAACTTCAAGAGCAACTTCGACCTGGCCGAGGATGCCAAACTGCAGCTGAGCAAGGACACCTACGACGACGACCTGGACAACCTGCTGGCCCAGATCGGCGACCAGTACGCCGACCTGTTTCTGGCCGCCAAGAACCTGTCCGACGCCATCCTGCTGAGCGACATCCTGAGAGTGAACACCGAGATCACCAAGGCCCCCCTGAGCGCCTCTATGATCAAGAGATACGACGAGCACCACCAGGACCTGACCCTGCTGAAAGCTCTCGTGCGGCAGCAGCTGCCTGAGAAGTACAAAGAGATTTTCTTCGACCAGAGCAAGAACGGCTACGCCGGCTACATTGACGGCGGAGCCAGCCAGGAAGAGTTCTACAAGTTCATCAAGCCCATCCTGGAAAAGATGGACGGCACCGAGGAACTGCTCGTGAAGCTGAACAGAGAGGACCTGCTGCGGAAGCAGCGGACCTTCGACAACGGCAGCATCCCCCACCAGATCCACCTGGGAGAGCTGCACGCCATTCTGCGGCGGCAGGAAGATTTTTACCCATTCCTGAAGGACAACCGGGAAAAGATCGAGAAGATCCTGACCTTCCGCATCCCCTACTACGTGGGCCCTCTGGCCAGGGGAAACAGCAGATTCGCCTGGATGACCAGAAAGAGCGAGGAAACCATCACCCCCTGGAACTTCGAGGAAGTGGTGGACAAGGGCGCTTCCGCCCAGAGCTTCATCGAGCGGATGACCAACTTCGATAAGAACCTGCCCAACGAGAAGGTGCTGCCCAAGCACAGCCTGCTGTACGAGTACTTCACCGTGTATAACGAGCTGACCAAAGTGAAATACGTGACCGAGGGAATGAGAAAGCCCGCCTTCCTGAGCGGCGAGCAGAAAAAGGCCATCGTGGACCTGCTGTTCAAGACCAACCGGAAAGTGACCGTGAAGCAGCTGAAAGAGGACTACTTCAAGAAAATCGAGTGCTTCGACTCCGTGGAAATCTCCGGCGTGGAAGATCGGTTCAACGCCTCCCTGGGCACATACCACGATCTGCTGAAAATTATCAAGGACAAGGACTTCCTGGACAATGAGGAAAACGAGGACATTCTGGAAGATATCGTGCTGACCCTGACACTGTTTGAGGACAGAGAGATGATCGAGGAACGGCTGAAAACCTATGCCCACCTGTTCGACGACAAAGTGATGAAGCAGCTGAAGCGGCGGAGATACACCGGCTGGGGCAGGCTGAGCCGGAAGCTGATCAACGGCATCCGGGACAAGCAGTCCGGCAAGACAATCCTGGATTTCCTGAAGTCCGACGGCTTCGCCAACAGAAACTTCATGCAGCTGATCCACGACGACAGCCTGACCTTTAAAGAGGACATCCAGAAAGCCCAGGTGTCCGGCCAGGGCGATAGCCTGCACGAGCACATTGCCAATCTGGCCGGCAGCCCCGCCATTAAGAAGGGCATCCTGCAGACAGTGAAGGTGGTGGACGAGCTTGTGAAAGTGATGGGCCGGCACAAGCCCGAGAACATCGTGATCGAAATGGCCAGAGAGAACCAGACCACCCAGAAGGGACAGAAGAACAGCCGCGAGAGAATGAAGCGGATCGAAGAGGGCATCAAAGAGCTGGGCAGCCAGATCCTGAAAGAACACCCCGTGGAAAACACCCAGCTGCAGAACGAGAAGCTGTACCTGTACTACCTGCAGAATGGGCGGGATATGTACGTGGACCAGGAACTGGACATCAACCGGCTGTCCGACTACGATGTGGACCATATCGTGCCTCAGAGCTTTCTGAAGGACGACTCCATCGACAACAAGGTGCTGACCAGAAGCGACAAGAACCGGGGCAAGAGCGACAACGTGCCCTCCGAAGAGGTCGTGAAGAAGATGAAGAACTACTGGCGGCAGCTGCTGAACGCCAAGCTGATTACCCAGAGAAAGTTCGACAATCTGACCAAGGCCGAGAGAGGCGGCCTGAGCGAACTGGATAAGGCCGGCTTCATCAAGAGACAGCTGGTGGAAACCCGGCAGATCACAAAGCACGTGGCACAGATCCTGGACTCCCGGATGAACACTAAGTACGACGAGAATGACAAGCTGATCCGGGAAGTGAAAGTGATCACCCTGAAGTCCAAGCTGGTGTCCGATTTCCGGAAGGATTTCCAGTTTTACAAAGTGCGCGAGATCAACAACTACCACCACGCCCACGACGCCTACCTGAACGCCGTCGTGGGAACCGCCCTGATCAAAAAGTACCCTAAGCTGGAAAGCGAGTTCGTGTACGGCGACTACAAGGTGTACGACGTGCGGAAGATGATCGCCAAGAGCGAGCAGGAAATCGGCAAGGCTACCGCCAAGTACTTCTTCTACAGCAACATCATGAACTTTTTCAAGACCGAGATTACCCTGGCCAACGGCGAGATCCGGAAGCGGCCTCTGATCGAGACAAACGGCGAAACCGGGGAGATCGTGTGGGATAAGGGCCGGGATTTTGCCACCGTGCGGAAAGTGCTGAGCATGCCCCAAGTGAATATCGTGAAAAAGACCGAGGTGCAGACAGGCGGCTTCAGCAAAGAGTCTATCCTGCCCAAGAGGAACAGCGATAAGCTGATCGCCAGAAAGAAGGACTGGGACCCTAAGAAGTACGGCGGCTTCGACAGCCCCACCGTGGCCTATTCTGTGCTGGTGGTGGCCAAAGTGGAAAAGGGCAAGTCCAAGAAACTGAAGAGTGTGAAAGAGCTGCTGGGGATCACCATCATGGAAAGAAGCAGCTTCGAGAAGAATCCCATCGACTTTCTGGAAGCCAAGGGCTACAAAGAAGTGAAAAAGGACCTGATCATCAAGCTGCCTAAGTACTCCCTGTTCGAGCTGGAAAACGGCCGGAAGAGAATGCTGGCCTCTGCCGGCGAACTGCAGAAGGGAAACGAACTGGCCCTGCCCTCCAAATATGTGAACTTCCTGTACCTGGCCAGCCACTATGAGAAGCTGAAGGGCTCCCCCGAGGATAATGAGCAGAAACAGCTGTTTGTGGAACAGCACAAGCACTACCTGGACGAGATCATCGAGCAGATCAGCGAGTTCTCCAAGAGAGTGATCCTGGCCGACGCTAATCTGGACAAAGTGCTGTCCGCCTACAACAAGCACCGGGATAAGCCCATCAGAGAGCAGGCCGAGAATATCATCCACCTGTTTACCCTGACCAATCTGGGAGCCCCTGCCGCCTTCAAGTACTTTGACACCACCATCGACCGGAAGAGGTACACCAGCACCAAAGAGGTGCTGGACGCCACCCTGATCCACCAGAGCATCACCGGCCTGTACGAGACACGGATCGACCTGTCTCAGCTGGGAGGCGAC**ACTAGTTCCGGCGGCAGCACCAACCTGTCCGACATCATCGAGAAGGAGACGGGCAAGCAACTCGTGATCCAGGAGAGCATCCTCATGCTGCCAGAGGAGGTGGAGGAGGTCATCGGCAACAAGCCAGAGTCCGACATCCTGGTGCACACCGCCTACGACGAGTCCACCGACGAGAACGTCATGCTCCTGACCAGCGACGCCCCAGAGTACAAGCCCTGGGCCCTCGTCATCCAGGACAGCAACGGGGAGAACAAGATCAAGATGCTGGCTTCTCCAAAGCGTCCGCGTGACCGTCACGATGGAGAATTGGGTGGACGCAAACGTGCAAGAGGTTAAGGTACCTGATTGATCGATAGAGCTCGAATTTCCCCGATCGTTCAAACATTTGGCAATAAAGTTTCTTAAGATTGAATCCTGTTGCCGGTCTTGCGATGATTATCATATAATTTCTGTTGAATTACGTTAAGCATGTAATAATTAACATGTAATGCATGACGTTATTTATGAGATGGGTTTTTATGATTAGAGTCCCGCAATTATACATTTAATACGCGATAGAAAACAAAATATAGCGCGCAAACTAGGATAAATTATCGCGCGCGGTGTCATCTATGTTACTAGATCGG

CBE-P3

GGATCATGAACCAACGGCCTGGCTGTATTTGGTGGTTGTGTAGGGAGATGGGGAGAAGAAAAGCCCGATTCTCTTCGCTGTGATGGGCTGGATGCATGCGGGGGAGCGGGAGGCCCAAGTACGTGCACGGTGAGCGGCCCACAGGGCGAGTGTGAGCGCGAGAGGCGGGAGGAACAGTTTAGTACCACATTGCCCAGCTAACTCGAACGCGACCAACTTATAAACCCGCGCGCTGTCGCTTGTGTGAgagaccATCTAGACggtctcTGTTTCAGAGCTATGCTGGAAACAGCATAGCAAGTTGAAATAAGGCTAGTCCGTTATCAACTTGAAAAAGTGGCACCGAGTCGGTGCTTTTTTTGTTTTTTGGCCGGCATGGTCCCAGCCTCCTCGCTGGCGCCGGCTGGGCAACATGCTTCGGCATGGCGAATGGGACTTTTTTTGTTTTTTCCCGGGTGCAGCGTGACCCGGTCGTGCCCCTCTCTAGAGATAATGAGCATTGCATGTCTAAGTTATAAAAAATTACCACATATTTTTTTTGTCACACTTGTTTGAAGTGCAGTTTATCTATCTTTATACATATATTTAAACTTTACTCTACGAATAATATAATCTATAGTACTACAATAATATCAGTGTTTTAGAGAATCATATAAATGAACAGTTAGACATGGTCTAAAGGACAATTGAGTATTTTGACAACAGGACTCTACAGTTTTATCTTTTTAGTGTGCATGTGTTCTCCTTTTTTTTTGCAAATAGCTTCACCTATATAATACTTCATCCATTTTATTAGTACATCCATTTAGGGTTTAGGGTTAATGGTTTTTATAGACTAATTTTTTTAGTACATCTATTTTATTCTATTTTAGCCTCTAAATTAAGAAAACTAAAACTCTATTTTAGTTTTTTTATTTAATAATTTAGATATAAAATAGAATAAAATAAAGTGACTAAAAATTAAACAAATACCCTTTAAGAAATTAAAAAAACTAAGGAAACATTTTTCTTGTTTCGAGTAGATAATGCCAGCCTGTTAAACGCCGTCGACGAGTCTAACGGACACCAACCAGCGAACCAGCAGCGTCGCGTCGGGCCAAGCGAAGCAGACGGCACGGCATCTCTGTCGCTGCCTCTGGACCCCTATCGAGAGTTCCGCTCCACCGTTGGACTTGCTCCGCTGTCGGCATCCAGAAATTGCGTGGCGGAGCGGCAGACGTGAGCCGGCACGGCAGGCGGCCTCCTCCTCCTCTCACGGCACGGCAGCTACGGGGGATTCCTTTCCCACCGCTCCTTCGCTTTCCCTTCCTCGCCCGCCGTAATAAATAGACACCCCCTCCACACCCTCTTTCCCCAACCTCGTGTTGTTCGGAGCGCACACACACACAACCAGATCTCCCCCAAATCCACCCGTCGGCACCTCCGCTTCAAGGTACGCCGCTCGTCCTCCCCCCCCCCCCCTCTCTACCTTCTCTAGATCGGCGTTCCGGTCCATGGTTAGGGCCCGGTAGTTCTACTTCTGTTCATGTTTGTGTTAGATCCGTGTTTGTGTTAGATCCGTGCTGCTAGCGTTCGTACACGGATGCGACCTGTACGTCAGACACGTTCTGATTGCTAACTTGCCAGTGTTTCTCTTTGGGGAATCCTGGGATGGCTCTAGCCGTTCCGCAGACGGGATCGATTTCATGATTTTTTTTGTTTCGTTGCATAGGGTTTGGTTTGCCCTTTTCCTTTATTTCAATATATGCCGTGCACTTGTTTGTCGGGTCATCTTTTCATGCTTTTTTTTGTCTTGGTTGTGATGATGTGGTCTGGTTGGGCGGTCGTTCTAGATCGGAGTAGAATTCTGTTTCAAACTACCTGGTGGATTTATTAATTTTGGATCTGTATGTGTGTGCCATACATATTCATAGTTACGAATTGAAGATGATGGATGGAAATATCGATCTAGGATAGGTATACATGTTGATGCGGGTTTTACTGATGCATATACAGAGATGCTTTTTGTTCGCTTGGTTGTGATGATGTGGTGTGGTTGGGCGGTCGTTCATTCGTTCTAGATCGGAGTAGAATACTGTTTCAAACTACCTGGTGTATTTATTAATTTTGGAACTGTATGTGTGTGTCATACATCTTCATAGTTACGAGTTTAAGATGGATGGAAATATCGATCTAGGATAGGTATACATGTTGATGTGGGTTTTACTGATGCATATACATGATGGCATATGCAGCATCTATTCATATGCTCTAACCTTGAGTACCTATCTATTATAATAAACAAGTATGTTTTATAATTATTTTGATCTTGATATACTTGGATGATGGCATATGCAGCAGCTATATGTGGATTTTTTTAGCCCTGCCTTCATACGCTATTTATTTGCTTGGTACTGTTTCTTTTGTCGATGCTCACCCTGTTGTTTGGTGTTACTTCTGCAG

CCATGGCTCCTAAGAAAAAGAGGAAGGTGGCGAGCTCAGAGACTGGCCCAGTGGCTGTGGACCCCACATTGAGACGGCGGATCGAGCCCCATGAGTTTGAGGTATTCTTCGATCCGAGAGAGCTCCGCAAGGAAACCTGCCTGCTTTACGAAATTAATTGGGGGGGCCGGCACTCCATTTGGCGACATACATCACAGAACACTAACAAGCACGTCGAAGTCAACTTCATCGAGAAGTTCACGACAGAAAGATATTTCTGTCCGAACACAAGGTGCAGCATTACCTGGTTTCTCAGCTGGAGCCCATGCGGCGAATGTAGTAGGGCCATCACTGAGTTCCTGTCAAGGTATCCCCACGTCACTCTGTTTATTTACATCGCAAGGCTGTACCACCACGCTGACCCCCGCAATCGACAAGGCCTGCGGGATTTGATCTCTTCAGGTGTGACTATCCAAATTATGACTGAGCAGGAGTCAGGATACTGCTGGAGAAACTTTGTGAATTATAGCCCGAGTAATGAAGCCCACTGGCCTAGGTATCCCCATCTGTGGGTACGACTGTACGTTCTTGAACTGTACTGCATCATACTGGGCCTGCCTCCTTGTCTCAACATTCTGAGAAGGAAGCAGCCACAGCTGACATTCTTTACCATCGCTCTTCAGTCTTGTCATTACCAGCGACTGCCCCCACACATTCTCTGGGCCACCGGGTTGAAATCCGGCAGCGAGACGCCAGGCACCTCCGAGAGCGCTACGCCTGAATCCGGATCC**GACAAGAAGTACAGCATCGGCCTGGCCATCGGCACCAACTCTGTGGGCTGGGCCGTGATCACCGACGAGTACAAGGTGCCCAGCAAGAAATTCAAGGTGCTGGGCAACACCGACCGGCACAGCATCAAGAAGAACCTGATCGGAGCCCTGCTGTTCGACAGCGGCGAAACAGCCGAGGCCACCCGGCTGAAGAGAACCGCCAGAAGAAGATACACCAGACGGAAGAACCGGATCTGCTATCTGCAAGAGATCTTCAGCAACGAGATGGCCAAGGTGGACGACAGCTTCTTCCACAGACTGGAAGAGTCCTTCCTGGTGGAAGAGGATAAGAAGCACGAGCGGCACCCCATCTTCGGCAACATCGTGGACGAGGTGGCCTACCACGAGAAGTACCCCACCATCTACCACCTGAGAAAGAAACTGGTGGACAGCACCGACAAGGCCGACCTGCGGCTGATCTATCTGGCCCTGGCCCACATGATCAAGTTCCGGGGCCACTTCCTGATCGAGGGCGACCTGAACCCCGACAACAGCGACGTGGACAAGCTGTTCATCCAGCTGGTGCAGACCTACAACCAGCTGTTCGAGGAAAACCCCATCAACGCCAGCGGCGTGGACGCCAAGGCCATCCTGTCTGCCAGACTGAGCAAGAGCAGACGGCTGGAAAATCTGATCGCCCAGCTGCCCGGCGAGAAGAAGAATGGCCTGTTCGGAAACCTGATTGCCCTGAGCCTGGGCCTGACCCCCAACTTCAAGAGCAACTTCGACCTGGCCGAGGATGCCAAACTGCAGCTGAGCAAGGACACCTACGACGACGACCTGGACAACCTGCTGGCCCAGATCGGCGACCAGTACGCCGACCTGTTTCTGGCCGCCAAGAACCTGTCCGACGCCATCCTGCTGAGCGACATCCTGAGAGTGAACACCGAGATCACCAAGGCCCCCCTGAGCGCCTCTATGATCAAGAGATACGACGAGCACCACCAGGACCTGACCCTGCTGAAAGCTCTCGTGCGGCAGCAGCTGCCTGAGAAGTACAAAGAGATTTTCTTCGACCAGAGCAAGAACGGCTACGCCGGCTACATTGACGGCGGAGCCAGCCAGGAAGAGTTCTACAAGTTCATCAAGCCCATCCTGGAAAAGATGGACGGCACCGAGGAACTGCTCGTGAAGCTGAACAGAGAGGACCTGCTGCGGAAGCAGCGGACCTTCGACAACGGCAGCATCCCCCACCAGATCCACCTGGGAGAGCTGCACGCCATTCTGCGGCGGCAGGAAGATTTTTACCCATTCCTGAAGGACAACCGGGAAAAGATCGAGAAGATCCTGACCTTCCGCATCCCCTACTACGTGGGCCCTCTGGCCAGGGGAAACAGCAGATTCGCCTGGATGACCAGAAAGAGCGAGGAAACCATCACCCCCTGGAACTTCGAGGAAGTGGTGGACAAGGGCGCTTCCGCCCAGAGCTTCATCGAGCGGATGACCAACTTCGATAAGAACCTGCCCAACGAGAAGGTGCTGCCCAAGCACAGCCTGCTGTACGAGTACTTCACCGTGTATAACGAGCTGACCAAAGTGAAATACGTGACCGAGGGAATGAGAAAGCCCGCCTTCCTGAGCGGCGAGCAGAAAAAGGCCATCGTGGACCTGCTGTTCAAGACCAACCGGAAAGTGACCGTGAAGCAGCTGAAAGAGGACTACTTCAAGAAAATCGAGTGCTTCGACTCCGTGGAAATCTCCGGCGTGGAAGATCGGTTCAACGCCTCCCTGGGCACATACCACGATCTGCTGAAAATTATCAAGGACAAGGACTTCCTGGACAATGAGGAAAACGAGGACATTCTGGAAGATATCGTGCTGACCCTGACACTGTTTGAGGACAGAGAGATGATCGAGGAACGGCTGAAAACCTATGCCCACCTGTTCGACGACAAAGTGATGAAGCAGCTGAAGCGGCGGAGATACACCGGCTGGGGCAGGCTGAGCCGGAAGCTGATCAACGGCATCCGGGACAAGCAGTCCGGCAAGACAATCCTGGATTTCCTGAAGTCCGACGGCTTCGCCAACAGAAACTTCATGCAGCTGATCCACGACGACAGCCTGACCTTTAAAGAGGACATCCAGAAAGCCCAGGTGTCCGGCCAGGGCGATAGCCTGCACGAGCACATTGCCAATCTGGCCGGCAGCCCCGCCATTAAGAAGGGCATCCTGCAGACAGTGAAGGTGGTGGACGAGCTTGTGAAAGTGATGGGCCGGCACAAGCCCGAGAACATCGTGATCGAAATGGCCAGAGAGAACCAGACCACCCAGAAGGGACAGAAGAACAGCCGCGAGAGAATGAAGCGGATCGAAGAGGGCATCAAAGAGCTGGGCAGCCAGATCCTGAAAGAACACCCCGTGGAAAACACCCAGCTGCAGAACGAGAAGCTGTACCTGTACTACCTGCAGAATGGGCGGGATATGTACGTGGACCAGGAACTGGACATCAACCGGCTGTCCGACTACGATGTGGACCATATCGTGCCTCAGAGCTTTCTGAAGGACGACTCCATCGACAACAAGGTGCTGACCAGAAGCGACAAGAACCGGGGCAAGAGCGACAACGTGCCCTCCGAAGAGGTCGTGAAGAAGATGAAGAACTACTGGCGGCAGCTGCTGAACGCCAAGCTGATTACCCAGAGAAAGTTCGACAATCTGACCAAGGCCGAGAGAGGCGGCCTGAGCGAACTGGATAAGGCCGGCTTCATCAAGAGACAGCTGGTGGAAACCCGGCAGATCACAAAGCACGTGGCACAGATCCTGGACTCCCGGATGAACACTAAGTACGACGAGAATGACAAGCTGATCCGGGAAGTGAAAGTGATCACCCTGAAGTCCAAGCTGGTGTCCGATTTCCGGAAGGATTTCCAGTTTTACAAAGTGCGCGAGATCAACAACTACCACCACGCCCACGACGCCTACCTGAACGCCGTCGTGGGAACCGCCCTGATCAAAAAGTACCCTAAGCTGGAAAGCGAGTTCGTGTACGGCGACTACAAGGTGTACGACGTGCGGAAGATGATCGCCAAGAGCGAGCAGGAAATCGGCAAGGCTACCGCCAAGTACTTCTTCTACAGCAACATCATGAACTTTTTCAAGACCGAGATTACCCTGGCCAACGGCGAGATCCGGAAGCGGCCTCTGATCGAGACAAACGGCGAAACCGGGGAGATCGTGTGGGATAAGGGCCGGGATTTTGCCACCGTGCGGAAAGTGCTGAGCATGCCCCAAGTGAATATCGTGAAAAAGACCGAGGTGCAGACAGGCGGCTTCAGCAAAGAGTCTATCCTGCCCAAGAGGAACAGCGATAAGCTGATCGCCAGAAAGAAGGACTGGGACCCTAAGAAGTACGGCGGCTTCGTCAGCCCCACCGTGGCCTATTCTGTGCTGGTGGTGGCCAAAGTGGAAAAGGGCAAGTCCAAGAAACTGAAGAGTGTGAAAGAGCTGCTGGGGATCACCATCATGGAAAGAAGCAGCTTCGAGAAGAATCCCATCGACTTTCTGGAAGCCAAGGGCTACAAAGAAGTGAAAAAGGACCTGATCATCAAGCTGCCTAAGTACTCCCTGTTCGAGCTGGAAAACGGCCGGAAGAGAATGCTGGCCTCTGCCGGCGAACTGCAGAAGGGAAACGAACTGGCCCTGCCCTCCAAATATGTGAACTTCCTGTACCTGGCCAGCCACTATGAGAAGCTGAAGGGCTCCCCCGAGGATAATGAGCAGAAACAGCTGTTTGTGGAACAGCACAAGCACTACCTGGACGAGATCATCGAGCAGATCAGCGAGTTCTCCAAGAGAGTGATCCTGGCCGACGCTAATCTGGACAAAGTGCTGTCCGCCTACAACAAGCACCGGGATAAGCCCATCAGAGAGCAGGCCGAGAATATCATCCACCTGTTTACCCTGACCAATCTGGGAGCCCCTGCCGCCTTCAAGTACTTTGACACCACCATCGACCGGAAGCAGTACAGGAGCACCAAAGAGGTGCTGGACGCCACCCTGATCCACCAGAGCATCACCGGCCTGTACGAGACACGGATCGACCTGTCTCAGCTGGGAGGCGAC**ACTAGTTCCGGCGGCAGCACCAACCTGTCCGACATCATCGAGAAGGAGACGGGCAAGCAACTCGTGATCCAGGAGAGCATCCTCATGCTGCCAGAGGAGGTGGAGGAGGTCATCGGCAACAAGCCAGAGTCCGACATCCTGGTGCACACCGCCTACGACGAGTCCACCGACGAGAACGTCATGCTCCTGACCAGCGACGCCCCAGAGTACAAGCCCTGGGCCCTCGTCATCCAGGACAGCAACGGGGAGAACAAGATCAAGATGCTGGCTTCTCCAAAGCGTCCGCGTGACCGTCACGATGGAGAATTGGGTGGACGCAAACGTGCAAGAGGTTAAGGTACCTGATTGATCGATAGAGCTCGAATTTCCCCGATCGTTCAAACATTTGGCAATAAAGTTTCTTAAGATTGAATCCTGTTGCCGGTCTTGCGATGATTATCATATAATTTCTGTTGAATTACGTTAAGCATGTAATAATTAACATGTAATGCATGACGTTATTTATGAGATGGGTTTTTATGATTAGAGTCCCGCAATTATACATTTAATACGCGATAGAAAACAAAATATAGCGCGCAAACTAGGATAAATTATCGCGCGCGGTGTCATCTATGTTACTAGATCGG

CBE-P5

GGATCATGAACCAACGGCCTGGCTGTATTTGGTGGTTGTGTAGGGAGATGGGGAGAAGAAAAGCCCGATTCTCTTCGCTGTGATGGGCTGGATGCATGCGGGGGAGCGGGAGGCCCAAGTACGTGCACGGTGAGCGGCCCACAGGGCGAGTGTGAGCGCGAGAGGCGGGAGGAACAGTTTAGTACCACATTGCCCAGCTAACTCGAACGCGACCAACTTATAAACCCGCGCGCTGTCGCTTGTGTgagaccATCTAGACggtctcTGTTTTAGTACTCTGGAAACAGAATCTACTAAAACAAGGCAAAATGCCGTGTTTATCTCGTCAACTTGTTGGCGAGATTTTTTTTTGTTTTTTATGTCTCCCGGGTGCAGCGTGACCCGGTCGTGCCCCTCTCTAGAGATAATGAGCATTGCATGTCTAAGTTATAAAAAATTACCACATATTTTTTTTGTCACACTTGTTTGAAGTGCAGTTTATCTATCTTTATACATATATTTAAACTTTACTCTACGAATAATATAATCTATAGTACTACAATAATATCAGTGTTTTAGAGAATCATATAAATGAACAGTTAGACATGGTCTAAAGGACAATTGAGTATTTTGACAACAGGACTCTACAGTTTTATCTTTTTAGTGTGCATGTGTTCTCCTTTTTTTTTGCAAATAGCTTCACCTATATAATACTTCATCCATTTTATTAGTACATCCATTTAGGGTTTAGGGTTAATGGTTTTTATAGACTAATTTTTTTAGTACATCTATTTTATTCTATTTTAGCCTCTAAATTAAGAAAACTAAAACTCTATTTTAGTTTTTTTATTTAATAATTTAGATATAAAATAGAATAAAATAAAGTGACTAAAAATTAAACAAATACCCTTTAAGAAATTAAAAAAACTAAGGAAACATTTTTCTTGTTTCGAGTAGATAATGCCAGCCTGTTAAACGCCGTCGACGAGTCTAACGGACACCAACCAGCGAACCAGCAGCGTCGCGTCGGGCCAAGCGAAGCAGACGGCACGGCATCTCTGTCGCTGCCTCTGGACCCCTATCGAGAGTTCCGCTCCACCGTTGGACTTGCTCCGCTGTCGGCATCCAGAAATTGCGTGGCGGAGCGGCAGACGTGAGCCGGCACGGCAGGCGGCCTCCTCCTCCTCTCACGGCACGGCAGCTACGGGGGATTCCTTTCCCACCGCTCCTTCGCTTTCCCTTCCTCGCCCGCCGTAATAAATAGACACCCCCTCCACACCCTCTTTCCCCAACCTCGTGTTGTTCGGAGCGCACACACACACAACCAGATCTCCCCCAAATCCACCCGTCGGCACCTCCGCTTCAAGGTACGCCGCTCGTCCTCCCCCCCCCCCCCTCTCTACCTTCTCTAGATCGGCGTTCCGGTCCATGGTTAGGGCCCGGTAGTTCTACTTCTGTTCATGTTTGTGTTAGATCCGTGTTTGTGTTAGATCCGTGCTGCTAGCGTTCGTACACGGATGCGACCTGTACGTCAGACACGTTCTGATTGCTAACTTGCCAGTGTTTCTCTTTGGGGAATCCTGGGATGGCTCTAGCCGTTCCGCAGACGGGATCGATTTCATGATTTTTTTTGTTTCGTTGCATAGGGTTTGGTTTGCCCTTTTCCTTTATTTCAATATATGCCGTGCACTTGTTTGTCGGGTCATCTTTTCATGCTTTTTTTTGTCTTGGTTGTGATGATGTGGTCTGGTTGGGCGGTCGTTCTAGATCGGAGTAGAATTCTGTTTCAAACTACCTGGTGGATTTATTAATTTTGGATCTGTATGTGTGTGCCATACATATTCATAGTTACGAATTGAAGATGATGGATGGAAATATCGATCTAGGATAGGTATACATGTTGATGCGGGTTTTACTGATGCATATACAGAGATGCTTTTTGTTCGCTTGGTTGTGATGATGTGGTGTGGTTGGGCGGTCGTTCATTCGTTCTAGATCGGAGTAGAATACTGTTTCAAACTACCTGGTGTATTTATTAATTTTGGAACTGTATGTGTGTGTCATACATCTTCATAGTTACGAGTTTAAGATGGATGGAAATATCGATCTAGGATAGGTATACATGTTGATGTGGGTTTTACTGATGCATATACATGATGGCATATGCAGCATCTATTCATATGCTCTAACCTTGAGTACCTATCTATTATAATAAACAAGTATGTTTTATAATTATTTTGATCTTGATATACTTGGATGATGGCATATGCAGCAGCTATATGTGGATTTTTTTAGCCCTGCCTTCATACGCTATTTATTTGCTTGGTACTGTTTCTTTTGTCGATGCTCACCCTGTTGTTTGGTGTTACTTCTGCAGCCATGGCTCCTAAGAAAAAGAGGAAGGTGGCGAGCTCAGAGACTGGCCCAGTGGCTGTGGACCCCACATTGAGACGGCGGATCGAGCCCCATGAGTTTGAGGTATTCTTCGATCCGAGAGAGCTCCGCAAGGAAACCTGCCTGCTTTACGAAATTAATTGGGGGGGCCGGCACTCCATTTGGCGACATACATCACAGAACACTAACAAGCACGTCGAAGTCAACTTCATCGAGAAGTTCACGACAGAAAGATATTTCTGTCCGAACACAAGGTGCAGCATTACCTGGTTTCTCAGCTGGAGCCCATGCGGCGAATGTAGTAGGGCCATCACTGAGTTCCTGTCAAGGTATCCCCACGTCACTCTGTTTATTTACATCGCAAGGCTGTACCACCACGCTGACCCCCGCAATCGACAAGGCCTGCGGGATTTGATCTCTTCAGGTGTGACTATCCAAATTATGACTGAGCAGGAGTCAGGATACTGCTGGAGAAACTTTGTGAATTATAGCCCGAGTAATGAAGCCCACTGGCCTAGGTATCCCCATCTGTGGGTACGACTGTACGTTCTTGAACTGTACTGCATCATACTGGGCCTGCCTCCTTGTCTCAACATTCTGAGAAGGAAGCAGCCACAGCTGACATTCTTTACCATCGCTCTTCAGTCTTGTCATTACCAGCGACTGCCCCCACACATTCTCTGGGCCACCGGGTTGAAATCCGGCAGCGAGACGCCAGGCACCTCCGAGAGCGCTACGCCTGAATCCGGATCC**AAGCGGAACTACATCCTGGGCCTGGCCATCGGCATCACCAGCGTGGGCTACGGCATCATCGACTACGAGACACGGGACGTGATCGATGCCGGCGTGCGGCTGTTCAAAGAGGCCAACGTGGAAAACAACGAGGGCAGGCGGAGCAAGAGAGGCGCCAGAAGGCTGAAGCGGCGGAGGCGGCATAGAATCCAGAGAGTGAAGAAGCTGCTGTTCGACTACAACCTGCTGACCGACCACAGCGAGCTGAGCGGCATCAACCCCTACGAGGCCAGAGTGAAGGGCCTGAGCCAGAAGCTGAGCGAGGAAGAGTTCTCTGCCGCCCTGCTGCACCTGGCCAAGAGAAGAGGCGTGCACAACGTGAACGAGGTGGAAGAGGACACCGGCAACGAGCTGTCCACCAAAGAGCAGATCAGCCGGAACAGCAAGGCCCTGGAAGAGAAATACGTGGCCGAACTGCAGCTGGAACGGCTGAAGAAAGACGGCGAAGTGCGGGGCAGCATCAACAGATTCAAGACCAGCGACTACGTGAAAGAAGCCAAACAGCTGCTGAAGGTGCAGAAGGCCTACCACCAGCTGGACCAGAGCTTCATCGACACCTACATCGACCTGCTGGAAACCCGGCGGACCTACTATGAGGGACCTGGCGAGGGCAGCCCCTTCGGCTGGAAGGACATCAAAGAATGGTACGAGATGCTGATGGGCCACTGCACCTACTTCCCCGAGGAACTGCGGAGCGTGAAGTACGCCTACAACGCCGACCTGTACAACGCCCTGAACGACCTGAACAATCTCGTGATCACCAGGGACGAGAACGAGAAGCTGGAATATTACGAGAAGTTCCAGATCATCGAGAACGTGTTCAAGCAGAAGAAGAAGCCCACCCTGAAGCAGATCGCCAAAGAAATCCTCGTGAACGAAGAGGATATTAAGGGCTACAGAGTGACCAGCACCGGCAAGCCCGAGTTCACCAACCTGAAGGTGTACCACGACATCAAGGACATTACCGCCCGGAAAGAGATTATTGAGAACGCCGAGCTGCTGGATCAGATTGCCAAGATCCTGACCATCTACCAGAGCAGCGAGGACATCCAGGAAGAACTGACCAATCTGAACTCCGAGCTGACCCAGGAAGAGATCGAGCAGATCTCTAATCTGAAGGGCTATACCGGCACCCACAACCTGAGCCTGAAGGCCATCAACCTGATCCTGGACGAGCTGTGGCACACCAACGACAACCAGATCGCTATCTTCAACCGGCTGAAGCTGGTGCCCAAGAAGGTGGACCTGTCCCAGCAGAAAGAGATCCCCACCACCCTGGTGGACGACTTCATCCTGAGCCCCGTCGTGAAGAGAAGCTTCATCCAGAGCATCAAAGTGATCAACGCCATCATCAAGAAGTACGGCCTGCCCAACGACATCATTATCGAGCTGGCCCGCGAGAAGAACTCCAAGGACGCCCAGAAAATGATCAACGAGATGCAGAAGCGGAACCGGCAGACCAACGAGCGGATCGAGGAAATCATCCGGACCACCGGCAAAGAGAACGCCAAGTACCTGATCGAGAAGATCAAGCTGCACGACATGCAGGAAGGCAAGTGCCTGTACAGCCTGGAAGCCATCCCTCTGGAAGATCTGCTGAACAACCCCTTCAACTATGAGGTGGACCACATCATCCCCAGAAGCGTGTCCTTCGACAACAGCTTCAACAACAAGGTGCTCGTGAAGCAGGAAGAAAACAGCAAGAAGGGCAACCGGACCCCATTCCAGTACCTGAGCAGCAGCGACAGCAAGATCAGCTACGAAACCTTCAAGAAGCACATCCTGAATCTGGCCAAGGGCAAGGGCAGAATCAGCAAGACCAAGAAAGAGTATCTGCTGGAAGAACGGGACATCAACAGGTTCTCCGTGCAGAAAGACTTCATCAACCGGAACCTGGTGGATACCAGATACGCCACCAGAGGCCTGATGAACCTGCTGCGGAGCTACTTCAGAGTGAACAACCTGGACGTGAAAGTGAAGTCCATCAATGGCGGCTTCACCAGCTTTCTGCGGCGGAAGTGGAAGTTTAAGAAAGAGCGGAACAAGGGGTACAAGCACCACGCCGAGGACGCCCTGATCATTGCCAACGCCGATTTCATCTTCAAAGAGTGGAAGAAACTGGACAAGGCCAAAAAAGTGATGGAAAACCAGATGTTCGAGGAAAAGCAGGCCGAGAGCATGCCCGAGATCGAAACCGAGCAGGAGTACAAAGAGATCTTCATCACCCCCCACCAGATCAAGCACATTAAGGACTTCAAGGACTACAAGTACAGCCACCGGGTGGACAAGAAGCCTAATAGAAAGCTGATTAACGACACCCTGTACTCCACCCGGAAGGACGACAAGGGCAACACCCTGATCGTGAACAATCTGAACGGCCTGTACGACAAGGACAATGACAAGCTGAAAAAGCTGATCAACAAGAGCCCCGAAAAGCTGCTGATGTACCACCACGACCCCCAGACCTACCAGAAACTGAAGCTGATTATGGAACAGTACGGCGACGAGAAGAATCCCCTGTACAAGTACTACGAGGAAACCGGGAACTACCTGACCAAGTACTCCAAAAAGGACAACGGCCCCGTGATCAAGAAGATTAAGTATTACGGCAACAAACTGAACGCCCATCTGGACATCACCGACGACTACCCCAACAGCAGAAACAAGGTCGTGAAGCTGTCCCTGAAGCCCTACAGATTCGACGTGTACCTGGACAATGGCGTGTACAAGTTCGTGACCGTGAAGAATCTGGATGTGATCAAAAAAGAAAACTACTACGAAGTGAATAGCAAGTGCTATGAGGAAGCTAAGAAGCTGAAGAAGATCAGCAACCAGGCCGAGTTTATCGCCTCCTTCTACAAGAACGATCTGATCAAGATCAACGGCGAGCTGTATAGAGTGATCGGCGTGAACAACGACCTGCTGAACCGGATCGAAGTGAACATGATCGACATCACCTACCGCGAGTACCTGGAAAACATGAACGACAAGAGGCCCCCCCACATCATTAAGACAATCGCCTCCAAGACCCAGAGCATTAAGAAGTACAGCACAGACATTCTGGGCAACCTGTATGAAGTGAAATCTAAGAAGCACCCTCAGATCATCAAAAAGGG**ACTAGTTCCGGCGGCAGCACCAACCTGTCCGACATCATCGAGAAGGAGACGGGCAAGCAACTCGTGATCCAGGAGAGCATCCTCATGCTGCCAGAGGAGGTGGAGGAGGTCATCGGCAACAAGCCAGAGTCCGACATCCTGGTGCACACCGCCTACGACGAGTCCACCGACGAGAACGTCATGCTCCTGACCAGCGACGCCCCAGAGTACAAGCCCTGGGCCCTCGTCATCCAGGACAGCAACGGGGAGAACAAGATCAAGATGCTGGCTTCTCCAAAGCGTCCGCGTGACCGTCACGATGGAGAATTGGGTGGACGCAAACGTGCAAGAGGTTAAGGTACCTGATTGATCGATAGAGCTCGAATTTCCCCGATCGTTCAAACATTTGGCAATAAAGTTTCTTAAGATTGAATCCTGTTGCCGGTCTTGCGATGATTATCATATAATTTCTGTTGAATTACGTTAAGCATGTAATAATTAACATGTAATGCATGACGTTATTTATGAGATGGGTTTTTATGATTAGAGTCCCGCAATTATACATTTAATACGCGATAGAAAACAAAATATAGCGCGCAAACTAGGATAAATTATCGCGCGCGGTGTCATCTATGTTACTAGATCGG

pVQR-Cas9

GGATCATGAACCAACGGCCTGGCTGTATTTGGTGGTTGTGTAGGGAGATGGGGAGAAGAAAAGCCCGATTCTCTTCGCTGTGATGGGCTGGATGCATGCGGGGGAGCGGGAGGCCCAAGTACGTGCACGGTGAGCGGCCCACAGGGCGAGTGTGAGCGCGAGAGGCGGGAGGAACAGTTTAGTACCACATTGCCCAGCTAACTCGAACGCGACCAACTTATAAACCCGCGCGCTGTCGCTTGTGTGAgagaccATCTAGACggtctcTGTTTCAGAGCTATGCTGGAAACAGCATAGCAAGTTGAAATAAGGCTAGTCCGTTATCAACTTGAAAAAGTGGCACCGAGTCGGTGCTTTTTTTGTTTTTTGGCCGGCATGGTCCCAGCCTCCTCGCTGGCGCCGGCTGGGCAACATGCTTCGGCATGGCGAATGGGACTTTTTTTGTTTTTTCCCGGGTGCAGCGTGACCCGGTCGTGCCCCTCTCTAGAGATAATGAGCATTGCATGTCTAAGTTATAAAAAATTACCACATATTTTTTTTGTCACACTTGTTTGAAGTGCAGTTTATCTATCTTTATACATATATTTAAACTTTACTCTACGAATAATATAATCTATAGTACTACAATAATATCAGTGTTTTAGAGAATCATATAAATGAACAGTTAGACATGGTCTAAAGGACAATTGAGTATTTTGACAACAGGACTCTACAGTTTTATCTTTTTAGTGTGCATGTGTTCTCCTTTTTTTTTGCAAATAGCTTCACCTATATAATACTTCATCCATTTTATTAGTACATCCATTTAGGGTTTAGGGTTAATGGTTTTTATAGACTAATTTTTTTAGTACATCTATTTTATTCTATTTTAGCCTCTAAATTAAGAAAACTAAAACTCTATTTTAGTTTTTTTATTTAATAATTTAGATATAAAATAGAATAAAATAAAGTGACTAAAAATTAAACAAATACCCTTTAAGAAATTAAAAAAACTAAGGAAACATTTTTCTTGTTTCGAGTAGATAATGCCAGCCTGTTAAACGCCGTCGACGAGTCTAACGGACACCAACCAGCGAACCAGCAGCGTCGCGTCGGGCCAAGCGAAGCAGACGGCACGGCATCTCTGTCGCTGCCTCTGGACCCCTATCGAGAGTTCCGCTCCACCGTTGGACTTGCTCCGCTGTCGGCATCCAGAAATTGCGTGGCGGAGCGGCAGACGTGAGCCGGCACGGCAGGCGGCCTCCTCCTCCTCTCACGGCACGGCAGCTACGGGGGATTCCTTTCCCACCGCTCCTTCGCTTTCCCTTCCTCGCCCGCCGTAATAAATAGACACCCCCTCCACACCCTCTTTCCCCAACCTCGTGTTGTTCGGAGCGCACACACACACAACCAGATCTCCCCCAAATCCACCCGTCGGCACCTCCGCTTCAAGGTACGCCGCTCGTCCTCCCCCCCCCCCCCTCTCTACCTTCTCTAGATCGGCGTTCCGGTCCATGGTTAGGGCCCGGTAGTTCTACTTCTGTTCATGTTTGTGTTAGATCCGTGTTTGTGTTAGATCCGTGCTGCTAGCGTTCGTACACGGATGCGACCTGTACGTCAGACACGTTCTGATTGCTAACTTGCCAGTGTTTCTCTTTGGGGAATCCTGGGATGGCTCTAGCCGTTCCGCAGACGGGATCGATTTCATGATTTTTTTTGTTTCGTTGCATAGGGTTTGGTTTGCCCTTTTCCTTTATTTCAATATATGCCGTGCACTTGTTTGTCGGGTCATCTTTTCATGCTTTTTTTTGTCTTGGTTGTGATGATGTGGTCTGGTTGGGCGGTCGTTCTAGATCGGAGTAGAATTCTGTTTCAAACTACCTGGTGGATTTATTAATTTTGGATCTGTATGTGTGTGCCATACATATTCATAGTTACGAATTGAAGATGATGGATGGAAATATCGATCTAGGATAGGTATACATGTTGATGCGGGTTTTACTGATGCATATACAGAGATGCTTTTTGTTCGCTTGGTTGTGATGATGTGGTGTGGTTGGGCGGTCGTTCATTCGTTCTAGATCGGAGTAGAATACTGTTTCAAACTACCTGGTGTATTTATTAATTTTGGAACTGTATGTGTGTGTCATACATCTTCATAGTTACGAGTTTAAGATGGATGGAAATATCGATCTAGGATAGGTATACATGTTGATGTGGGTTTTACTGATGCATATACATGATGGCATATGCAGCATCTATTCATATGCTCTAACCTTGAGTACCTATCTATTATAATAAACAAGTATGTTTTATAATTATTTTGATCTTGATATACTTGGATGATGGCATATGCAGCAGCTATATGTGGATTTTTTTAGCCCTGCCTTCATACGCTATTTATTTGCTTGGTACTGTTTCTTTTGTCGATGCTCACCCTGTTGTTTGGTGTTACTTCTGCAG

CCATGGCTCCTAAGAAAAAGAGGAAGGTGGCG**GACAAGAAGTACAGCATCGGCCTGGACATCGGCACCAACTCTGTGGGCTGGGCCGTGATCACCGACGAGTACAAGGTGCCCAGCAAGAAATTCAAGGTGCTGGGCAACACCGACCGGCACAGCATCAAGAAGAACCTGATCGGAGCCCTGCTGTTCGACAGCGGCGAAACAGCCGAGGCCACCCGGCTGAAGAGAACCGCCAGAAGAAGATACACCAGACGGAAGAACCGGATCTGCTATCTGCAAGAGATCTTCAGCAACGAGATGGCCAAGGTGGACGACAGCTTCTTCCACAGACTGGAAGAGTCCTTCCTGGTGGAAGAGGATAAGAAGCACGAGCGGCACCCCATCTTCGGCAACATCGTGGACGAGGTGGCCTACCACGAGAAGTACCCCACCATCTACCACCTGAGAAAGAAACTGGTGGACAGCACCGACAAGGCCGACCTGCGGCTGATCTATCTGGCCCTGGCCCACATGATCAAGTTCCGGGGCCACTTCCTGATCGAGGGCGACCTGAACCCCGACAACAGCGACGTGGACAAGCTGTTCATCCAGCTGGTGCAGACCTACAACCAGCTGTTCGAGGAAAACCCCATCAACGCCAGCGGCGTGGACGCCAAGGCCATCCTGTCTGCCAGACTGAGCAAGAGCAGACGGCTGGAAAATCTGATCGCCCAGCTGCCCGGCGAGAAGAAGAATGGCCTGTTCGGAAACCTGATTGCCCTGAGCCTGGGCCTGACCCCCAACTTCAAGAGCAACTTCGACCTGGCCGAGGATGCCAAACTGCAGCTGAGCAAGGACACCTACGACGACGACCTGGACAACCTGCTGGCCCAGATCGGCGACCAGTACGCCGACCTGTTTCTGGCCGCCAAGAACCTGTCCGACGCCATCCTGCTGAGCGACATCCTGAGAGTGAACACCGAGATCACCAAGGCCCCCCTGAGCGCCTCTATGATCAAGAGATACGACGAGCACCACCAGGACCTGACCCTGCTGAAAGCTCTCGTGCGGCAGCAGCTGCCTGAGAAGTACAAAGAGATTTTCTTCGACCAGAGCAAGAACGGCTACGCCGGCTACATTGACGGCGGAGCCAGCCAGGAAGAGTTCTACAAGTTCATCAAGCCCATCCTGGAAAAGATGGACGGCACCGAGGAACTGCTCGTGAAGCTGAACAGAGAGGACCTGCTGCGGAAGCAGCGGACCTTCGACAACGGCAGCATCCCCCACCAGATCCACCTGGGAGAGCTGCACGCCATTCTGCGGCGGCAGGAAGATTTTTACCCATTCCTGAAGGACAACCGGGAAAAGATCGAGAAGATCCTGACCTTCCGCATCCCCTACTACGTGGGCCCTCTGGCCAGGGGAAACAGCAGATTCGCCTGGATGACCAGAAAGAGCGAGGAAACCATCACCCCCTGGAACTTCGAGGAAGTGGTGGACAAGGGCGCTTCCGCCCAGAGCTTCATCGAGCGGATGACCAACTTCGATAAGAACCTGCCCAACGAGAAGGTGCTGCCCAAGCACAGCCTGCTGTACGAGTACTTCACCGTGTATAACGAGCTGACCAAAGTGAAATACGTGACCGAGGGAATGAGAAAGCCCGCCTTCCTGAGCGGCGAGCAGAAAAAGGCCATCGTGGACCTGCTGTTCAAGACCAACCGGAAAGTGACCGTGAAGCAGCTGAAAGAGGACTACTTCAAGAAAATCGAGTGCTTCGACTCCGTGGAAATCTCCGGCGTGGAAGATCGGTTCAACGCCTCCCTGGGCACATACCACGATCTGCTGAAAATTATCAAGGACAAGGACTTCCTGGACAATGAGGAAAACGAGGACATTCTGGAAGATATCGTGCTGACCCTGACACTGTTTGAGGACAGAGAGATGATCGAGGAACGGCTGAAAACCTATGCCCACCTGTTCGACGACAAAGTGATGAAGCAGCTGAAGCGGCGGAGATACACCGGCTGGGGCAGGCTGAGCCGGAAGCTGATCAACGGCATCCGGGACAAGCAGTCCGGCAAGACAATCCTGGATTTCCTGAAGTCCGACGGCTTCGCCAACAGAAACTTCATGCAGCTGATCCACGACGACAGCCTGACCTTTAAAGAGGACATCCAGAAAGCCCAGGTGTCCGGCCAGGGCGATAGCCTGCACGAGCACATTGCCAATCTGGCCGGCAGCCCCGCCATTAAGAAGGGCATCCTGCAGACAGTGAAGGTGGTGGACGAGCTTGTGAAAGTGATGGGCCGGCACAAGCCCGAGAACATCGTGATCGAAATGGCCAGAGAGAACCAGACCACCCAGAAGGGACAGAAGAACAGCCGCGAGAGAATGAAGCGGATCGAAGAGGGCATCAAAGAGCTGGGCAGCCAGATCCTGAAAGAACACCCCGTGGAAAACACCCAGCTGCAGAACGAGAAGCTGTACCTGTACTACCTGCAGAATGGGCGGGATATGTACGTGGACCAGGAACTGGACATCAACCGGCTGTCCGACTACGATGTGGACCATATCGTGCCTCAGAGCTTTCTGAAGGACGACTCCATCGACAACAAGGTGCTGACCAGAAGCGACAAGAACCGGGGCAAGAGCGACAACGTGCCCTCCGAAGAGGTCGTGAAGAAGATGAAGAACTACTGGCGGCAGCTGCTGAACGCCAAGCTGATTACCCAGAGAAAGTTCGACAATCTGACCAAGGCCGAGAGAGGCGGCCTGAGCGAACTGGATAAGGCCGGCTTCATCAAGAGACAGCTGGTGGAAACCCGGCAGATCACAAAGCACGTGGCACAGATCCTGGACTCCCGGATGAACACTAAGTACGACGAGAATGACAAGCTGATCCGGGAAGTGAAAGTGATCACCCTGAAGTCCAAGCTGGTGTCCGATTTCCGGAAGGATTTCCAGTTTTACAAAGTGCGCGAGATCAACAACTACCACCACGCCCACGACGCCTACCTGAACGCCGTCGTGGGAACCGCCCTGATCAAAAAGTACCCTAAGCTGGAAAGCGAGTTCGTGTACGGCGACTACAAGGTGTACGACGTGCGGAAGATGATCGCCAAGAGCGAGCAGGAAATCGGCAAGGCTACCGCCAAGTACTTCTTCTACAGCAACATCATGAACTTTTTCAAGACCGAGATTACCCTGGCCAACGGCGAGATCCGGAAGCGGCCTCTGATCGAGACAAACGGCGAAACCGGGGAGATCGTGTGGGATAAGGGCCGGGATTTTGCCACCGTGCGGAAAGTGCTGAGCATGCCCCAAGTGAATATCGTGAAAAAGACCGAGGTGCAGACAGGCGGCTTCAGCAAAGAGTCTATCCTGCCCAAGAGGAACAGCGATAAGCTGATCGCCAGAAAGAAGGACTGGGACCCTAAGAAGTACGGCGGCTTCGTCAGCCCCACCGTGGCCTATTCTGTGCTGGTGGTGGCCAAAGTGGAAAAGGGCAAGTCCAAGAAACTGAAGAGTGTGAAAGAGCTGCTGGGGATCACCATCATGGAAAGAAGCAGCTTCGAGAAGAATCCCATCGACTTTCTGGAAGCCAAGGGCTACAAAGAAGTGAAAAAGGACCTGATCATCAAGCTGCCTAAGTACTCCCTGTTCGAGCTGGAAAACGGCCGGAAGAGAATGCTGGCCTCTGCCGGCGAACTGCAGAAGGGAAACGAACTGGCCCTGCCCTCCAAATATGTGAACTTCCTGTACCTGGCCAGCCACTATGAGAAGCTGAAGGGCTCCCCCGAGGATAATGAGCAGAAACAGCTGTTTGTGGAACAGCACAAGCACTACCTGGACGAGATCATCGAGCAGATCAGCGAGTTCTCCAAGAGAGTGATCCTGGCCGACGCTAATCTGGACAAAGTGCTGTCCGCCTACAACAAGCACCGGGATAAGCCCATCAGAGAGCAGGCCGAGAATATCATCCACCTGTTTACCCTGACCAATCTGGGAGCCCCTGCCGCCTTCAAGTACTTTGACACCACCATCGACCGGAAGCAGTACAGGAGCACCAAAGAGGTGCTGGACGCCACCCTGATCCACCAGAGCATCACCGGCCTGTACGAGACACGGATCGACCTGTCTCAGCTGGGAGGCGAC**ACTAGTTCCGGCGGCAGCGCTTCTCCAAAGCGTCCGCGTGACCGTCACGATGGAGAATTGGGTGGACGCAAACGTGCAAGAGGTTAAGGTACCTGATTGATCGATAGAGCTCGAATTTCCCCGATCGTTCAAACATTTGGCAATAAAGTTTCTTAAGATTGAATCCTGTTGCCGGTCTTGCGATGATTATCATATAATTTCTGTTGAATTACGTTAAGCATGTAATAATTAACATGTAATGCATGACGTTATTTATGAGATGGGTTTTTATGATTAGAGTCCCGCAATTATACATTTAATACGCGATAGAAAACAAAATATAGCGCGCAAACTAGGATAAATTATCGCGCGCGGTGTCATCTATGTTACTAGATCGG

pKKH-SaCas9

GGATCATGAACCAACGGCCTGGCTGTATTTGGTGGTTGTGTAGGGAGATGGGGAGAAGAAAAGCCCGATTCTCTTCGCTGTGATGGGCTGGATGCATGCGGGGGAGCGGGAGGCCCAAGTACGTGCACGGTGAGCGGCCCACAGGGCGAGTGTGAGCGCGAGAGGCGGGAGGAACAGTTTAGTACCACATTGCCCAGCTAACTCGAACGCGACCAACTTATAAACCCGCGCGCTGTCGCTTGTGTgagaccATCTAGACggtctcTGTTTTAGTACTCTGGAAACAGAATCTACTAAAACAAGGCAAAATGCCGTGTTTATCTCGTCAACTTGTTGGCGAGATTTTTTTTTGTTTTTTATGTCTCCCGGGTGCAGCGTGACCCGGTCGTGCCCCTCTCTAGAGATAATGAGCATTGCATGTCTAAGTTATAAAAAATTACCACATATTTTTTTTGTCACACTTGTTTGAAGTGCAGTTTATCTATCTTTATACATATATTTAAACTTTACTCTACGAATAATATAATCTATAGTACTACAATAATATCAGTGTTTTAGAGAATCATATAAATGAACAGTTAGACATGGTCTAAAGGACAATTGAGTATTTTGACAACAGGACTCTACAGTTTTATCTTTTTAGTGTGCATGTGTTCTCCTTTTTTTTTGCAAATAGCTTCACCTATATAATACTTCATCCATTTTATTAGTACATCCATTTAGGGTTTAGGGTTAATGGTTTTTATAGACTAATTTTTTTAGTACATCTATTTTATTCTATTTTAGCCTCTAAATTAAGAAAACTAAAACTCTATTTTAGTTTTTTTATTTAATAATTTAGATATAAAATAGAATAAAATAAAGTGACTAAAAATTAAACAAATACCCTTTAAGAAATTAAAAAAACTAAGGAAACATTTTTCTTGTTTCGAGTAGATAATGCCAGCCTGTTAAACGCCGTCGACGAGTCTAACGGACACCAACCAGCGAACCAGCAGCGTCGCGTCGGGCCAAGCGAAGCAGACGGCACGGCATCTCTGTCGCTGCCTCTGGACCCCTATCGAGAGTTCCGCTCCACCGTTGGACTTGCTCCGCTGTCGGCATCCAGAAATTGCGTGGCGGAGCGGCAGACGTGAGCCGGCACGGCAGGCGGCCTCCTCCTCCTCTCACGGCACGGCAGCTACGGGGGATTCCTTTCCCACCGCTCCTTCGCTTTCCCTTCCTCGCCCGCCGTAATAAATAGACACCCCCTCCACACCCTCTTTCCCCAACCTCGTGTTGTTCGGAGCGCACACACACACAACCAGATCTCCCCCAAATCCACCCGTCGGCACCTCCGCTTCAAGGTACGCCGCTCGTCCTCCCCCCCCCCCCCTCTCTACCTTCTCTAGATCGGCGTTCCGGTCCATGGTTAGGGCCCGGTAGTTCTACTTCTGTTCATGTTTGTGTTAGATCCGTGTTTGTGTTAGATCCGTGCTGCTAGCGTTCGTACACGGATGCGACCTGTACGTCAGACACGTTCTGATTGCTAACTTGCCAGTGTTTCTCTTTGGGGAATCCTGGGATGGCTCTAGCCGTTCCGCAGACGGGATCGATTTCATGATTTTTTTTGTTTCGTTGCATAGGGTTTGGTTTGCCCTTTTCCTTTATTTCAATATATGCCGTGCACTTGTTTGTCGGGTCATCTTTTCATGCTTTTTTTTGTCTTGGTTGTGATGATGTGGTCTGGTTGGGCGGTCGTTCTAGATCGGAGTAGAATTCTGTTTCAAACTACCTGGTGGATTTATTAATTTTGGATCTGTATGTGTGTGCCATACATATTCATAGTTACGAATTGAAGATGATGGATGGAAATATCGATCTAGGATAGGTATACATGTTGATGCGGGTTTTACTGATGCATATACAGAGATGCTTTTTGTTCGCTTGGTTGTGATGATGTGGTGTGGTTGGGCGGTCGTTCATTCGTTCTAGATCGGAGTAGAATACTGTTTCAAACTACCTGGTGTATTTATTAATTTTGGAACTGTATGTGTGTGTCATACATCTTCATAGTTACGAGTTTAAGATGGATGGAAATATCGATCTAGGATAGGTATACATGTTGATGTGGGTTTTACTGATGCATATACATGATGGCATATGCAGCATCTATTCATATGCTCTAACCTTGAGTACCTATCTATTATAATAAACAAGTATGTTTTATAATTATTTTGATCTTGATATACTTGGATGATGGCATATGCAGCAGCTATATGTGGATTTTTTTAGCCCTGCCTTCATACGCTATTTATTTGCTTGGTACTGTTTCTTTTGTCGATGCTCACCCTGTTGTTTGGTGTTACTTCTGCAGCCATGGCTCCTAAGAAAAAGAGGAAGGTGGCG**AAGCGGAACTACATCCTGGGCCTGGACATCGGCATCACCAGCGTGGGCTACGGCATCATCGACTACGAGACACGGGACGTGATCGATGCCGGCGTGCGGCTGTTCAAAGAGGCCAACGTGGAAAACAACGAGGGCAGGCGGAGCAAGAGAGGCGCCAGAAGGCTGAAGCGGCGGAGGCGGCATAGAATCCAGAGAGTGAAGAAGCTGCTGTTCGACTACAACCTGCTGACCGACCACAGCGAGCTGAGCGGCATCAACCCCTACGAGGCCAGAGTGAAGGGCCTGAGCCAGAAGCTGAGCGAGGAAGAGTTCTCTGCCGCCCTGCTGCACCTGGCCAAGAGAAGAGGCGTGCACAACGTGAACGAGGTGGAAGAGGACACCGGCAACGAGCTGTCCACCAAAGAGCAGATCAGCCGGAACAGCAAGGCCCTGGAAGAGAAATACGTGGCCGAACTGCAGCTGGAACGGCTGAAGAAAGACGGCGAAGTGCGGGGCAGCATCAACAGATTCAAGACCAGCGACTACGTGAAAGAAGCCAAACAGCTGCTGAAGGTGCAGAAGGCCTACCACCAGCTGGACCAGAGCTTCATCGACACCTACATCGACCTGCTGGAAACCCGGCGGACCTACTATGAGGGACCTGGCGAGGGCAGCCCCTTCGGCTGGAAGGACATCAAAGAATGGTACGAGATGCTGATGGGCCACTGCACCTACTTCCCCGAGGAACTGCGGAGCGTGAAGTACGCCTACAACGCCGACCTGTACAACGCCCTGAACGACCTGAACAATCTCGTGATCACCAGGGACGAGAACGAGAAGCTGGAATATTACGAGAAGTTCCAGATCATCGAGAACGTGTTCAAGCAGAAGAAGAAGCCCACCCTGAAGCAGATCGCCAAAGAAATCCTCGTGAACGAAGAGGATATTAAGGGCTACAGAGTGACCAGCACCGGCAAGCCCGAGTTCACCAACCTGAAGGTGTACCACGACATCAAGGACATTACCGCCCGGAAAGAGATTATTGAGAACGCCGAGCTGCTGGATCAGATTGCCAAGATCCTGACCATCTACCAGAGCAGCGAGGACATCCAGGAAGAACTGACCAATCTGAACTCCGAGCTGACCCAGGAAGAGATCGAGCAGATCTCTAATCTGAAGGGCTATACCGGCACCCACAACCTGAGCCTGAAGGCCATCAACCTGATCCTGGACGAGCTGTGGCACACCAACGACAACCAGATCGCTATCTTCAACCGGCTGAAGCTGGTGCCCAAGAAGGTGGACCTGTCCCAGCAGAAAGAGATCCCCACCACCCTGGTGGACGACTTCATCCTGAGCCCCGTCGTGAAGAGAAGCTTCATCCAGAGCATCAAAGTGATCAACGCCATCATCAAGAAGTACGGCCTGCCCAACGACATCATTATCGAGCTGGCCCGCGAGAAGAACTCCAAGGACGCCCAGAAAATGATCAACGAGATGCAGAAGCGGAACCGGCAGACCAACGAGCGGATCGAGGAAATCATCCGGACCACCGGCAAAGAGAACGCCAAGTACCTGATCGAGAAGATCAAGCTGCACGACATGCAGGAAGGCAAGTGCCTGTACAGCCTGGAAGCCATCCCTCTGGAAGATCTGCTGAACAACCCCTTCAACTATGAGGTGGACCACATCATCCCCAGAAGCGTGTCCTTCGACAACAGCTTCAACAACAAGGTGCTCGTGAAGCAGGAAGAAAACAGCAAGAAGGGCAACCGGACCCCATTCCAGTACCTGAGCAGCAGCGACAGCAAGATCAGCTACGAAACCTTCAAGAAGCACATCCTGAATCTGGCCAAGGGCAAGGGCAGAATCAGCAAGACCAAGAAAGAGTATCTGCTGGAAGAACGGGACATCAACAGGTTCTCCGTGCAGAAAGACTTCATCAACCGGAACCTGGTGGATACCAGATACGCCACCAGAGGCCTGATGAACCTGCTGCGGAGCTACTTCAGAGTGAACAACCTGGACGTGAAAGTGAAGTCCATCAATGGCGGCTTCACCAGCTTTCTGCGGCGGAAGTGGAAGTTTAAGAAAGAGCGGAACAAGGGGTACAAGCACCACGCCGAGGACGCCCTGATCATTGCCAACGCCGATTTCATCTTCAAAGAGTGGAAGAAACTGGACAAGGCCAAAAAAGTGATGGAAAACCAGATGTTCGAGGAAAAGCAGGCCGAGAGCATGCCCGAGATCGAAACCGAGCAGGAGTACAAAGAGATCTTCATCACCCCCCACCAGATCAAGCACATTAAGGACTTCAAGGACTACAAGTACAGCCACCGGGTGGACAAGAAGCCTAATAGAAAGCTGATTAACGACACCCTGTACTCCACCCGGAAGGACGACAAGGGCAACACCCTGATCGTGAACAATCTGAACGGCCTGTACGACAAGGACAATGACAAGCTGAAAAAGCTGATCAACAAGAGCCCCGAAAAGCTGCTGATGTACCACCACGACCCCCAGACCTACCAGAAACTGAAGCTGATTATGGAACAGTACGGCGACGAGAAGAATCCCCTGTACAAGTACTACGAGGAAACCGGGAACTACCTGACCAAGTACTCCAAAAAGGACAACGGCCCCGTGATCAAGAAGATTAAGTATTACGGCAACAAACTGAACGCCCATCTGGACATCACCGACGACTACCCCAACAGCAGAAACAAGGTCGTGAAGCTGTCCCTGAAGCCCTACAGATTCGACGTGTACCTGGACAATGGCGTGTACAAGTTCGTGACCGTGAAGAATCTGGATGTGATCAAAAAAGAAAACTACTACGAAGTGAATAGCAAGTGCTATGAGGAAGCTAAGAAGCTGAAGAAGATCAGCAACCAGGCCGAGTTTATCGCCTCCTTCTACAAGAACGATCTGATCAAGATCAACGGCGAGCTGTATAGAGTGATCGGCGTGAACAACGACCTGCTGAACCGGATCGAAGTGAACATGATCGACATCACCTACCGCGAGTACCTGGAAAACATGAACGACAAGAGGCCCCCCCACATCATTAAGACAATCGCCTCCAAGACCCAGAGCATTAAGAAGTACAGCACAGACATTCTGGGCAACCTGTATGAAGTGAAATCTAAGAAGCACCCTCAGATCATCAAAAAGGG**ACTAGTTCCGGCGGCAGCGCTTCTCCAAAGCGTCCGCGTGACCGTCACGATGGAGAATTGGGTGGACGCAAACGTGCAAGAGGTTAAGGTACCTGATTGATCGATAGAGCTCGAATTTCCCCGATCGTTCAAACATTTGGCAATAAAGTTTCTTAAGATTGAATCCTGTTGCCGGTCTTGCGATGATTATCATATAATTTCTGTTGAATTACGTTAAGCATGTAATAATTAACATGTAATGCATGACGTTATTTATGAGATGGGTTTTTATGATTAGAGTCCCGCAATTATACATTTAATACGCGATAGAAAACAAAATATAGCGCGCAAACTAGGATAAATTATCGCGCGCGGTGTCATCTATGTTACTAGATCGG


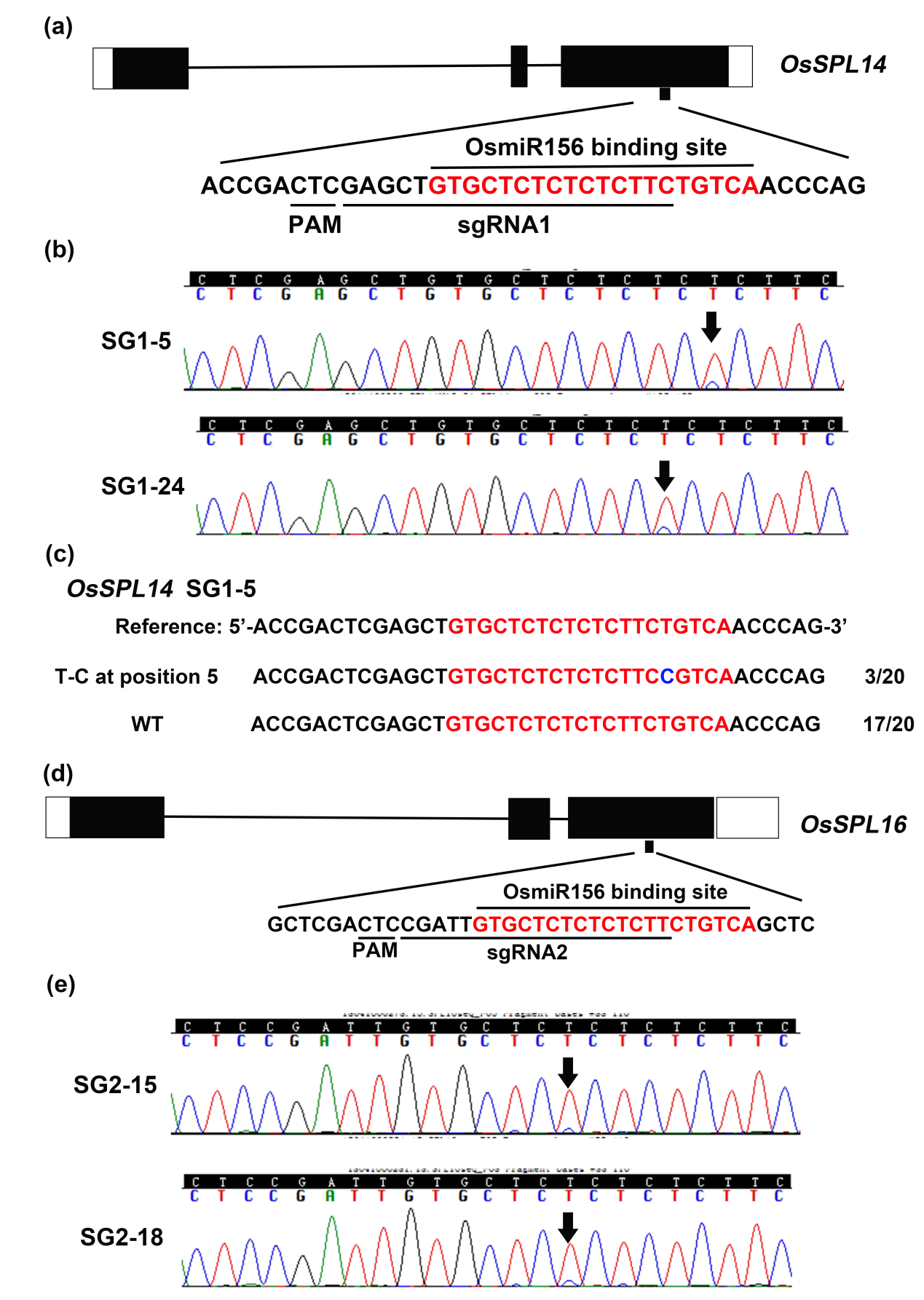


**Figure S1.** Base editing by ABE-P1 at the genomic sequences with NAG PAMs.

(a) Schematic view of the sgRNA1 target site with an NAG PAM sequence in the *OsSPL14* gene. The OsmiR156 binding sequence of *OsSPL14* is highlighted in red.

(b) Sequencing chromatograms of two edited lines, SG1-5 and SG1-24, are shown. Arrows point to the positions with edited bases.

(c) TA cloning results for the edited line SG1-5. Twenty clones were randomly selected for sequencing. The target site is highlighted in red and the edited base is marked by a blue letter.

(d) Schematic view of the sgRNA2 target site with an NAG PAM sequence in the *OsSPL16* gene. The OsmiR156 binding sequence of *OsSPL16* is highlighted in red.

(e) Two lines, SG2-15 and SG2-18 are edited at the *OsSPL16* target site. Their sequencing chromatograms at the *OsSPL16* target site are shown and the edited bases are marked by arrows.


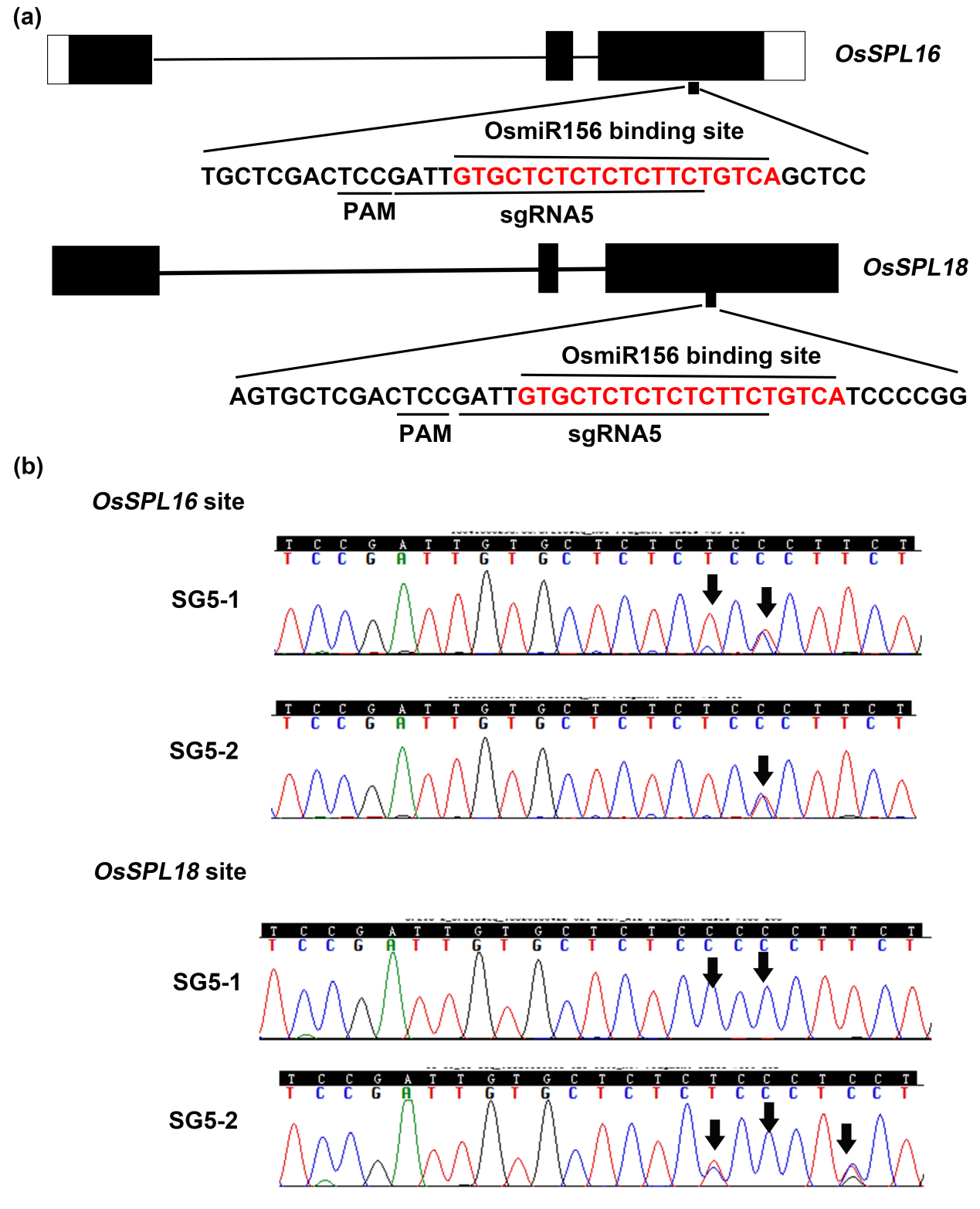


**Figure S2.** Targeted base editing at *OsSPL16* and *OsSPL18* by ABE-P3.

(a) Targeted editing of *OsSPL16* and *OsSPL18* simultaneously by sgRNA5. The OsmiR156 binding sequences in *OsSPL16* and *OsSPL18* are highlighted in red.

(b) Two representative lines, SG5-1 and SG5-2, are edited at both target sites. Their sequencing chromatograms are shown and the edited bases are marked by arrows.


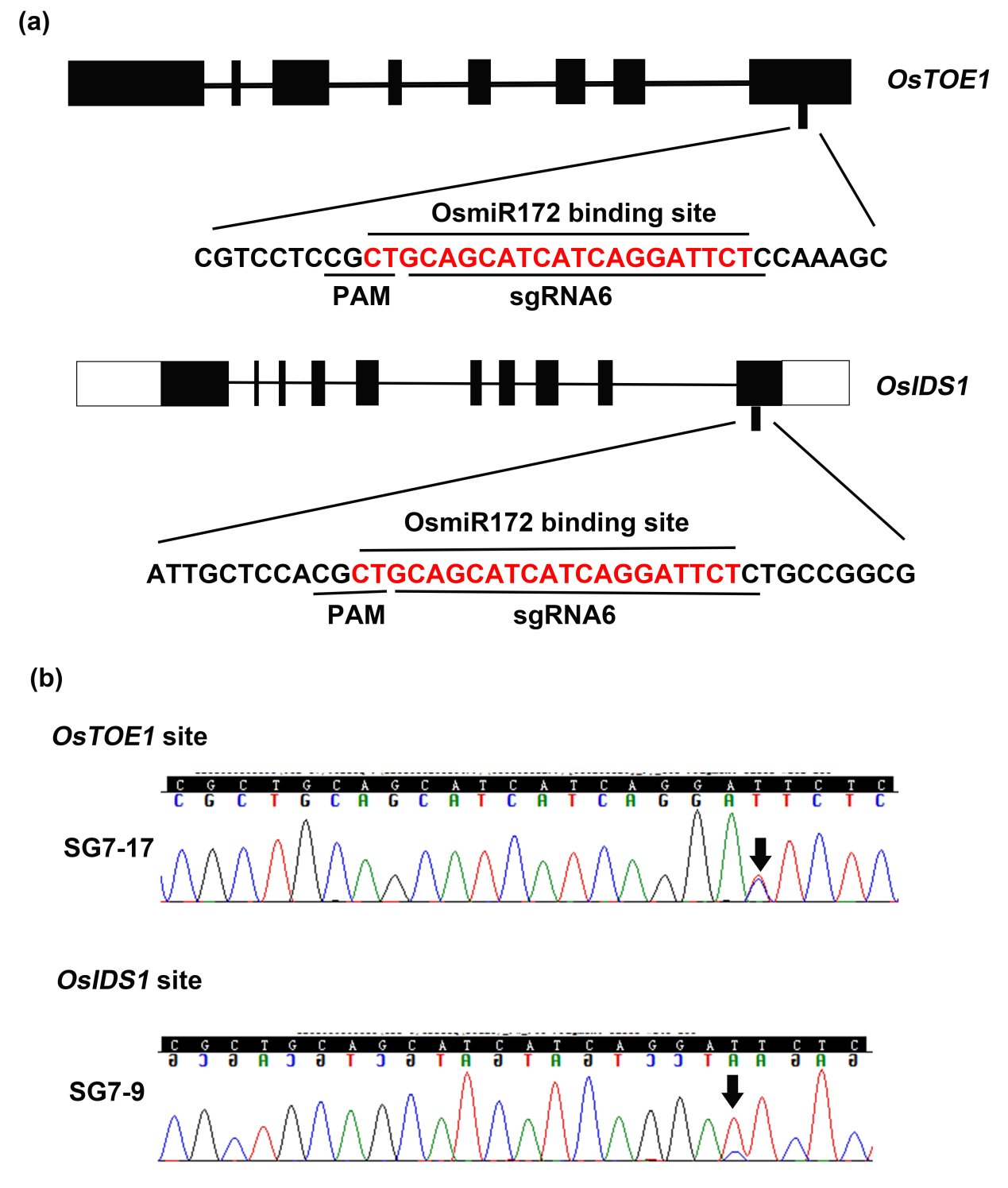


**Figure S3.** Targeted base editing at *OsTOE1* and *OsIDS1* by ABE-P4.

(a) *OsTOE1* and *OsIDS1* are simultaneously targeted by sgRNA6. The OsmiR172 binding sequences in *OsTOE1* and *OsIDS1* are highlighted in red. (b) Line SG7-17 shows base editing at the *OsTOE1* target site, whereas line SG7-9 shows base editing at the *OsIDS1* target site. The sequencing chromatograms at corresponding edited sites are shown and the edited bases are marked by arrows.


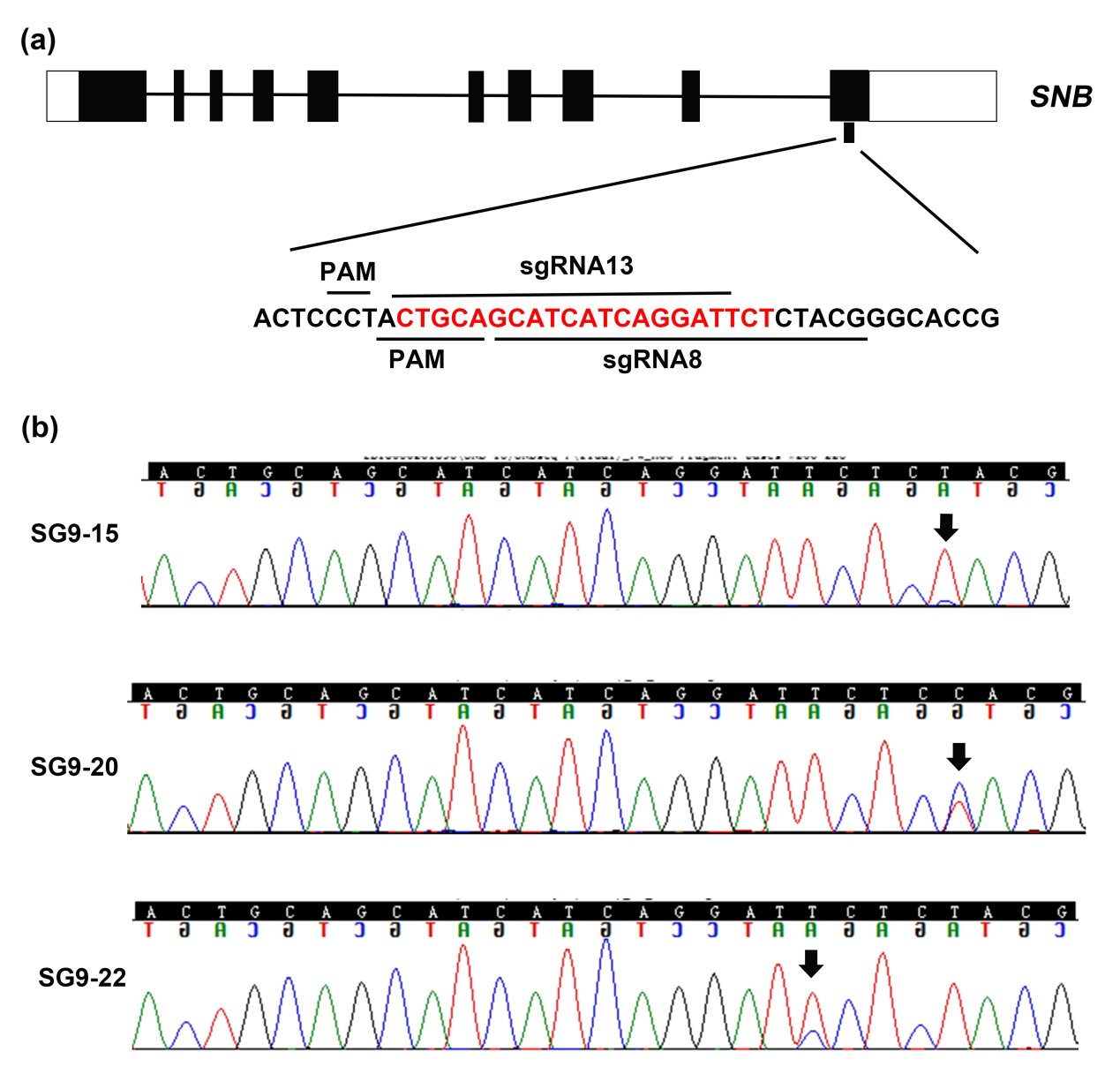


**Figure S4.** Targeted base editing at the *SNB* gene by ABE-P5.

(a) Schematic view of the sgRNA8 and sgRNA13 target sites in the *SNB* gene. The OsmiR172 binding sequence of *SNB* is highlighted in red letters.

(b) Sequencing chromatograms of three edited lines, SG9-15, SG9-20, SG9-22, are shown. Arrows point to the positions with edited bases.


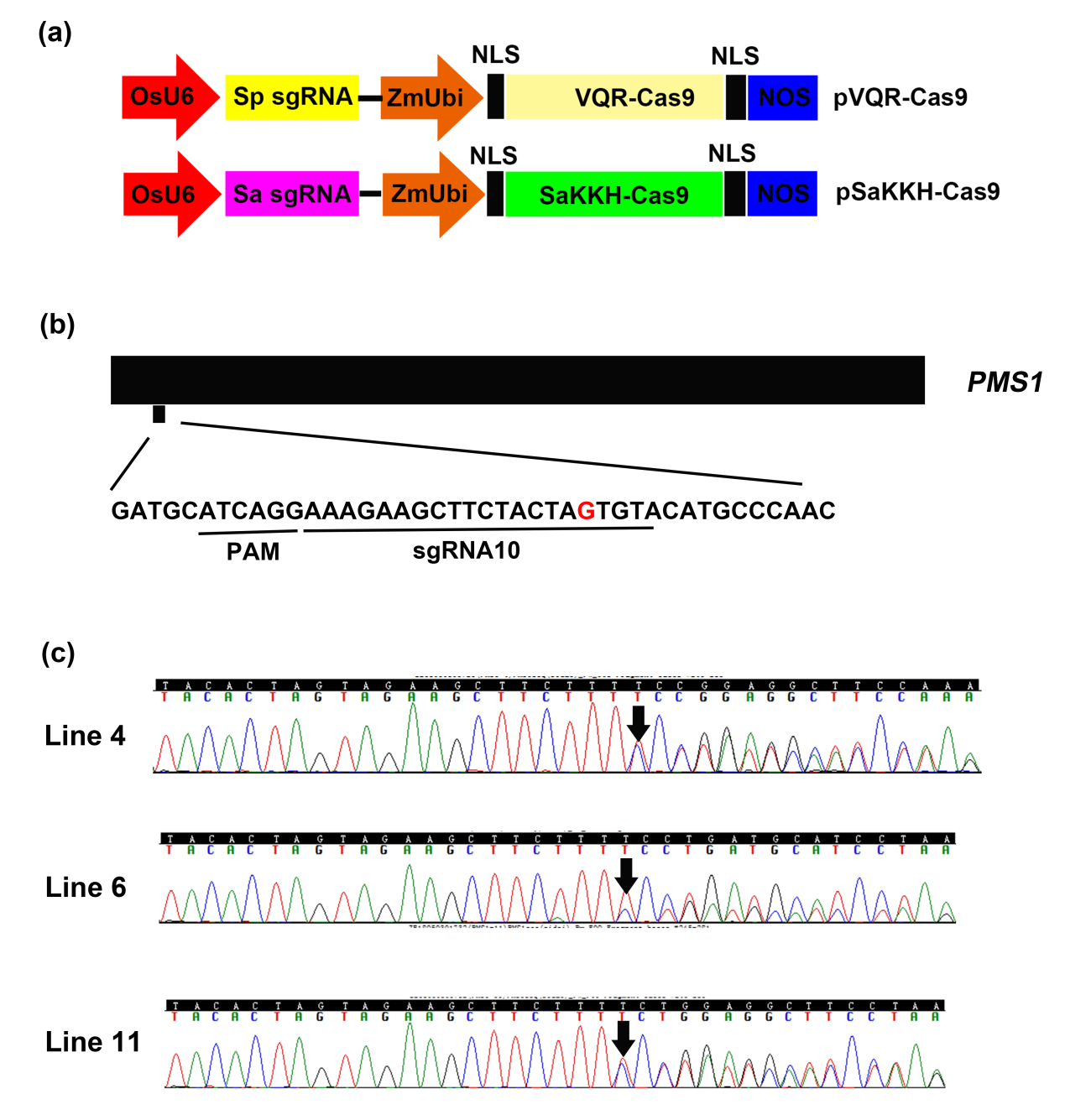


**Figure S5.** Targeted mutation at the *PMS1* and *PMS3* genes by pSaKKH-Cas9 and pVQR-Cas9.

1. Schematic view of pSaKKH-Cas9 and pVQR-Cas9 vectors.

(b) Schematic view of the sgRNA10 target site in the *PMS1* gene. The targeted G for base editing is highlighted in red.

(c) Three lines are mutated at the *PMS1* gene by the pSaKKH-Cas9 vector. Their sequencing chromatograms are shown and the sites of mutations are marked by arrows.

**Table S1.** Primers for sgRNAs used in this study.

| sgRNA | Primer name | Primer sequence 5’-3’ |
| --- | --- | --- |
| sgRNA1 | sgRNA1-F | TGTGGAAGAGAGAGAGCACAGCTC |
|  | sgRNA1-R | AAACGAGCTGTGCTCTCTCTCTTC |
| sgRNA2 | sgRNA2-F | TGTGGAAGAGAGAGAGCACAATCG |
|  | sgRNA2-R | AAACCGATTGTGCTCTCTCTCTTC |
| sgRNA3 | sgRNA3-F | TGTGCAAGAAAGCCTGTGGAAACG |
|  | sgRNA3-R | AAACCGTTTCCACAGGCTTTCTTG |
| sgRNA4 | sgRNA4-F | TGTGAGAAGAGAGAGAGCACAGCT |
|  | sgRNA4-R | AAACAGCTGTGCTCTCTCTCTTCT |
| sgRNA5 | sgRNA5-F | TGTGAGAAGAGAGAGAGCACAATC |
|  | sgRNA5-R | AAACGATTGTGCTCTCTCTCTTCT |
| sgRNA6 | sgRNA6-F | TGTGGAGAATCCTGATGATGCTGC |
|  | sgRNA6-R | AAACGCAGCATCATCAGGATTCTC |
| sgRNA7 | sgRNA7-F | TGTGAGAAGCAGGTCACGTGCGCT |
|  | sgRNA7-R | AAACAGCGCACGTGACCTGCTTCT |
| sgRNA8 | sgRNA8-F | TGTGTGCGTAGAGAATCCTGATGATGC |
|  | sgRNA8-R | AAACGCATCATCAGGATTCTCTACGCA |
| sgRNA9 | sgRNA9-F | TGTGTGACAGAAGAGAGGGAGCATGC |
|  | sgRNA9-R | AAACGCATGCTCCCTCTCTTCTGTCA |
| sgRNA10 | sgRNA10-F | TGTGTGTACACTAGTAGAAGCTTCTTT |
|  | sgRNA10-R | AAACAAAGAAGCTTCTACTAGTGTA CA |
| sgRNA11 | sgRNA11-F | TGTGTGTTTGTCTACCATCCATCA |
|  | sgRNA11-R | AAACTGATGGATGGTAGACAAACA |
| sgRNA12 | sgRNA12-F | TGTGTGACAGAAGAGAGAGAGCACAGC |
|  | sgRNA12-R | AAACGCTGTGCTCTCTCTCTTCTGT CA |
| sgRNA13 | sgRNA13-F | TGTGAATCCTGATGATGCTGCAGT |
|  | sgRNA13-R | AAACACTGCAGCATCATCAGGATT |

**Table S2.** Primers for target sites amplification and sequencing.

| Primer name | Primer sequence 5’-3’ | Purpose |
| --- | --- | --- |
| SPL14-F | AGGGTTCCAAGCAGCGTAAGGA | Amplify all the *OsSPL14* target sites |
| SPL14-R | TGGTGCTGGGGCTGGACCGTTC |  |
| SPL14seq-F | TCTCCGGTGGTATCCAGTGGCAC | Sequencing Primer |
| SPL17-F | GGGTTCCAAGCAGTGTGAGGGA | Amplify the *OsSPL17* target site |
| SPL17-R | GGACCTCGTTCAGCACAACCC |  |
| SPL17seq-F | ATGAACTCCATCCTCACCGCAG | Sequencing Primer |
| SPL16-F | TTGGCATCAGCAGCAGGCAGCAG | Amplify all the *OsSPL16* target sites |
| SPL16-R | AGAGACGACGACGTGCCATCCGATG |  |
| SPL16seq-F | CAGCTGCTGCTGCTGCTTCAGTGTG | Sequencing Primer |
| SPL18-F | TGGGATCATCAAATCCGAGGAG | Amplify all the *OsSPL18* target sites |
| SPL18-R | CTGTCCATGCTCGGGCAGGCG |  |
| SPL18seq-F | CCTCCATGAGGGCGATCAGATA | Sequencing Primer |
| GRF4-F | GCATATGGATCGGATATGGACTTC | Amplify the *GRF4* target site |
| GRF4-R | TGAACAATCACCTGAGATCTTTGC |  |
| GRF4seq-F | GTAACACGGTTGCTACGATGTGC | Sequencing Primer |
| OsIDS1-F | AGAATATGTACTGTTCCAACTGCAG | Amplify the *OsIDS1* target site |
| OsIDS1-R | TCAAGCTCCATGCCCATGATGAATT |  |
| OsIDS1seq-F | GTCAATGCACCTGGCTAGTACT | Sequencing Primer |
| OsTOE1-F | ACTGTTTAGTAATCTCAGTAAC | Amplify the *OsTOE1*target site |
| OsTOE1-R | TTGAATCGTCGCAAATCCAATCCG |  |
| OsTOE1seq-F | GCAAATACAGTCATGAATCC | Sequencing Primer |
| OMTN1-F | CCGGGAGATCTTCAGGGGGA | Amplify the *OMTN1* target site |
| OMTN1-R | GCATACACACTTCGTTCTCTT |  |
| OMTN1seq-F | TCGTGCACCATGCCCGCTC | Sequencing Primer |
| SNB-F | GGTTAGAATCATCCATTCATG | Amplify all the *SNB* target sites |
| SNB-R | CCAGCTTTAGTGCCCACTGTTC |  |
| SNBseq-F | CTGGTAGCACGGTCACGATTCG | Sequencing Primer |
| OsSPL13-F | ATTTGACAAGAGTGCATTATGCA | Amplify the *OsSPL13* target site |
| OsSPL13-R | AGCACTGCATATATGTAGCAGCAG |  |
| OsSPL13seq-F | GCTTCATCATCATCTTGTCC | Sequencing Primer |
| PMS1-F | GAAACCACGCAAGCTTGGCTC | Amplify the *PMS1*target site |
| PMS1-R | CACGTGCACGCTAACTAGTATA |  |
| PMS1seq-F | CCGAATTAACCTGGCATAGA | Sequencing Primer |
| PMS3-F | GATGCACATACTGAATCTCT | Amplify the *PMS3* target site |
| PMS3-R | GTGGTACCTCGTCAAGCGACAC |  |
| PMS3seq-F | GATGAGCAACATGAGAACTTC | Sequencing Primer |
